# One-dimensional CsPbBr_3_ superlattices with polarized and amplified spontaneous circularly polarized emissions

**DOI:** 10.1038/s41467-026-73513-2

**Published:** 2026-05-23

**Authors:** Baowei Zhang, Kexin Chen, Zhengkun Xie, Kun Hu, Haiyun Dong, Yong Sheng Zhao, Liberato Manna, Siyu Lu

**Affiliations:** 1 College of Chemistry, Pingyuan Laboratory, Zhengzhou University, Zhengzhou, China; 2 Beijing National Laboratory for Molecular Sciences, Institute of Chemistry, Chinese Academy of Sciences, Beijing, China; 3 School of Chemical Sciences, University of Chinese Academy of Sciences, Beijing, China; 4 Nanochemistry, Istituto Italiano di Tecnologia, Genova, Italy

**Keywords:** Molecular self-assembly, Metamaterials

## Abstract

Nanocrystal superlattices typically occur in two- or three-dimensional configurations, constrained to the micrometer scale and with limited size tunability. Here, we report one-dimensional superlattices prepared by self-assembly of CsPbBr_3_ nanorods and nanoplatelets. These exhibit a hierarchical structure, evolving from ribbons (µm) of nanorods or nanoplatelets to assemblies of ribbons (mm). These superlattices have a diameter of 0.8–1 µm and length in the range of 13–1500 µm, and their aspect ratio can be tuned from 14 to 1200 by adjusting the shape of the nanorods and nanoplatelets. Thanks to their anisotropic structure, the superlattices show strong polarized emission with a near-unity degree of polarization, ≈4 times larger than that of randomly assembled film. These superlattices also exhibit chiral optical response (circular dichroism and circularly polarized emission). Since no chiral ligands are used in the synthesis, the chiral signal (negative or positive) from the superlattices is random. However, the signal can be controlled after the addition of chiral ligands. The maximum dissymmetry factor of the luminescence (*g*_lum_) is −0.11, and can be further amplified to −0.32 in the amplified spontaneous emission of the superlattices.

## Abstract (web summary)

Nanocrystal superlattices typically occur in two- and three-dimensional configurations, constrained to the micrometer scale and with limited size tunability. Here, the authors assemble CsPbBr3 nanoplatelets into chiroptically active 1D helical superlattices with mm lengths and μm widths.

## Introduction

Halide perovskite nanocrystals have drawn significant attention not only because of the bright emission from individual nanocrystals^1,2^, but also since they exhibit collective optical properties when assembled into superlattices^3–7^. For example, the electronic coupling between the constituting nanocrystals when they are organized in superlattices can induce the formation of minibands (narrow secondary energy bands arising from interparticle electronic hybridization and energy splitting) structures and result in a redshifted photoluminescence^3,8^. Also, after interacting coherently with light, the CsPbBr_3_ nanocrystal superlattices can emit short and intense bursts of light (so-called superfluorescence), a process that is a consequence of the long-range order of the nanocrystals in these superlattices^4,9,10^. The overall configuration of perovskite nano-crystal superlattices can significantly influence their collective properties^6,7,11,12^. Three-dimensional (3D) superlattices are the most studied cases for superfluorescence^4,13^. Most 3D superlattices have cuboid shapes^12,14,15^, while a few of them are spherical^16^, rhombic-prism^11^ or pyramidal^13^. Two-dimensional (2D) superlattices typically exhibit a µm-scale in the lateral direction and only a few layers of nanocrystals in the thickness direction^6,17–20^. These 2D superlattices exhibit strongly polarized emission and thus may enhance the light outcoupling efficiency in light-emitting diodes (LEDs)^6^. So far, there have been only few reports on one-dimensional (1D) superlattices from perovskite nanocrystals^21–26^. It is expected that using building blocks different from cubes (the typical shape of CsPbBr_3_ nanocrystals) may confer more structural flexibility and even chirality to the resulting 1D superlattices^27,28^.

**Fig. 1:**
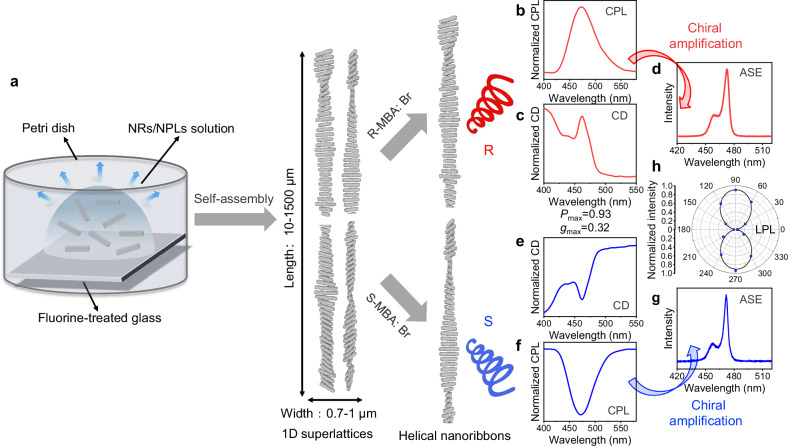
Preparation of one-dimensional superlattices exhibiting polarized emission, circularly polarized emission and amplified spontaneous emission. **a** Schematic illustration of the self-assembly of CsPbBr₃ NRs/NPLs into one-dimensional superlattices. **b** Circularly polarized luminescence spectra, (**c**) circular dichroism, and (**d**) amplified spontaneous emission spectra of the R-superlattices (R-SLs). **e** Circular dichroism, (**f**) circularly polarized luminescence spectra, and (**g**) amplified spontaneous emission spectra of the S-superlattices (S-SLs). **h** Polar plot of the photoluminescence intensity for 1D NPLs-DDAB-2 superlattices. All the intensities plotted in (**b**–**h**) were normalized. The red curves represent the R-SLs, while the blue curves represent the S-SLs. LPL Linearly polarized luminescence, ASE Amplified spontaneous emission. Fluorine-treated: The glass substrates are treated with perfluorodecyltriethoxysilane. Source data are provided as a Source Data file.

We report here the self-assembly of CsPbBr_3_ nanorods (NRs) and nanoplatelets (NPLs) for the preparation of 1D superlattices having a width in the ≈0.8–1 µm range and a length in the ≈13–1500 µm range (Fig. 1a). Structural analysis revealed a hierarchical organization of the superlattices: the NRs or NPLs (nm in lateral size) firstly stack into ribbons (µm in length), then the ribbons align with each other to form 1D superlattices (mm in length). The shape of the NRs/NPLs influences the aspect ratio of the superlattices, which spans the 14–1200 range. Due to their anisotropic geometry, the superlattices exhibit strong polarized emission, with a maximum degree of polarization (*P*) close to near unity, a value that is significantly larger than that observed from randomly assembled CsPbBr_3_ films (Fig. 1h). Also, the ribbons in the superlattices have a chiral configuration, which we ascribe to the propagation of an orientational twist of these NRs/NPLs perpendicular to their stacking direction. This configuration results in strong circular dichroism and circularly polarized emission of the 1D superlattices (Fig. 1b–f). The signal (negative or positive) of the circular dichroism can be controlled by exchanging the native ligands on the surface of the NRs/NPLs with chiral ones. The maximum dissymmetry factor (*g*_lum_) from these 1D superlattices is −0.11^29–32^. The chirality can be further amplified in the amplified spontaneous emission from the 1D superlattice, up to a *g*_lum_ of −0.32 (Fig. 1d, g).

## Result and discussion

### Preparation of 1D superlattices from NRs/NPLs

The experimental details on the syntheses and assembly can be found in the Methods section. Briefly, CsPbBr_3_ NRs with different diameter and length were prepared using didodecylamine (DDDAm) and oleic acid as surfactants^33^. By controlling the reaction temperature, the diameter of the NRs could be tuned from 7.4 to 8.3 nm, while preserving an average length size of ≈40 nm, as determined by transmission electron microscopy (TEM, Supplementary Fig. 1). All the samples had pure orthorhombic CsPbBr_3_ phase (ICSD, #97851) as seen from their X-ray diffraction patterns (XRD, Supplementary Fig. 3)^34^.

**Fig. 2:**
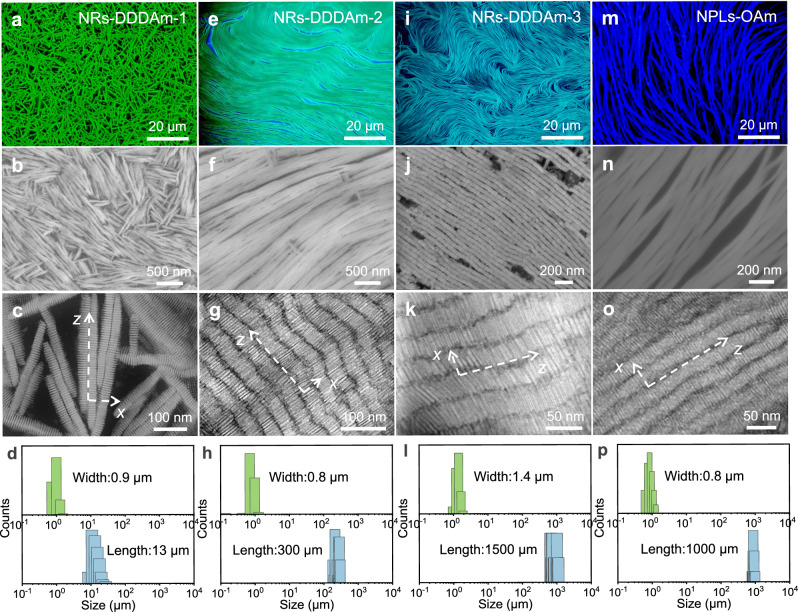
One-dimensional (1D) superlattices built by assembly of CsPbBr_3_ NRs or NPLs of different aspect ratios. **a** Optical microscope image of the NRs-DDDAm-1 (*d* = 8.3 nm) 1D superlattices under 365 nm light excitation. **b** SEM image of the superlattices at µm scale. **c** STEM image of the superlattices at nm scale. **d** Statistical counting of the length and width of the superlattices. (NRs-DDDAm-1: *n* = 100 for both width and length.) **e**–**h** Same characterization as in (**a**–**d**) but for the NRs-DDDAm-2 (*d* = 7.8 nm) superlattices. (NRs-DDDAm-2: *n* = 100 for width, *n* = 30 for length.). **i**–**l** Same characterization as in (**a**–**d**) but for the NRs-DDDAm-3 (*d* = 7.4 nm) superlattices. (NRs-DDDAm-3: *n* = 100 for width, *n* = 15 for length.) **m**–**p** Same characterization but for the NPLs-OAm (*d* = 4.6 nm) assembled superlattices. (NPLs-OAm: *n* = 100 for width, *n* = 15 for length.). The green bars represent the width distribution, and blue bars represent the length distribution. Source data are provided as a Source Data file.

After the synthesis, the NRs were dispersed in toluene (10 mg mL^−1^) for the assembly experiments. The glass substrates were treated with perfluorodecyltriethoxysilane to enhance their interaction with the NRs solution (Supplementary Fig. 4). The NRs solution was dropped on a glass substrate positioned in the container of a Petri dish, which was then covered by the lid (Supplementary Fig. 5). During the 1-2 h evaporation time, the 1D superlattices were formed. As shown in Fig. 2a–d, in this sample (named as NRs-DDDAm-1) the individual NR had a diameter of ≈8.3 nm. As seen under the optical microscope (Fig. 2a), the NRs formed fibrillar-like assemblies with an average length of ≈13 µm and width of ≈0.9 µm (aspect ratio (AR) = 14, Fig. 2d). Scanning electron microscopy (SEM) revealed that each fibril was comprised of clusters of ribbons (Fig. 2b). Scanning transmission electron microscopy (STEM) confirmed that each individual ribbon was the result of edge-up stacking of NRs (Fig. 2c)^22^. This multi-scale morphology characterization thus revealed a hierarchical structure of the 1D superlattices.

The NRs-DDDAm-2 sample, consisting of NRs with a diameter of 7.8 nm, also formed 1D superlattices, this time with an average length of ≈300 µm and width of ≈0.8 µm (Fig. 2e, h). These superlattices, on average, had a large aspect ratio (AR) of 375 and exhibited a “wavy” configuration (Fig. 2e). SEM and TEM analyses confirmed that these superlattices as well had a hierarchical structure (Fig. 2f, g). By further reducing the diameter of the NRs to 7.4 nm (Fig. 2i, sample named NRs-DDDAm-3) the length of the superlattices could be increased to ≈1500 µm, with a width of ≈1.4 µm (AR = 1070) (Fig. 2l). Under the optical microscope (Fig. 2i) also these 1D superlattices had a “wavy”configuration, and exhibited a multi-scale hierarchical structure under SEM and TEM (Fig. 2j, k).

We also synthesized thin NPLs by replacing DDDAm with oleylamine (OAm) in the synthesis (Fig. 2m). The NPLs in this case had a length of 35 nm and a width of 26 nm (they were named “NPLs-OAm”, see Supplementary Fig. 6). The NPLs have a thickness of 4.6 nm, corresponding to 5-monolayer (5-ML) CsPbBr_3_ NPLs. The superlattices built from this sample had a length of ≈1000 µm and width of ≈0.8 µm (Fig. 2p), resulting in an AR of 1250. These results indicate that the length and aspect ratio of the 1D superlattices can be controlled by adjusting the aspect ratio of the NRs/NPLs (Supplementary Fig. 8).

The NRs/NPLs stack with each other both along their thickness direction (along the long axis of 1D superlattice) and their lateral direction (along the short axis of the 1D superlattice), as shown in Supplementary Fig. 9. We have labeled the *x*-*z* directions in Fig. 2c–o to show this relation. In detail, the length of the 1D superlattices (*L*_SL_) follows the formula *L*_SL_ = *N*⋅*d* (where *N* is the number of NRs/NPLs along the long axis, and *d* is the sum of thickness of a single NPL and the length of a partial interdigitated ligand layer). Correspondingly, the width of the 1D superlattices (*W*_SL_) follows the formula *W*_SL_ = *M*⋅*l* (where *M* is the number of NRs/NPLs along the short axis, and *l* is the lateral size of a single NR/NPL).

For the vertical direction (along the long axis of the 1D superlattices), the inter-nanoparticle spacing (*d*_*z*_) can be directly calculated based on SAXS data (Supplementary Fig. 10), by the formula *d*_*z*_ =(2π/*q*)-*d*_0_, where 2π/*q* is the stacking distance measured by SAXS and *d*_0_ is the thickness of the NPLs (or NRs). For example, the alkyl chain lengths (*L*) of DDDAm and OAm are ≈1.5 nm and ≈1.7 nm, respectively, and the lateral spacing (*d*_*x*_) between adjacent NRs/NPLs is ≈2.3 nm and ≈3.0 nm, respectively (Supplementary Fig. 10c, d). *d*_z_ is generally smaller than twice the length of the ligands (2 *L*), due to partial interdigitation of ligands. The interdigitation factor Ƞ can be calculated as (*d*_*z*_/(2 *L)*, and is usually in the 77%-80% range. Overall, the inter-nanoparticle space between adjacent NRs/NPLs increases following the increase in alkyl chain lengths of the ligands^35,36^. The NPLs are densely stacked along the vertical direction of the 1D superlattices, but relatively loosely packed with neighboring NPLs along the lateral direction. This discrepancy arises because the lateral size distribution of the NPLs is less uniform than their thickness distribution. Ideally, the NPLs exhibit no variation in thickness. Therefore, dense packing is only achieved in the thickness direction.

**Fig. 3:**
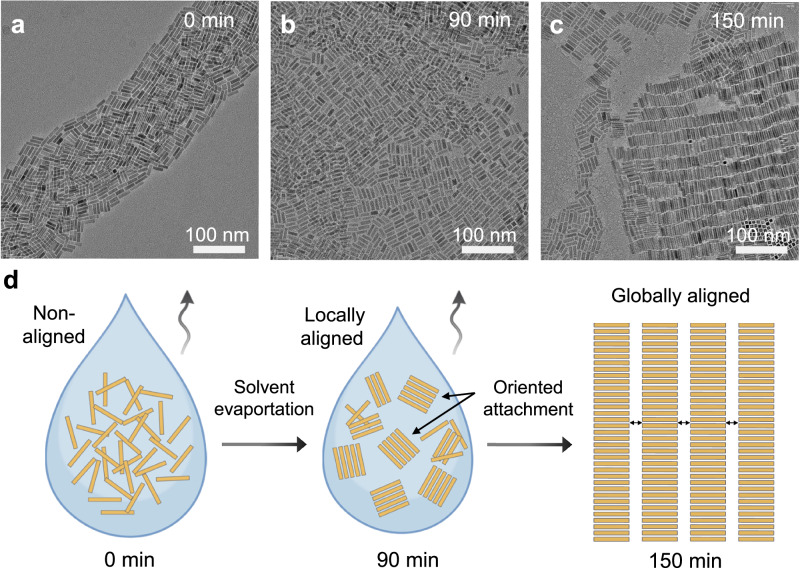
Monitoring the formation process of one-dimensional superlattices by cryo-TEM. **a**–**c** Cryo-TEM images during the process of solvent evaporation, for evaporation times of 0, 90, 150 min, respectively. **d** Sketches of the assembly process during the evaporation, from non-aligned to locally aligned and then to globally aligned particles.

To investigate the assembly process starting from the initial droplet to dried fibrillar-like assemblies, cryo-TEM analysis at different times was carried out (Fig. 3). In detail, three copper net grids were placed on the substrate, and the NRs toluene solution (10 mg mL^−1^) was drop-cast on each grid. Each grid was then rapidly plunge-frozen at different times (0, 90, 150 min) using liquid ethane. As shown in Fig. 3a, the cryo-TEM images of the NRs sample at 0 min did not evidence any clear alignment globally, as the NRs mainly formed disordered clusters. At 90 min, the NRs started to organize into locally aligned zones (Fig. 3b). At 150 min, globally aligned NRs were observed, in line with the corresponding 1D superlattices observed under the optical microscope (Fig. 2a). Given that NPLs and NRs share a similar anisotropic nature, and considering that NPLs ultimately form even longer superlattices, we deduce that the NPLs follow a self-assembly process that is fundamentally similar to that of the NRs.

### Polarized emission from the 1D superlattices

**Fig. 4:**
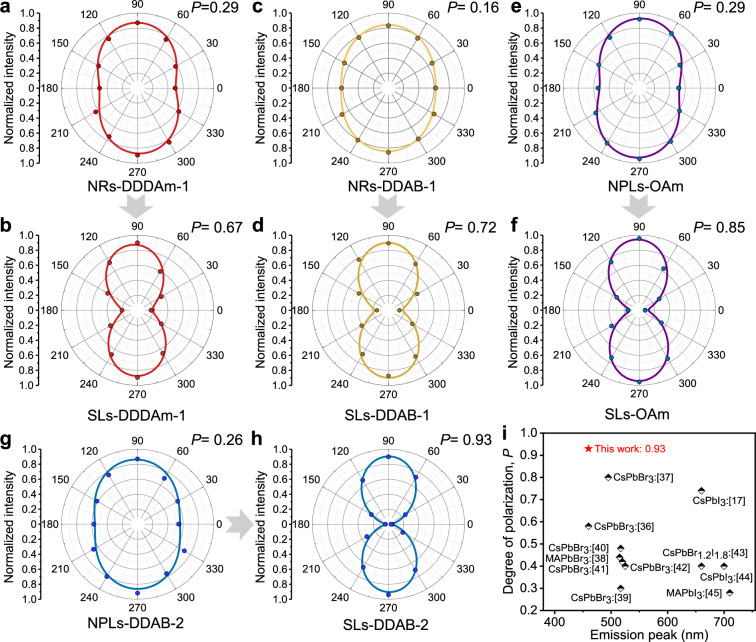
Polarized emission from 1D superlattices. Polar plot of the photoluminescence intensity for (**a**) randomly assembled NRs-DDDAm-1 film and (**b**) 1D NRs-DDDAm-1 superlattices; (**c**) randomly assembled NRs-DDAB-1 film and (**d**) 1D NRs-DDAB-1 superlattices; (**e**) randomly assembled NPLs-OAm film and (**f**) 1D NPLs-OAm superlattices; (**g**) randomly assembled NPLs-DDAB-2 film and (**h**) 1D NPLs-DDAB-2 superlattices. **i** Degree of polarization and emission peak position from published works on anisotropic perovskite nanocrystals and our 1D NPLs-DDAB-2 superlattices. The red, yellow, purple, and cyan curves correspond to NRs-DDDAm-1/SLs-DDDAm-1, NRs-DDAB-1/SLs-DDAB-1, NPLs-OAm/SLs-OAm, and NPLs-DDAB-2/SLs-DDAB-2, respectively. Source data are provided as a Source Data file.

Due to their anisotropic shape, individual CsPbBr_3_ NRs or NPLs are known to show polarized emission^36,37^. A random assembly of NRs or NPLs on the other hand can partially average out this polarization. The ordered 1D superlattices of this work are instead built from oriented NRs or NPLs and are thus expected to preserve the polarized emission. We prepared a randomly assembled film sample (Fig. 4a) and a 1D superlattice sample (Fig. 4b) using NRs-DDDAm-1 as building blocks. The random film sample had a degree of polarization equal to 0.29, where *P* = (*I*_max_–*I*_min_)/ (*I*_max_ + *I*_min_), consistent with that of previous reports^36,37^. In contrast, the 1D superlattices sample exhibited a *P* of 0.67, more than twice higher than the randomly assembled films. The other 1D superlattice samples also had high *P* (Fig. 4c–h). The 1D NPLs-DDAB-2 superlattices sample exhibited a near unity *P* (0.93), the highest value for anisotropic perovskite nanocrystals to date (Fig. 4i)^17,36–45^. To analyze the polarized emission direction of the samples, we employed a simplified polarization system based on a RX50M upright microscope in this study. This system consists of a polarizer and an analyzer, where the detection angle can be tuned by rotating the rotatable analyzer (0–360°). As shown in Supplementary Fig. 12, the fluorescence intensity of the sample exhibits a trend of “increase-decrease-increase-decrease” by rotating the analyzer in steps of 90°. In particular, the weakest and strongest intensity appeared at 45° and 135°, respectively, which indicates that the polarization direction is perpendicular to the long axis of the 1D superlattices, or parallel to the long axis of the NRs/NPLs. This indicates that the polarized emission stems from the building blocks of the 1D superlattices^17,46,47^.

### Chiral optical properties of 1D superlattices

**Fig. 5:**
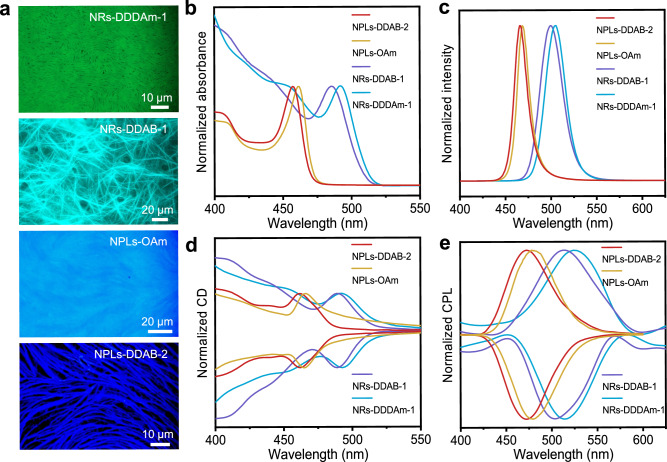
Chiral optical response of the 1D superlattices. **a** Optical microscope (OM) of 1D superlattices under the excitation of 365 nm light. The building blocks were NRs-DDDAm-1, NRs-DDAB-1, NPLs-OAm and NPLs-DDAB-2 from top to bottom, respectively. **b** UV-vis absorption and (**c**) photoluminescence spectra of NRs-DDDAm-1 (cyan), NRs-DDAB-1 (purple), NPLs-OAm (yellow) and NPLs-DDAB-2 (red) superlattices, respectively. **d** Circular dichroism and (**e**) circularly polarized photoluminescence spectra of NRs-DDDAm-1 (cyan), NRs-DDAB-1 (purple), NPLs-OAm (yellow) and NPLs-DDAB-2 (red) superlattices, respectively. All the intensities plotted in panels b-e were normalized. Source data are provided as a Source Data file.

As seen in Fig. 5a, the emission color of the superlattices (from blue to green) was strongly correlated with the thickness of the NRs/NPLs, and the absorption and photoluminescence spectra (Fig. 5b, c) exhibited gradually blue-shifted peaks with decreasing NRs/NPLs thickness^48^. We also collected circular dichroism (CD) and circularly polarized luminescence (CPL) spectra. As seen in Fig. 5d, the CD peaks of the NRs-DDDAm-1 superlattices were at 492 and 455 nm, respectively, in a mirror relation with the absorption peaks (Fig. 5b). The CD spectra of the other superlattices also matched well with their absorption spectra. These rotated stacked NPLs in 1D superlattices likely exhibit distorted lattice and contribute to the chirality, similar to non-chiral ligands involved chiral CsPbBr_3_ nanowires and nanoclusters^29,49^. It is worth noting that the randomly assembled NPLs exhibit non-detectable CD response, while the 1D NPLs-OAm superlattices show a reasonable CD signal (*g*_abs_ is on the order of 10^−3^) (Supplementary Fig. 13). This indicates that the ordered macroscopic pattern plays a key role in preserving or amplifying the intrinsic chirality.

To evaluate the thickness dependent CD spectra, we used different concentrated NPLs-OAm solutions (5-20 mg mL^−1^) to prepare the SLs-OAm film with different thicknesses. As shown in Supplementary Fig. 16, the average thickness is determined by cross-sectional SEM. It decreases from 328 to 235 and to 145 nm, following the decrease in concentration from 20 to 10 and to 5 mg mL^−1^. The absorption spectra and the CD spectra were collected on all the samples and the corresponding dissymmetry factors (*g*_abs_) were calculated. The results indicate that the dissymmetry factor (*g*_abs_) is thickness dependent: a thicker SLs film has a larger value of *g*_abs_ (Supplementary Fig. 16i). The CD spectra were collected using the so-called four-scan method (the sample is scanned at four different orientations, (1) *θ* = 0°, *β* = 0° (2) *θ* = 90°, *β* = 0° (3) *θ* = 0°, *β* = 180° and (4) *θ* = 90°, *β* = 180° and averages are made over the four scans) (Supplementary Fig. 17). This allowed us to subtract the contribution of linear dichroism to the CD spectra, hence retaining only the “true CD” signal.

The optical chirality was also evident in the circularly polarized emission (Fig. 5e). The NPLs-DDAB-2 superlattices sample has a dissymmetry factor (*g*_lum_) of −0.11 (Supplementary Fig. 20)^29,32,50,51^. The dissymmetry factor (*g*_lum_) is ~10^−2^, which is too small to show detectable PL intensity difference (≈1%) in the angular resolved PL. Both the CsPbBr_3_ NRs and NPLs can assemble into ordered 1D superlattices. As shown in Supplementary Table 1, the NPLs-based 1D superlattices exhibit larger aspect ratio, higher polarized emission and larger dissymmetry factor *g*_lum_ than the NRs superlattices, possibly due to the stronger anisotropic shape of the NPLs. Only the electronic ground state contributes to CD measurement, while the CPL process encompasses a broader range of excited transitions relevant for luminescence, which can lead to higher dissymmetry factors due to the cumulative effects of multiple contributing transitions^31^.

### Chirality control by addition of small chiral molecules

**Fig. 6:**
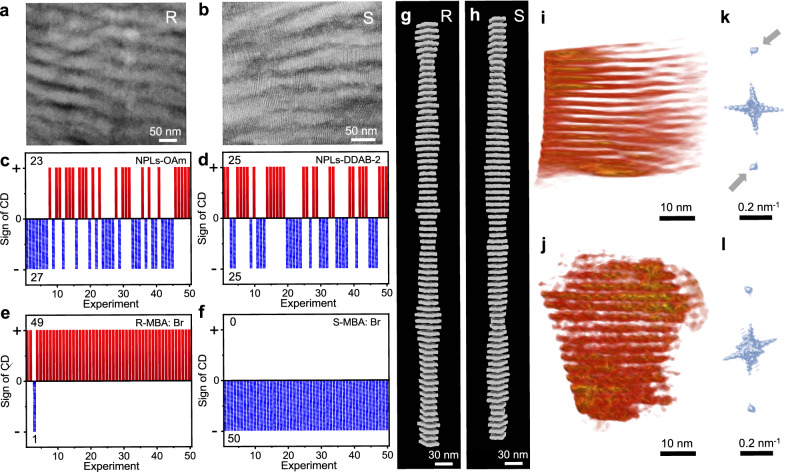
Chirality control of 1D superlattices by the addition of small chiral molecules. STEM image of (**a**) R-configuration ribbons and (**b**) S-configuration ribbons in the superlattices. Statistical counting of the CD signal for 50 batches of (**c**) NPLs-OAm superlattices and (**d**) 1D NPLs-DDAB-2 superlattices. Statistical counting of CD signal for 50 batches of (**e**) R-MBA:Br treated and (**f**) S-MBA:Br treated NPLs-OAm superlattices, respectively. The positive CD signal (red bar) was counted as +, and the negative signal (blue bar) was counted as -. The 3D model of (**g**) R-configuration ribbon and (**h**) S-configuration ribbon, respectively. The rotation angles of each NPL were calculated based on the width variation within the ribbons in STEM. **i**, **j** 3D reconstructed volume. The pseudocolor in (**i**, **j**) denotes the normalized contrast intensity of the 3D reconstructed volume. Bright red corresponds to regions with the maximum contrast, while dark brown indicates the minimum contrast areas. **k**, **l** 3D fast Fourier transform (FFT) analysis on the reconstructions of the stacked NPLs. Source data are provided as a Source Data file.

As discussed in the previous section, these 1D superlattices exhibited chiral optical response already with no involvement of chiral ligands or templates^52,53^. We deduce that the observed chirality is attributed to the configuration of the ribbons. As shown in Fig. 6a, b, the width was variable in one ribbon, although the NPLs in the starting solution were highly homogeneous in shape (Supplementary Fig. 21). This width variation suggests that each NPL is slightly rotated (twisted) along the helical axis in the ribbon compared to its neighbors (Fig. 6a, b)^28,54,55^. The rotation angle (*θ*) for a given NPL was calculated by the equation: cos*θ* = *L*_m_/*L*_0_, where *L*_m_ is the apparent width within the ribbon and *L*_0_ is the actual lateral size of the NPL, taken as the maximum value of *L*_m_ observed. 3D models of the helical ribbons were then constructed based on these calculated rotation angles (Fig. 6g, h). Regarding the previously reported CdSe NPLs helices^28^, these helices are well dispersed and do not interact with each other. Consequently, left- and right-handed helices are present in nearly equal amounts, resulting in a racemic system that exhibits no chiroptical response. In contrast, in our work, the densely aligned superlattices enable an autocatalytic effect^56^: an initial small fluctuation in one handedness promotes the formation of the same handedness, ultimately leading to macroscopic chiral symmetry breaking.

Statistical analysis of 50 batches of 1D NPLs-OAm superlattices revealed a near 1:1 frequency ratio between the positive and negative CD signals, confirming a near-stochastic chirality direction (Fig. 6c)^31,56^. This random chirality direction was also observed in the other 1D superlattices (Fig. 6d and Supplementary Fig. 22). We then introduced small chiral molecules (R/S-methylbenzylammonium bromide (R/S-MBA:Br)) during the assembly process of the NPLs. In detail, 10 mg of R-MBA: Br or S-MBA:Br dissolved in toluene were added into 3 mL of the as-obtained NPLs-OAm obtained, respectively, and the mixture was then vigorously stirred for 1 min. These NPLs were separated by adding ethyl acetate followed by centrifugation. After that, the treated NPLs were dispersed in toluene for the assembly experiments. As shown in Fig. 6c, after this chiral ligand (R-MBA:Br) treatment, 50 batches of NPLs-OAm superlattices exhibited 98% (49/50) positive signals. Similarly, in the S-MBA:Br treated case, 50 batches of NPLs-OAm superlattices exhibited 100% (50/50) negative signal (Fig. 6e, f). Such near unity chirality selection was also seen in the 1D NRs-DDDAm-1 superlattices (Supplementary Fig. 23). We attribute the high enantiomer-selectivity to an initial chiral distortion of the NPL lattice induced by the chiral ligands^57,58^, which is subsequently amplified during the assembly process via an autocatalytic effect^31,59^.

To directly investigate the stacking of NPLs in the superlattices (NPLs-OAm based 1D superlattice), we employed 3D reconstruction based on scanning TEM (Fig. 6i, j). As shown in Supplementary Fig. 24, four orthoslices of stacked NPLs were collected, and used to reconstruct a 3D image (Supplementary Fig. 24a). The 3D structure is shown in Supplementary Movie 1. 3D FFT analysis of the stacked NPLs was carried out, as shown in Fig. 6k, l. The discrete dots (highlighted by the gray arrows) indicate a distorted lamellar pattern between the stacked NPLs, in analogy with refs. ^60,61^. These results support our hypothesis that the NPLs are stacked along their long axis with different rotation angles relative to each other. The driving force for this stacking geometry probably stems from the ligand-induced mechanical stress on CsPbBr_3_ NPLs, which has been studied in detail for stacked CdSe NPLs^28^. Essentially, in an isolated NPL, the ligands on surface are free to rotate. However, after stacking with other NPLs, the ligands lock each other and such rotation freedom is limited^62,63^. This translates into a reduction of the entropy and an increase of the Gibbs energy in the stacked system, generating extra mechanical stress that twists this NPL to partially recover the rotation freedom of the ligands^28^.

### CP-ASE of 1D NPL superlattices

**Fig. 7:**
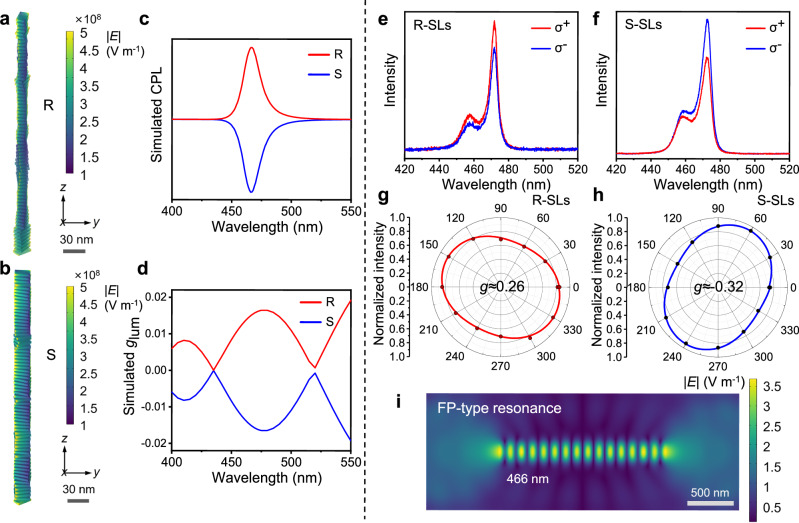
Simulated CPL response and circularly polarized ASE of the 1D superlattices. **a**, **b** Electric field passing through (a) right-handed chiral superlattice and (**b**) left-handed chiral superlattice. The color bar represents the electric field strength |*E*|, with the unit of V m^−1^. **c** Calculated CPL response and (**d**) *g*_lum_ curve by COMSOL Wave Optics Module. The red curves represent the R-SLs, while the blue curves represent the S-SLs. **e**, **f** Right and left circularly polarized amplified spontaneous emission (ASE) outputs from the 1D superlattices with different chiral configurations (R-/S-), respectively. The red line represents the right-handed circularly polarized ASE component (σ⁺), and the blue line represents the left-handed circularly polarized ASE component (σ⁻). **g**, **h** Polarization angle-dependent ASE intensities from the R/S-1D superlattices, respectively. The radial axis represents the relative ASE intensity, and the angular axis represents the polarization angle. The red curves represent the R-SLs, while the blue curves represent the S-SLs. **i** Simulated field intensity distribution (*λ* = 466 nm, *n* = 2.6) in a superlattice with *L* = 1.7 μm. The simulated field intensity distribution indicates that the superlattice can function as a FP optical cavity for realizing laser oscillations. The color bar represents the electric field strength |*E*|, with the unit of V m^−1^. Source data are provided as a Source Data file.

Electromagnetic simulations were performed on left-handed (LH) and right-handed (RH) one-dimensional superlattices using the COMSOL Wave Optics Module (Fig. 7a–d). The experimentally measured PL spectrum was used as the incident light source, which, after modulation by the helical structure (Fig. 7a, b), produced the CPL spectrum (Fig. 7c). The results show that the simulated CPL characteristic peaks of the two chiral films are in strong agreement with the experimental measurements (Fig. 5e), and the simulated *g*-factors were of the order of 10^−2^ (Fig. 7d).

Under high-power laser pumping (35 µJ cm^−2^) a sharp and intense emission signal was observed, characteristic of amplified spontaneous emission (ASE), with a full width at half maximum (FWHM) of 5-7 nm (Fig. 7e, f). On the other hand, the random assembled NPLs-OAm films did not show ASE under the same pumping densities (Supplementary Fig. 26). We conclude that the ordered patterns of 1D superlattices work as optical microcavities that induce ASE^64^. Optical simulations were performed to demonstrate the optical microcavities effect of 1D superlattices (Fig. 7i). The results indicate that the 1D superlattices can naturally form effective Fabry–Pérot (FP) optical cavities, providing feedback for laser oscillation. This proves that the system possesses a favorable optical cavity effect.

The ASE was also circularly polarized, an effect that was ascribed to the helical configuration of the 1D superlattices. The dissymmetry factor (*g*) of the circularly polarized ASE was 0.26 (R) and −0.32 (S), respectively, 3-4 times higher than the *g*_lum_ of circularly polarized luminescence, due to enhanced coupling between the light and the chiral structures (Fig. 7g, h)^65^. In the microcavity, photons are confined and oscillate repeatedly. During each round trip, the right-handed circularly polarized (RCP) component experiences greater gain than the left-handed circularly polarized (LCP) component. After hundreds or thousands of round trips of amplification, the small inherent differences are continuously accumulated and amplified, leading to a rapid increase in the output *g*_ASE_ value, which far exceeds the intrinsic *g*_lum_ value. In the absence of chiral ligands or other templates, both the CPL and ASE are attributed to the twisted stacking pattern of the NPLs within 1D superlattices^64^.

This work demonstrates the fabrication, elucidation of hierarchical structure, and polarized optical properties of 1D superlattices of CsPbBr_3_ nanorod/nanoplatelets. Due to their anisotropic geometry, the superlattices show polarized emission with a near unity *P* value, 4 times larger than the films from the corresponding randomly assembled CsPbBr_3_ nanorod/nanoplatelets. Also, the ribbons in the 1D superlattices have a helical configuration, resulting in strong circular dichroism and circularly polarized emission. With addition of small chiral ligands, the CD signal (negative or positive) of the superlattices can be well controlled, with an enantiomeric excess (ee) value over 98%. The maximum dissymmetry factor (*g*_lum_) of the superlattice is −0.11. These 1D superlattices act as optical microcavities capable of inducing circular polarized amplified spontaneous emission, with an amplified *g*_lum_ factor of −0.32. This work extends the concept of 1D perovskite nanocrystal superlattices and reveals their promising properties of polarized emission, circularly polarized emission and circularly polarized amplified spontaneous emission.

## Methods

### Materials

Lead acetate trihydrate [(Pb(Ac)_2_·3H_2_O), 99.99%], cesium carbonate (Cs_2_CO_3_, 99%), benzoyl bromide (C_6_H_5_COBr, 97%), ethyl acetate (98.8%), toluene (anhydrous, 99.5%), toluene-d_8_ (99 at% D), didodecyldimethylammonium bromide (DDAB, 98%), oleylamine (OAm, 70%), (R)-(+)-methylbenzylamine (R-MBA, 99%), (s)-(-)-methylbenzylamine (S-MBA, 99%), (R)-(+)-methylbenzylammonium bromide (R-MBA:Br, 99%), (S)-(-)-methylbenzylammonium bromide (S-MBA:Br, 99%), 1-octadecene (70%) and oleic acid (OA, 70%) were purchased from Sigma-Aldrich. Didodecylamine (DDDAm, 97%) was purchased from TCI. Oleylamine (70%), oleic acid (70%) and 1-octadecene (70%) were degassed at 120 °C and stored in the glovebox before use. Toluene and ethyl acetate were purchased from General Reagents Co., Ltd. and were degassed to remove residual water before use. All the other chemicals were used without any further purifications.

### Synthesis of CsPbBr_3_ nanorods (NRs-DDDAm-1)

A total of 76 mg (0.2 mmol) lead (II) acetate trihydrate, 16 mg (0.05 mmol) cesium carbonate, and 10 mL of octadecene were mixed in a 25 mL flask. The reaction mixture was degassed for 5 min at room temperature then for 1 h at 115 °C. 1.5 mL degassed oleic acid and 1.25 mmol DDDAm dispersed in 1 mL of anhydrous toluene were rapidly injected into the mixture under nitrogen. The mixture was stirred at 115 °C until all the metal salts were dissolved. The temperature was then kept at 40 °C (For NRs-DDDAm-2 and NRs-DDDAm-3, the temperature was reduced to 30 °C and 25 °C, respectively.). A total of 50 μL benzoyl bromide diluted in 500 μL of ODE was then injected into the mixture. The reaction was quenched after 15 s by immersion in a water bath (note: for NRs-DDDAm-3, an ice-water bath was used instead). Subsequently, 30 mL of an ethyl acetate and toluene mixture (with a ratio of 6:1) were added to the crude solution to precipitate the nanorods, followed by centrifugation at 6000 × *g* for 10 min. Finally, the supernatant was discarded, and the nanorods precipitates were redispersed in toluene.

### Synthesis of CsPbBr_3_ nanoplatelets (NPLs-OAm)

A total of 76 mg (0.2 mmol) lead (II) acetate trihydrate, 16 mg (0.05 mmol) cesium carbonate, and 10 mL of octadecene were mixed in a 25 mL flask. The reaction mixture was degassed for 5 min at room temperature then for 1 h at 115 °C. 1.5 mL of degassed oleic acid and 1.25 mmol oleylamine dispersed in 1 mL anhydrous toluene were rapidly injected into the mixture under nitrogen. The mixture was stirred at 115 °C until all the metal salts were dissolved. The temperature was then lowered to 30 °C. 50 μL of benzoyl bromide diluted in 500 μL of ODE was then injected into the mixture. The reaction was quenched after 1 min by immersion in a water bath. Subsequently, 30 mL ethyl acetate/toluene mixture (with a ratio of 6:1) were added to the crude solution to precipitate the nanoplatelets, followed by centrifugation at 7000 × *g* for 10 min. Finally, the supernatant was discarded, and the nanoplatelet precipitate was redispersed in toluene.

### Ligand exchange with DDAB

All ligands exchange protocols were performed under air. Briefly, the crude reaction solution (3 mL) containing the CsPbBr_3_ nanorods or nanoplatelets was mixed with a DDAB-toluene solution (2 mL, 25 mM) and then the mixture was vigorously stirred for 1 min. After that, the sample was precipitated by addition of 15 mL ethyl acetate, followed by centrifugation at 6000 × *g* for 10 min. The nanorods or nanoplatelets precipitate was redispersed in a diluted DDAB-toluene solution (1 mL, 2 mM). The sample was then precipitated again following the previous protocols. The ligand exchange procedure was repeated three times to enhance the stability and emission intensity of the sample. However, excess exposure time of the nanorods or nanoplatelets to DDAB was found to cause their degradation.

### Ligand exchange with R/S-MBA:Br

A solution of R/S-MBA:Br (10 mg) in toluene (2 mL) was prepared in a sample vial. The mixture was slightly heated until complete dissolution of the salt. Briefly, the R/S-MBA:Br-toluene mixture was added to crude solution of CsPbBr_3_ nanorods or nanoplatelets (3 mL) and the resulting mixture was vigorously stirred for 1 min. After that, the sample was precipitated by addition of 20 mL ethyl acetate, followed by centrifugation at 6000 ×*g* for 10 min. The precipitate was redispersed in toluene for further assembly experiments. An excess of R/S-MBA:Br was found to cause degradation of the nanorods or nanoplatelets.

### Assembly experiment

The glass slides were treated with perfluorodecyltriethoxysilane to enhance their interaction with the nanorods or nanoplatelets solution. After that, a treated glass slide was put inside the container of a Petri dish (Supplementary Fig. 4). 30 μL of the stock solution was dropped onto the glass slide by means of a pipette. The container was then covered by the lid and then wrapped in tin foil to shield it from light and wind. With this arrangement, the solvent evaporation slowly over several hours. After that, the glass slides were recovered and used for other characterizations. If the Petri dish container was not covered by the lid, the evaporation became very fast, and the so-called ‘randomly assembled film’ was formed in a few minutes.

### UV-Vis absorption measurements

The absorption spectra of the nanorods or nanoplatelets solutions were measured on the SHIMADZU UV-2600i UV-Vis spectrometer. The absorption spectra of the superlattices were measured on a Chirascan V100 spectrophotometer.

### PL and PLQY measurements

An Edinburgh FLS1000 spectrofluorometer was employed for recording the PL of the nanorods or nanoplatelets solution and the superlattices. PLQY measurements were performed using an integrating sphere attached to the Edinburgh FLS1000 spectrofluorometer, following the absolute method.

### CD and CPL measurements

The CD spectra and CPL spectra were recorded on a Chirascan V100 and JASCO CPL-300 spectrophotometers, respectively. The CD spectra were collected using the four-scan method to avoid linear dichroism and linear birefringence (LDLB) effects. The prepared sample was fixed on the test stand, and the CD signals at 0°, 90°, −0°, and −90° were measured. The average value of the four measurements was calculated as ‘pure’ CD signal of the sample. All data processing was performed using Origin 2021. All data are processed using the maximum-value normalization method. That is, for each spectral curve, the maximum signal intensity is set as 1, and the signal intensities of all other points on the curve are divided by this maximum value, thereby obtaining the normalized spectral data.

### Polarization emission characterizations

Polarization measurements were carried out on an Edinburgh FLS980, using linearly polarized light as the excitation light source.

### XRD measurements

XRD patterns were recorded on a Miniflex 600 benchtop X-ray diffractometer equipped with a 0.6 kW Cu *K*_a_ X-ray tube and a HyPix-MF 2D area detector, operating at 45 kV and 15 mA. For the measurements, the solution was deposited on a zero-background silicon wafer and the solvent was allowed to evaporate before recording the XRD patterns. The scanning angle ranged from 5° to 80° at a scan rate of 10° min^−1^.

### Amplified spontaneous emission measurements

The optically pumped lasing measurements were carried out on a far-field micro-photoluminescence measurement system equipped with a femtosecond pulsed laser (Spectra Physics), an optical microscope (Nikon), a charge-coupled device camera (Nikon), and a spectrophotometer (Princeton Instruments).

### SAXS measurements

The SAXS analysis was performed on a Bruker D8 Advanced X-ray Diffractometer, and the scanning 2*θ* angle ranged from 0.5° to 5° at a scan rate of 0.5° min^−1^.

### Optical microscopy

Optical microscopy images were collected on a SOPTOP RX50M microscope.

### Transmission electron microscopy

TEM measurements were performed on a FEI Talos F200s microscope operated at 200 kV. A carbon-coated copper mesh was first placed on the glass substrate, after which a drop of the nanorods or nanoplatelets-toluene suspension was deposited on it and then evaporated to form superlattices.

### Scanning electron microscopy

SEM measurements were carried out on a ZEISS Gemini 300 scanning electron microscope equipped with a SE2 secondary electronic detector operated at 3 kV. The glass slide was replaced by ITO for higher conductivity of sample.

### NMR measurements

The ^1^H-NMR tests were conducted on a Bruker-Avance NEO Ascend 500 M spectrometer. The sample was dissolved in toluene-d_8_ to form a high-concentrated and transparent solution.

### Time-resolved Cryo-TEM analysis

Cryo-TEM was performed on a Thermo Scientific Titan Krios G3i (USA) operated at 300 kV, equipped with automatic pick-up system of frozen sample. The carbon coated copper grid was placed on the glass substrate, after which a drop of the toluene suspension containing the NCs was deposited on it and the solvent was allowed to evaporate. Following evaporation times of 0, 90, and 150 min, the sample grid was rapidly plunge-frozen using liquid ethane cooled by liquid nitrogen. Plunge-freezing was performed using a Vitrobot Mark IV (Thermo Fisher Scientific) at room temperature.

### Electron tomography

Tomography series were acquired using an aberration corrected “cubed” Thermo Fisher Scientific Titan 60-300 electron microscope operated at 120 kV with a single-tilt tomography holder over a tilt range from −68° to + 72°, with tilt increments of 1°. The 3D reconstruction of the tilt series was performed using the SIRT algorithm. The volume rendering, digital slicing and images were processed using Avizo.

### Electromagnetic simulations

All numerical calculations were performed via finite-element simulations using the COMSOL software. Floquet periodic boundary conditions were applied in the transverse directions of the unit cell to define the wavevector, while a perfectly matched layer (PML) was adopted in the *z*-direction to simulate a reflection-free infinite domain. Simulations were carried out on structures modeled with left-handed and right-handed chirality. The incident light was directed vertically from the top port, and the left- and right-handed polarized signals modulated by the nanostructure were collected at the bottom port. Under linearly polarized light excitation, numerical simulations were performed for the left-handed and right-handed one-dimensional superlattices.

## Supplementary information

Supplementary Information

Description of Additional Supplementary Files

Supplementary Movie 1

Supplementary Data 1 (Unprocessed data)

Author Checklist

Transparent Peer Review file

## Source data

Source Data

## Data Availability

The data that support the findings of this study are available from the corresponding authors upon request. Unprocessed raw data are provided as Supplementary Data 1. Source data are provided with this paper.
