# Supplementary material for: One-dimensional CsPbBr3 superlattices with polarized and amplified spontaneous circularly polarized emissions
Source: Nat Commun. 2026 May 23;17:6768. doi: 10.1038/s41467-026-73513-2 (PMC13385850; doi:10.1038/s41467-026-73513-2)
Supplement: Supplementary file 1 — Supplementary Information [file 41467_2026_73513_MOESM1_ESM.pdf]

Supplementary information

One-dimensional $CsPbBr_3$ superlattices with polarized and amplified spontaneous circularly polarized emissions

Baowei Zhang[1, #], Kexin Chen[1, #], Zhengkun Xie[1], Kun Hu[2,3], Haiyun Dong[2,3], Yong Sheng Zhao[2,3], Liberato Manna[4, *], Siyu Lu[1, *]

[1] College of Chemistry, Pingyuan Laboratory, Zhengzhou University, Zhengzhou, 450000, China.
[2] Beijing National Laboratory for Molecular Sciences, Institute of Chemistry, Chinese Academy of Sciences, Beijing 100190, China
[3] School of Chemical Sciences, University of Chinese Academy of Sciences, Beijing 100049, China
[4] Nanochemistry, Istituto Italiano di Tecnologia, via Morego 30, 16163 Genova, Italy.

E-mail: **L. M. (*Liberato.Manna@iit.it*) and S. L. (*sylu2013@zzu.edu.cn*)**

The Supplementary Information includes:

Supplementary Fig. 1-26
Supplementary Table 1
Supplementary References

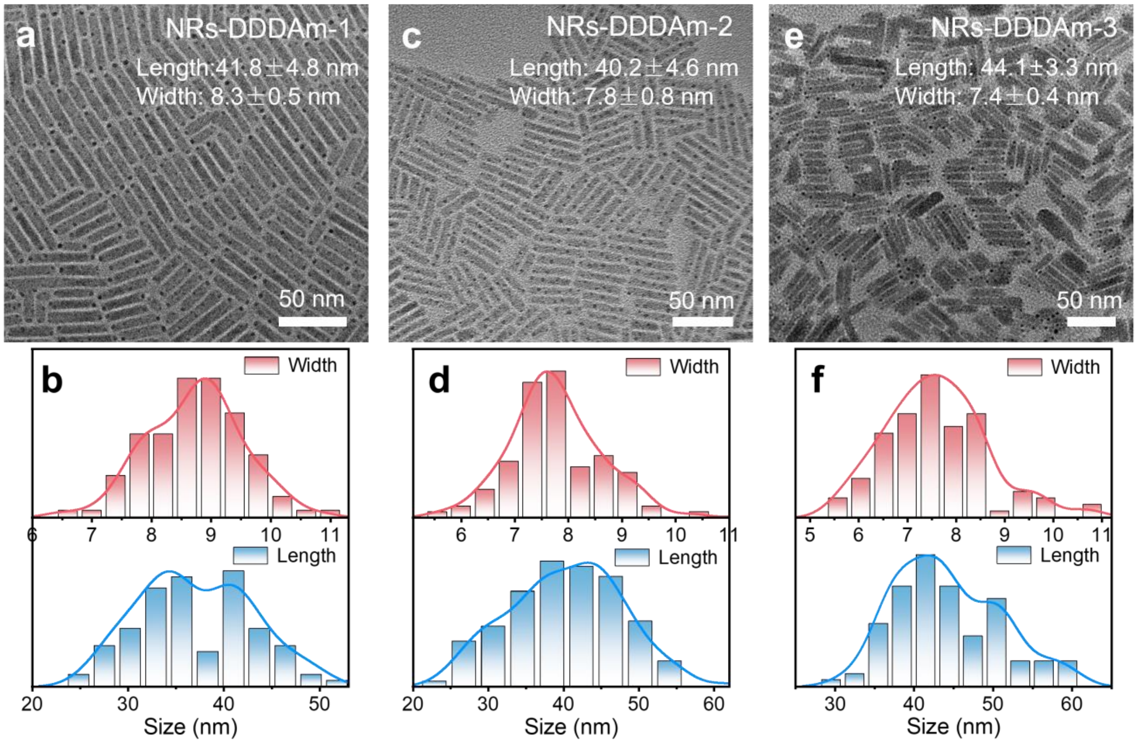

Supplementary Fig. 1 TEM images and size distributions of the nanorods synthesized using DDDAm at different temperatures. The nanorods with a diameter of 8.3 nm were named as NRs-DDDAm-1 (a-b, *d* = 8.3 nm), NRs-DDDAm-2 (c-d, *d* = 7.8 nm) and NRs-DDDAm-3 (e-f, *d* = 7.4 nm), respectively. Histograms of width (red) and length (blue) were derived from *n* = 100 individual nanorods for each sample (b, d, f). Source data are provided as a Source Data file.

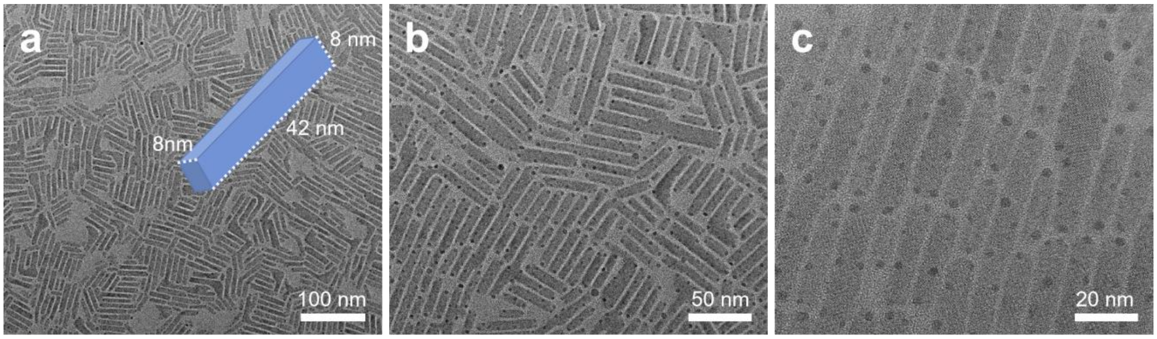

Supplementary Fig. 2 TEM characterization of the sample synthesized using DDDAm with concentrations of (a) 1 mg mL^{-1}, (b) 0.5 mg mL^{-1} and (c) 0.1 mg mL^{-1}, respectively. The images generally point to a nanorod (NR) shape. The geometrical parameters for this sample are: diameter of ≈8 nm and length of ≈42 nm (inset of panel (a)).

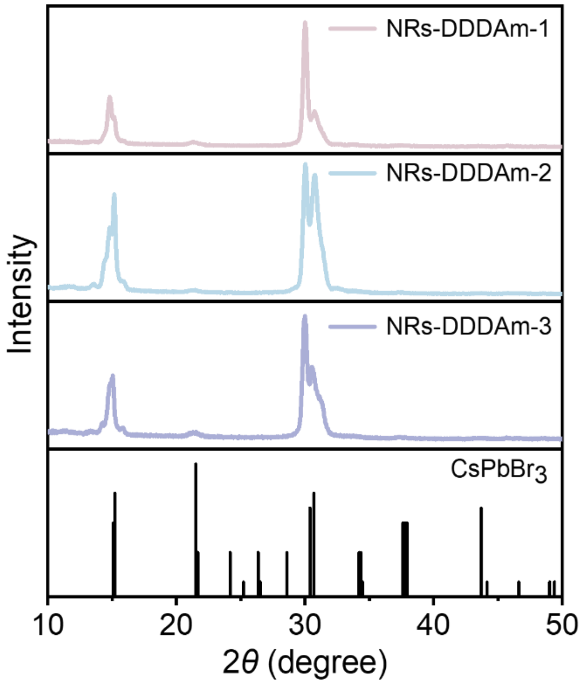

Supplementary Fig. 3 X-ray diffraction patterns of the NRs-DDDAm-1, NRs-DDDAm-2, and NRs-DDDAm-3 samples, respectively. The dark vertical lines represent the orthorhombic $CsPbBr_3$ phase (ICSD, #97851). The multi-peak features at 15° and 30° in the XRD patterns are the fringes due to the superlattices structure[1]. The curves in pink, blue, and purple represent NRs-DDDAm-1, NRs-DDDAm-2, and NRs-DDDAm-3, respectively. Source data are provided as a Source Data file.

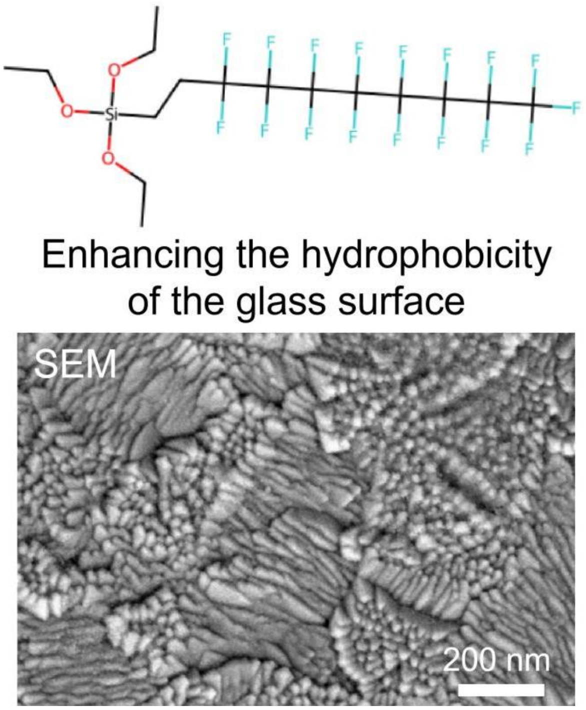

Supplementary Fig. 4 Molecular structure of perfluorodecyltriethoxysilane and SEM image of the modified glass substrate.

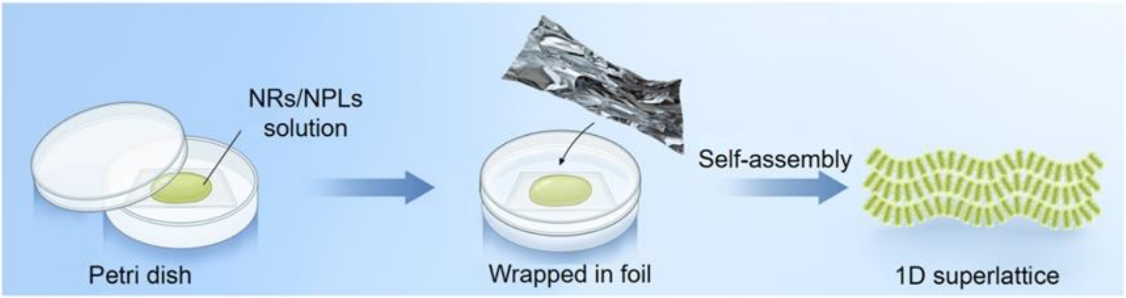

Supplementary Fig. 5 Sketch of the setup for the assembly experiment. The treated glass slide is placed in a Petri dish. 30 μL of the NCs solution (20 mg mL^{-1}) is dropped onto the glass slide. The lid of the Petri dish is used to cover the glass slide, and the whole setup is wrapped in aluminum foil[2]. This schematic diagram was drawn by Yao Tong using the open-source graphics editing software GIMP.

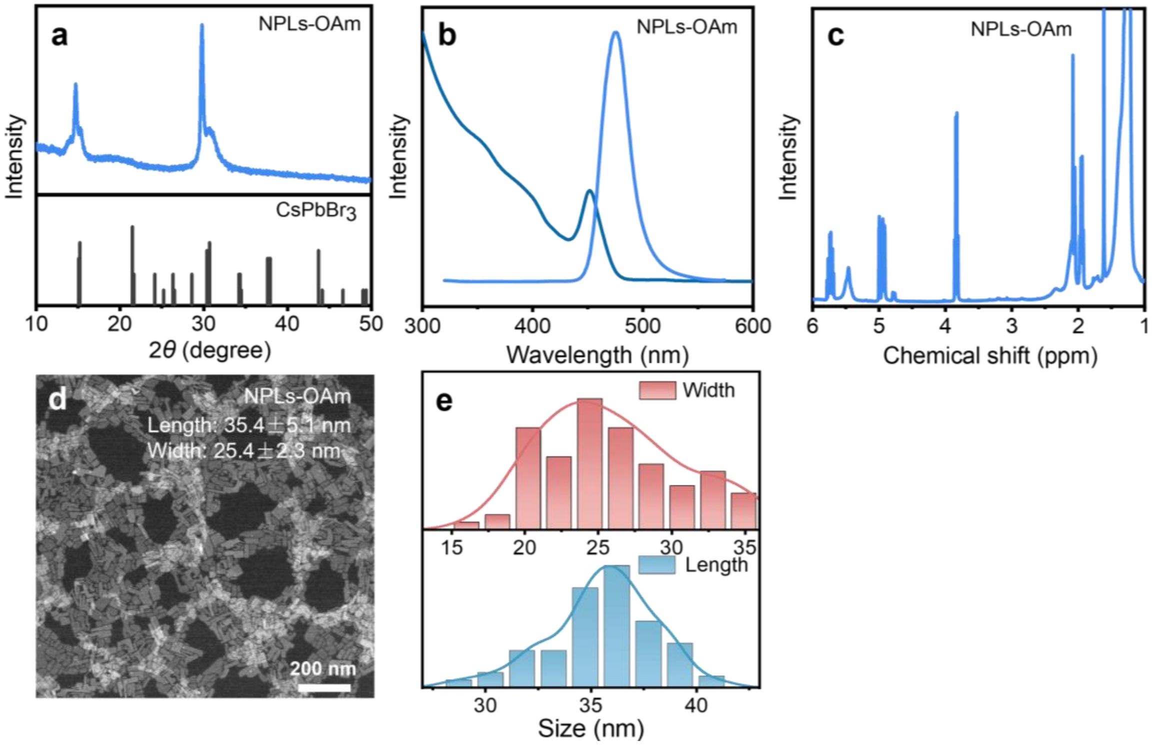

Supplementary Fig. 6 Primary characterizations of the nanoplatelets synthesized using oleylamine (NPLs-OAm). (a) X-ray diffraction pattern, (b) absorption and photoluminescence, (c) solution ^{1}H-NMR, (d) TEM and (e) size distribution of the NPLs-OAm. Histograms of width (red) and length (blue) were derived from n = 100 individual NPLs for each sample. Source data are provided as a Source Data file.

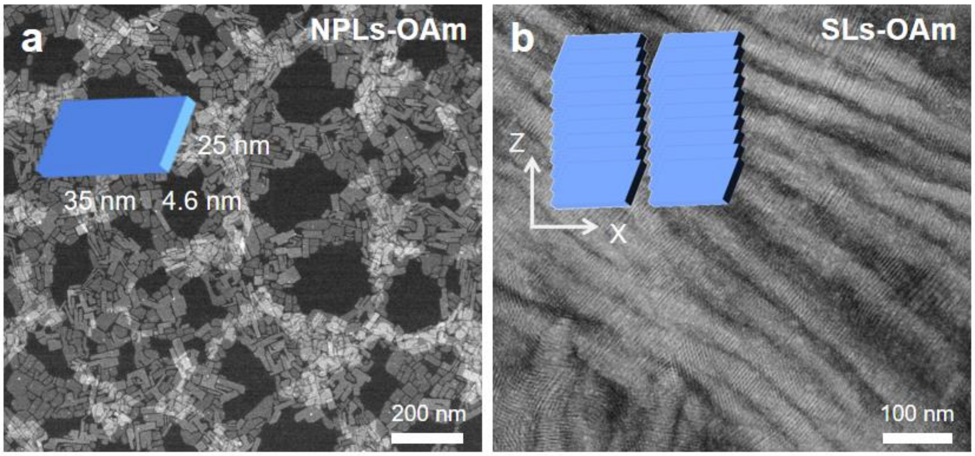

Supplementary Fig. 7 TEM images of (a) Edge-up stacked and (b) horizontally oriented NPLs-OAm. These indicate a NPL shape for the OAm sample with a thickness of ≈4.6 nm and lateral length of ≈25-35 nm.

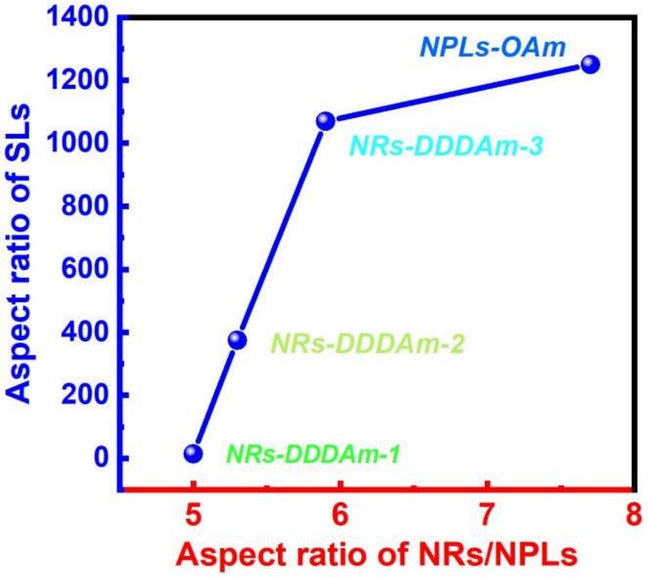

Supplementary Fig. 8 The relationship between the aspect ratio of superlattices (SLs) and the aspect ratio of nanoplatelets (NPLs) and nanorods (NRs).

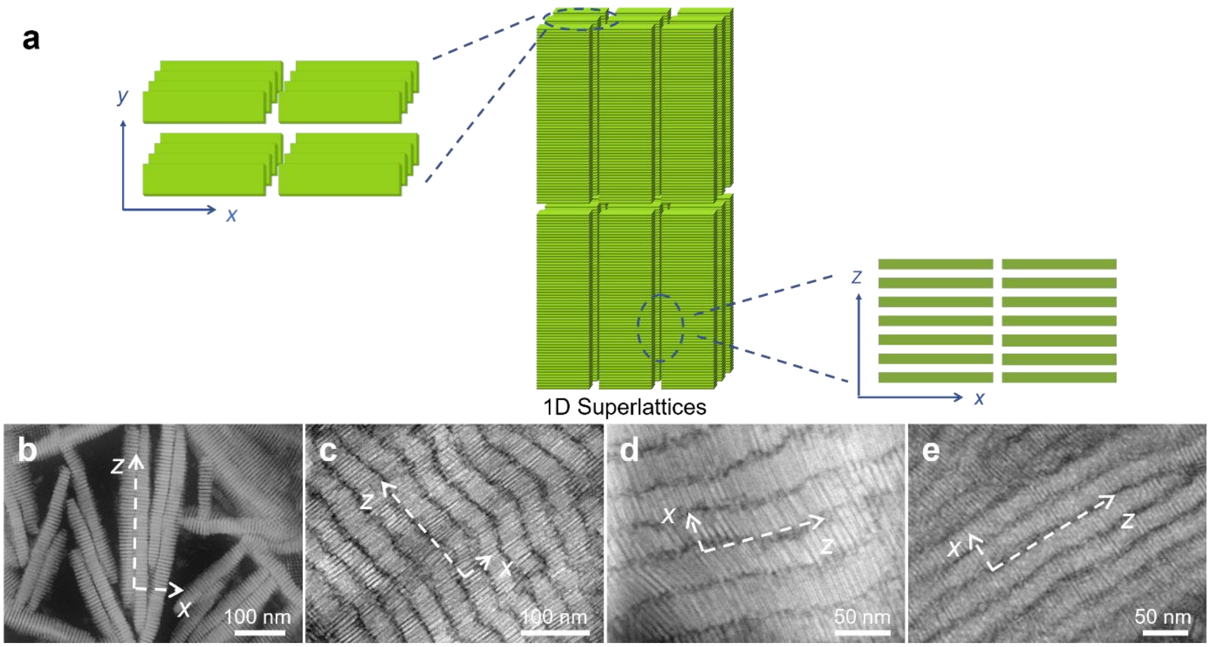

Supplementary Fig. 9 (a) Scheme of the stacking directions of NRs/NPLs in 1D superlattices; **(**b-e) STEM characterization of 1D NRs/NPLs superlattices.

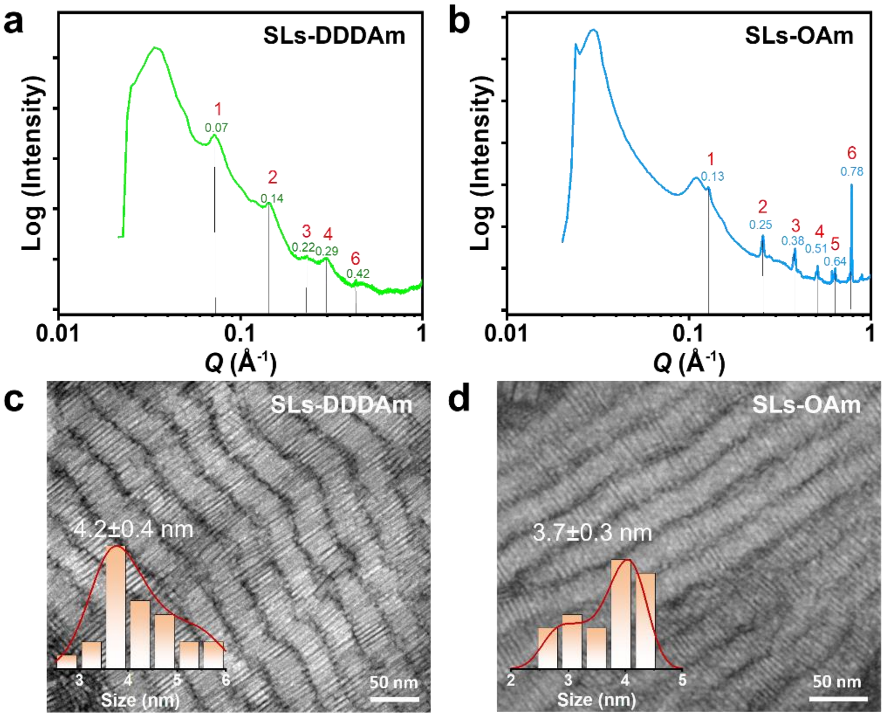

Supplementary Fig. 10 (a-b) SAXS data of (a) SLs-DDDAm and (b) SLs-OAm. (c-d) TEM images and size histograms of (c) SLs-DDDAm and (d) SLs-OAm, showing average lateral spacing of 4.2 $\pm$ 0.4 nm and 3.7 $\pm$ 0.3 nm, respectively. Histograms of lateral spacing were derived

from n =25 individual NRs/NPLs for each sample. The curves in green and blue represent SLs-DDDAm and SLs-OAm, respectively. Source data are provided as a Source Data file.

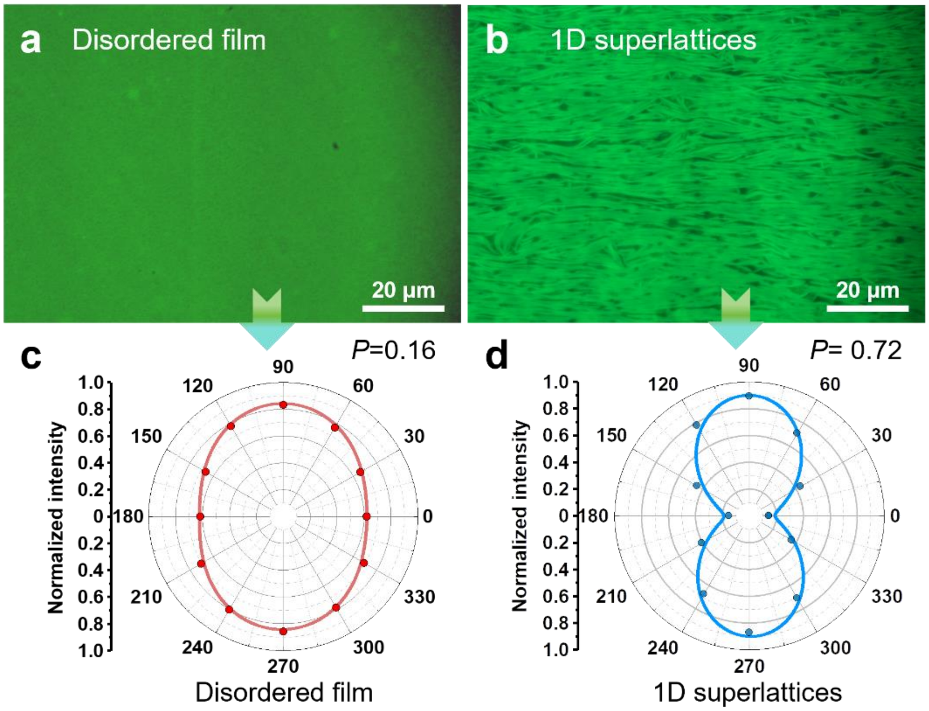

Supplementary Fig. 11 (a-b) Optical microscopy images of (a) a disordered NRs-DDDAm-1 film prepared by random evaporation assembly and (b) a 1D NRs-DDDAm-1 superlattices film prepared by confined evaporation; (c-d) Polar plots of the normalized intensity of the photoluminescence emission as a function of detection angle for (c) disordered NRs-DDDAm-1 film and (d) 1D NRs-DDDAm-1 superlattices film. Source data are provided as a Source Data file.

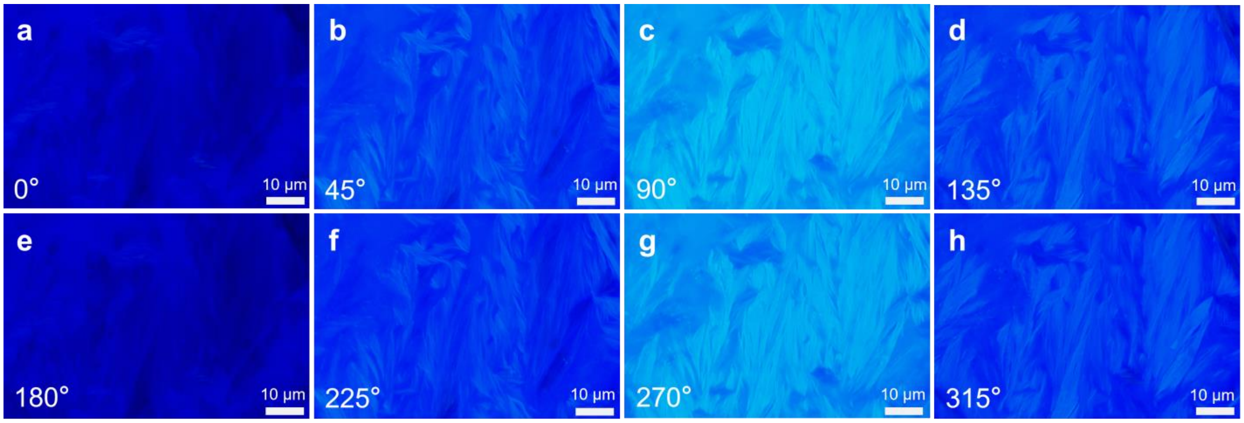

Supplementary Fig. 12 (a-h) Polarized fluorescence optical micrographs taken under crossed polarizers for film by rotating 1D SLs-OAm films by different angles from 0° to 315°. (The film was rotated in 45° increments from 0° to 315°.) Size distribution histograms were obtained from n = 25 individual NR/NPL per sample.

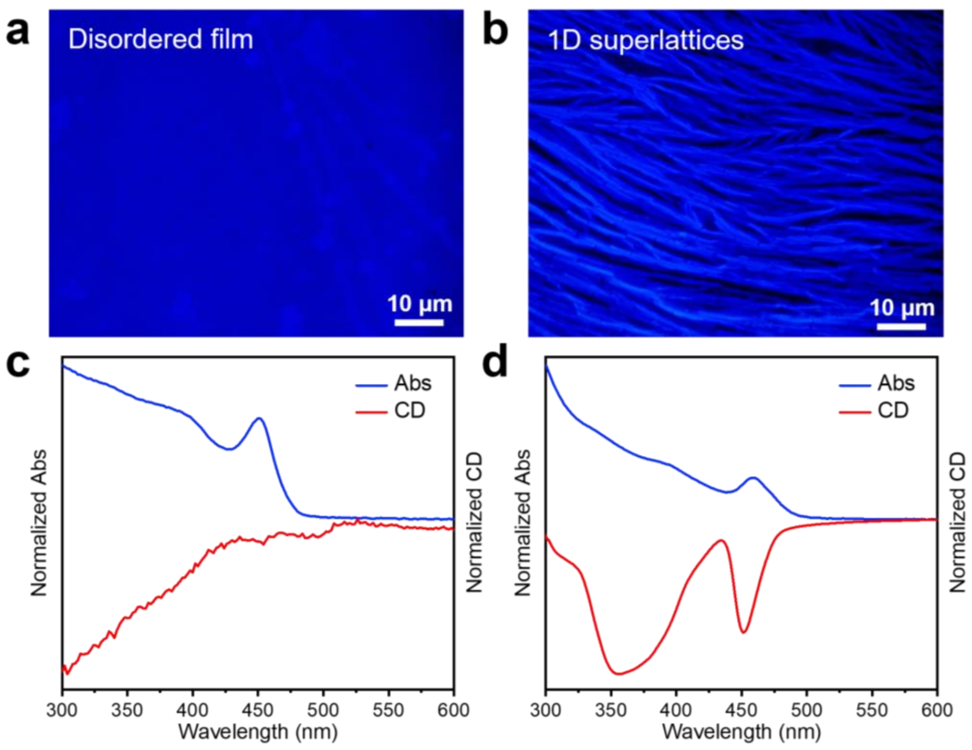

Supplementary Fig. 13 (a-b) Optical microscope images of the disordered film and the 1D SLs-OAm film. (c-d) CD signals of the disordered film and the 1D SLs-OAm film. The blue curve represents the absorption spectrum, and the red curve represents the CD spectrum. Source data are provided as a Source Data file.

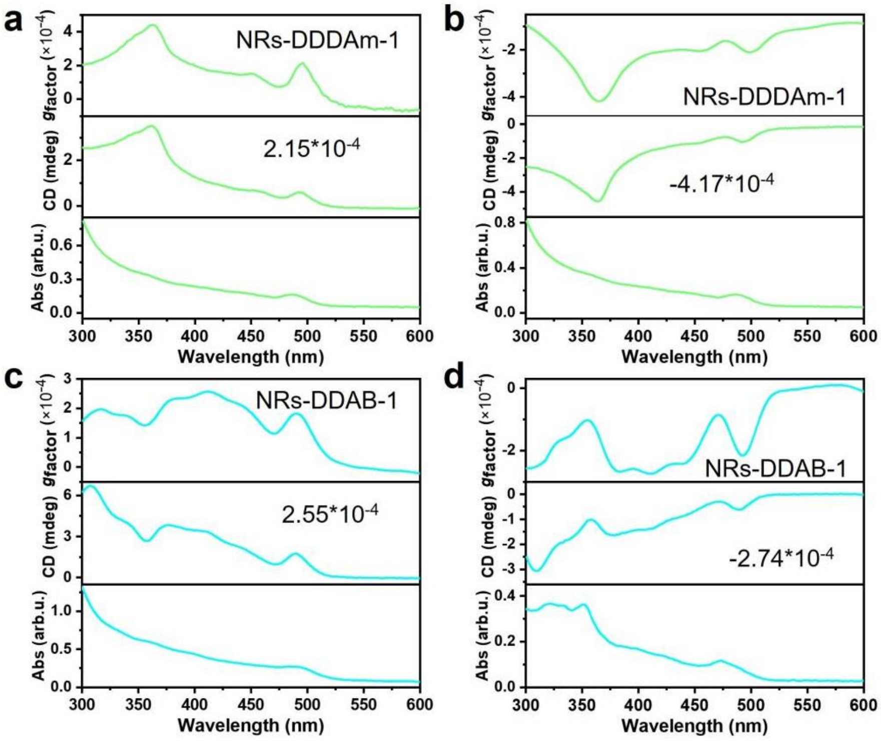

Supplementary Fig. 14 (a–b) g_{abs} data of 1D superlattices assembled from NRs-DDDAm-1; (c–d) g_{abs} data of 1D superlattices assembled from NRs-DDAB-1. The green curve represents the NRs-DDDAm-1, and the light green curve represents the NRs-DDAB-1. Source data are provided as a Source Data file.

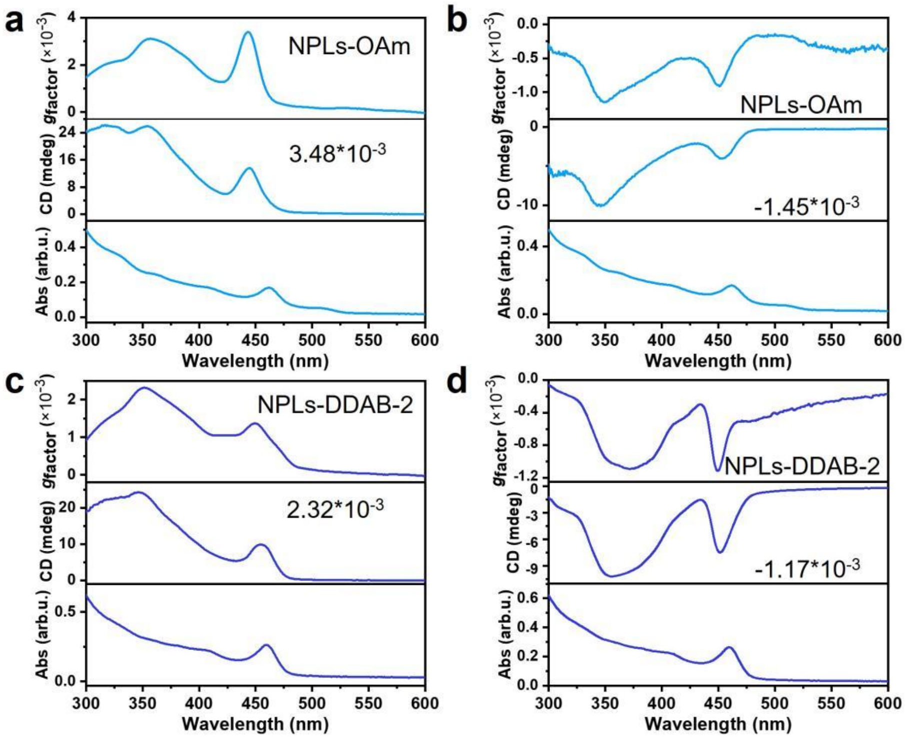

Supplementary Fig. 15 (a–b) g_{abs} data of 1D superlattices assembled from NPLs-OAm; (c–d) g_{abs} data of 1D superlattices assembled from NPLs-DDAB-2. The light blue curve represents the NPLs-OAm, and the dark blue curve represents the NPLs-DDAB-2. Source data are provided as a Source Data file.

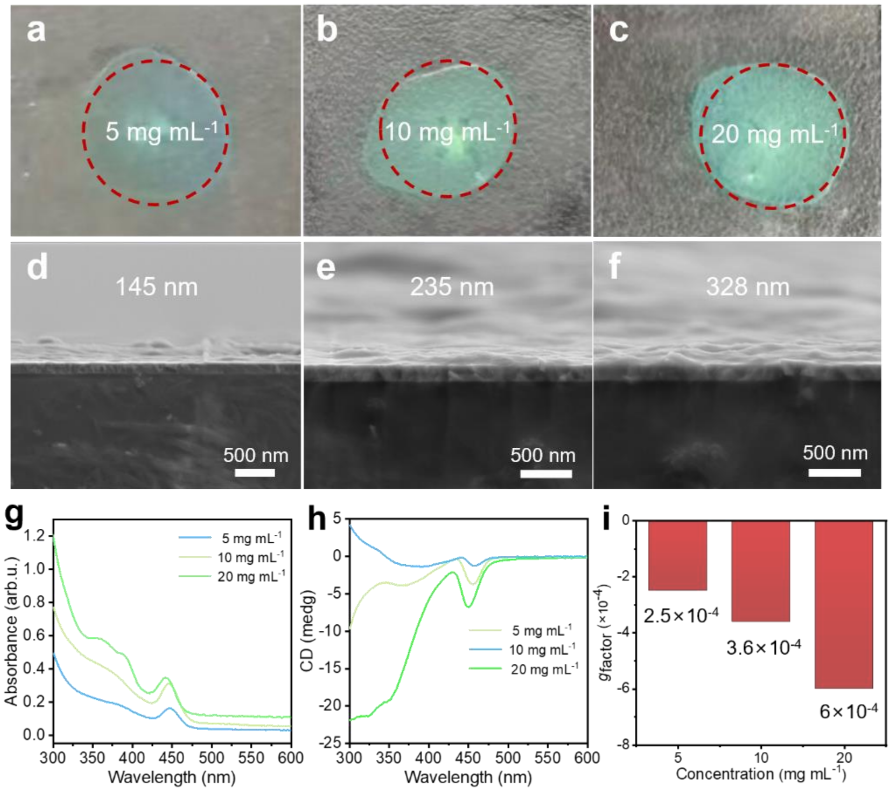

Supplementary Fig. 16 (a-c) SLs-OAm films prepared from NPLs solutions in toluene at different concentrations of NPLs, ranging from 5 to 20 mg mL^{-1}. (d-f) The corresponding thickness values for the prepared SLs-OAm films were determined by cross sectional SEM. The average thickness was 322 nm for 20 mg mL^{-1} film, 230 nm for 10 mg mL^{-1} film, and 132 nm for 5 mg mL^{-1} film, respectively. (g) Absorption spectra, (h) CD spectra, and (i) g_{abs} values of the various SLs-OAm films. Source data are provided as a Source Data file.

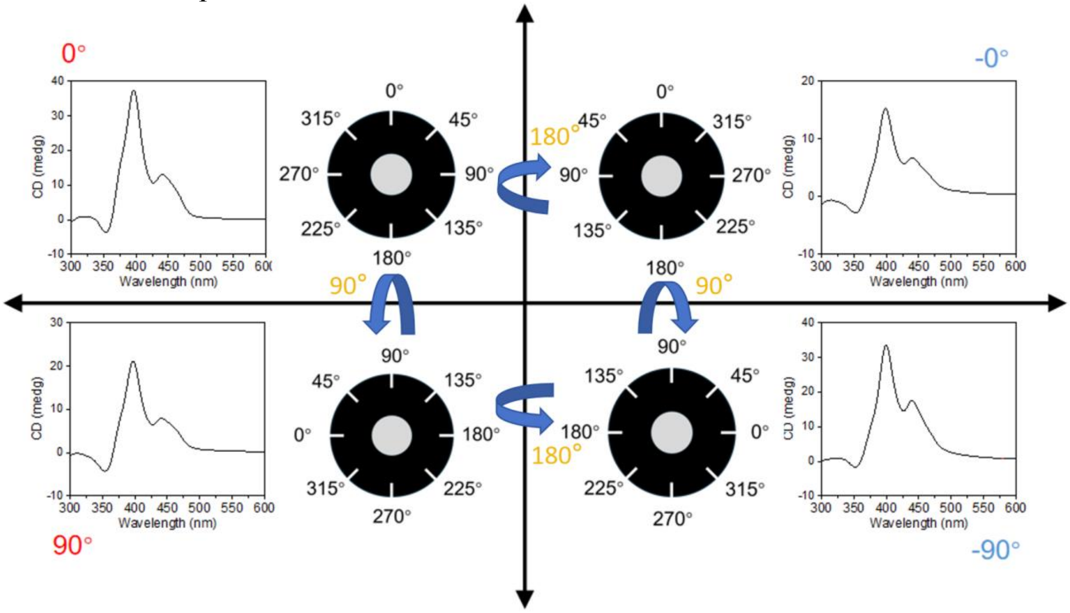

Supplementary Fig. 17 The glass slide containing the SLs-OAm was fixed on the testing frame, and the CD signals of the sample at (0°, 90°, -0°, -90°) were collected, respectively. Rotating from 0° to -0° or from 90° to -90° represents a flipping of the sample.

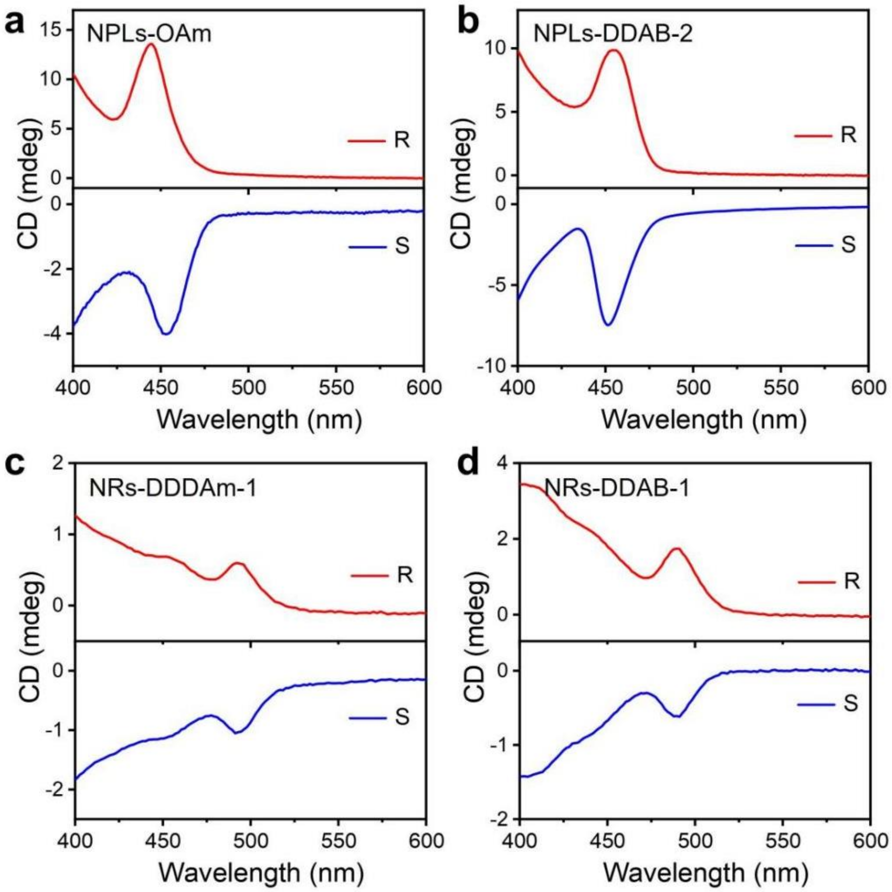

Supplementary Fig. 18 (a) Circular dichroism spectra of NPLs-OAm superlattices. (b) Circular dichroism spectra of NPLs-DDAB-2 superlattices. (c) Circular dichroism spectra of NRs-DDDAm-1 superlattices. (d) Circular dichroism spectra of NRs-DDAB-1 superlattices. All the intensity values plotted have not been normalized. The red curves represent the R-SLs, while the blue curves represent the S-SLs.

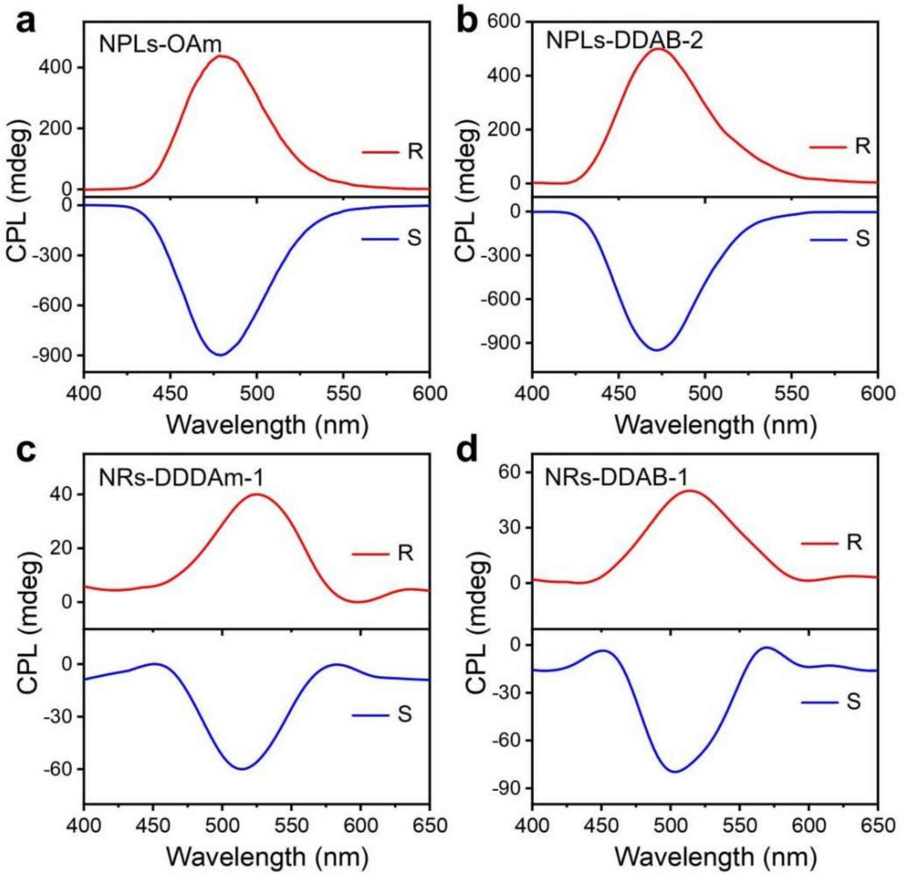

Supplementary Fig. 19 (a) Circularly polarized photoluminescence spectra of NPLs-OAm superlattices. (b) Circularly polarized photoluminescence spectra of NPLs-DDAB-2 superlattices. (c) Circularly polarized photoluminescence spectra of NRs-DDDAm-1 superlattices. (d) Circularly polarized photoluminescence spectra of NRs-DDAB-1 superlattices. All the intensity values plotted have not been normalized. The red curves represent the R-SLs, while the blue curves represent the S-SLs.

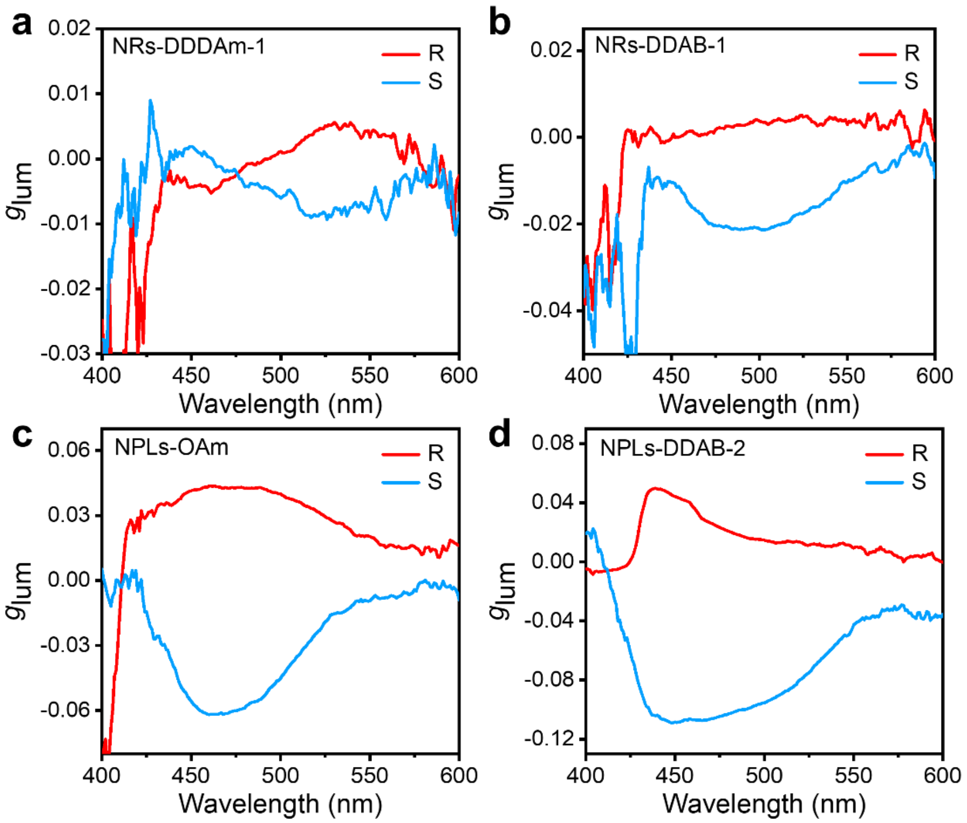

Supplementary Fig. 20 g_{lum} values of (a) NRs-DDDAm-1, (b) NRs-DDAB-1, (c) NPLs-OAm and (d) NPLs-DDAB-2 superlattices. The red curves represent the R-SLs, while the blue curves represent the S-SLs. Source data are provided as a Source Data file.

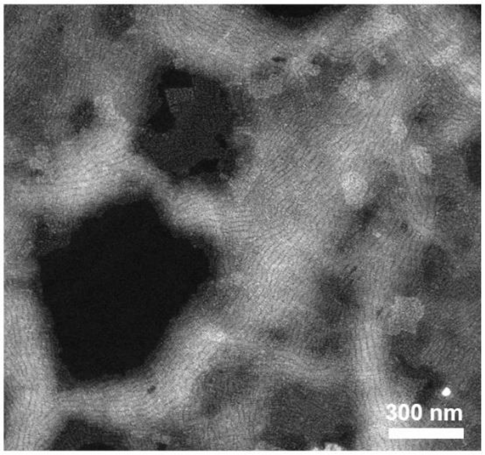

Supplementary Fig. 21 Representative TEM image of the NPLs-OAm.

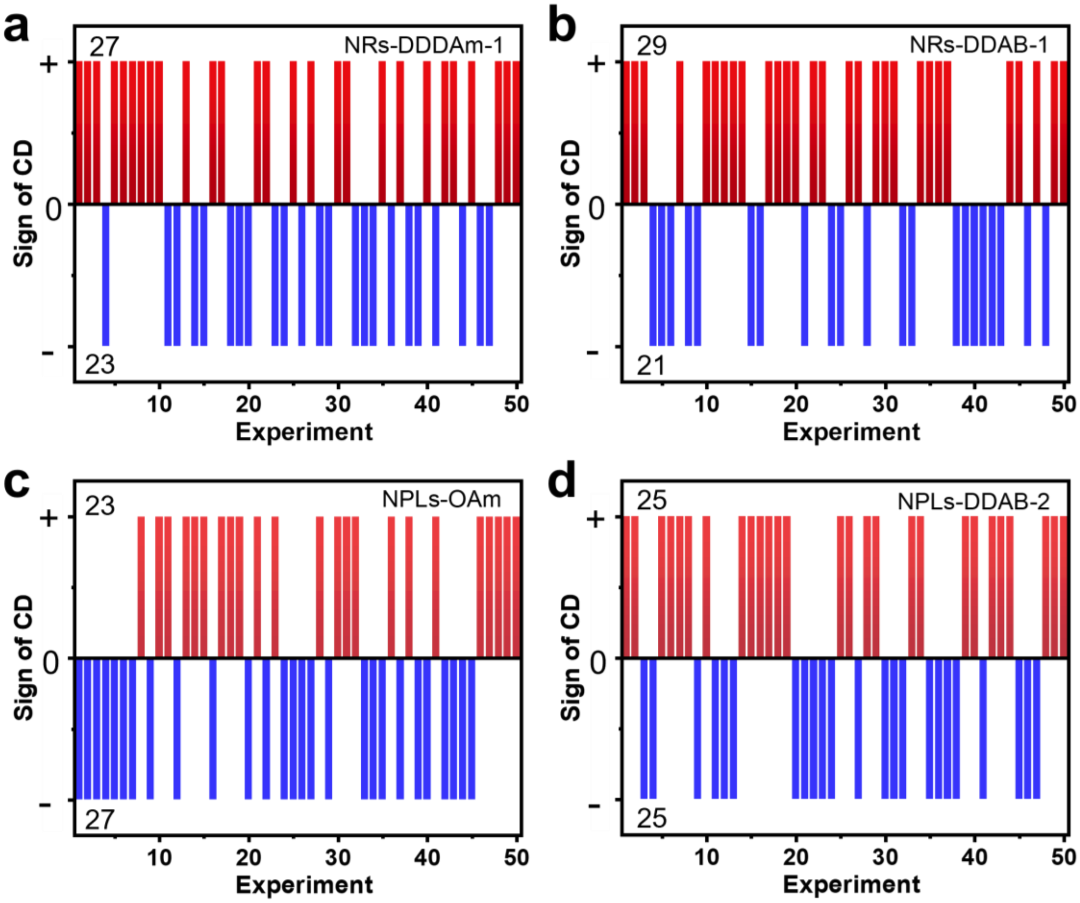

Supplementary Fig. 22 Statistical analysis of the CD signal (negative or positive) of (a) NRs-DDDAm-1, (b) NRs-DDAB-1, (c) NPLs-OAm and (d) NPLs-DDAB-2 superlattices without addition of chiral ligands. The positive CD signal (red bar) was counted as +, and the negative signal (blue bar) was counted as -. Source data are provided as a Source Data file.

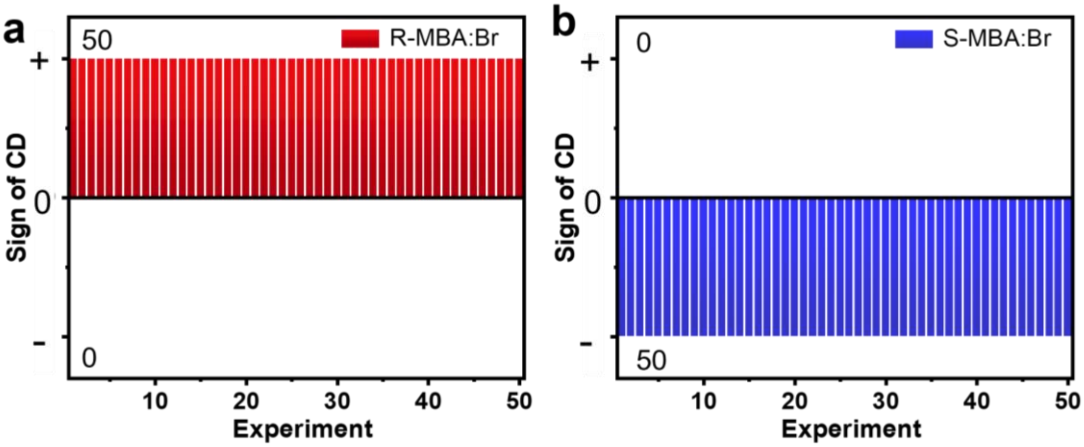

Supplementary Fig. 23 Statistical analysis of CD signal of NRs-DDDAm-1 superlattices after treatment with chiral ligands (R/S-MBA:Br). The positive CD signal (red bar) was counted as +, and the negative signal (blue bar) was counted as -. Source data are provided as a Source Data file.

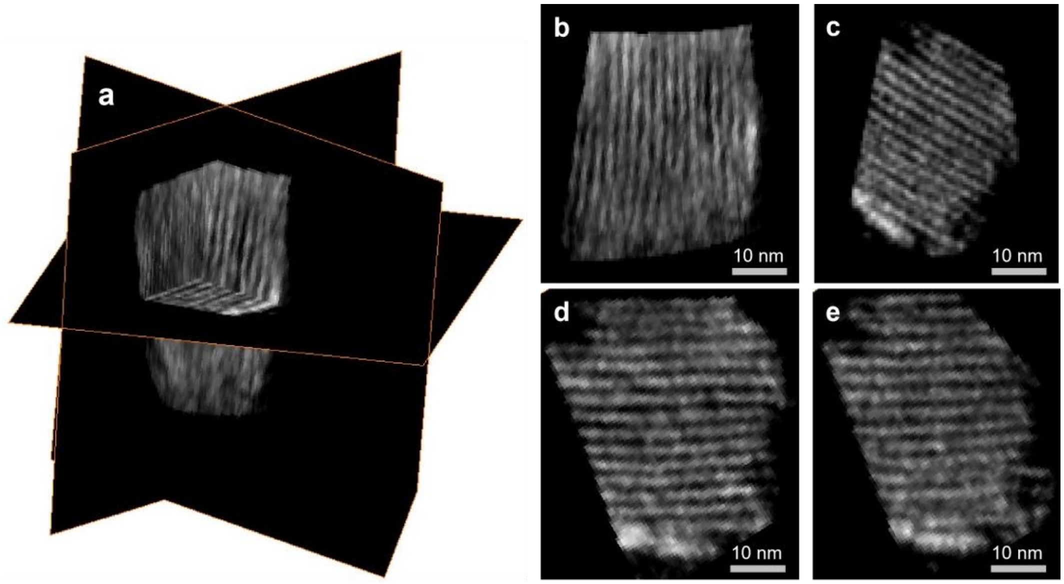

Supplementary Fig. 24 3D reconstruction of the superlattices obtained by electron tomography, showing the reconstructed 3D volume and orthoslices from different projections.

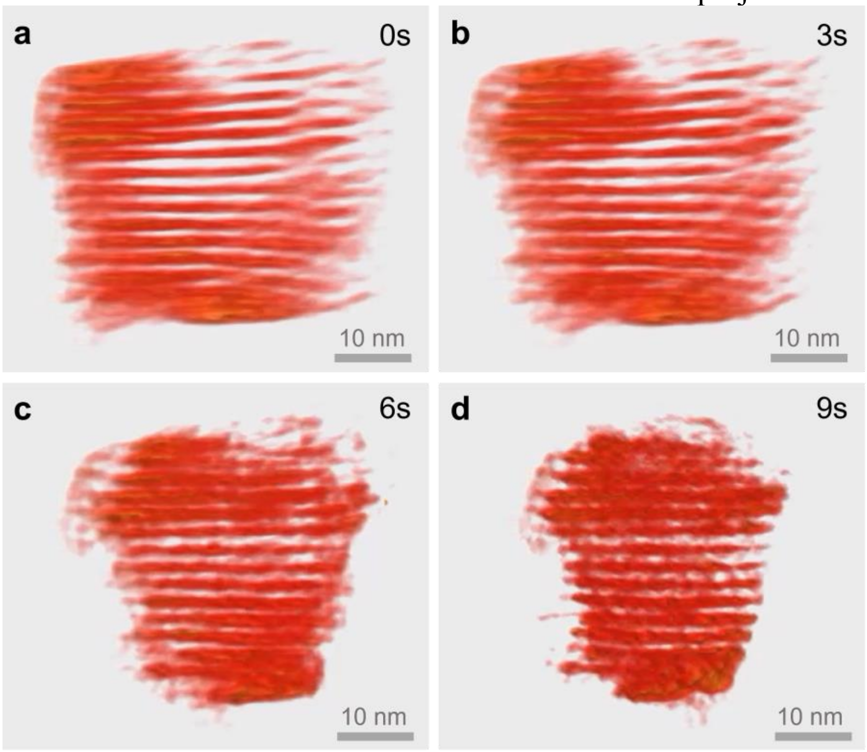

Supplementary Fig. 25 Sequential frames from this movie at (a) 0 s, (b) 3 s, (c) 6 s, and (d) 9 s.

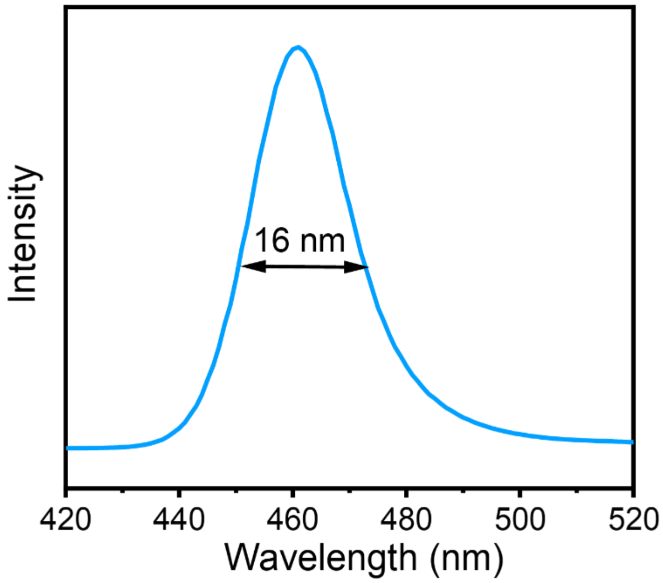

Supplementary Fig. 26 Photoluminescence spectra of a randomly assembled NPLs-OAm film by laser pumping under identical parameters as for the measurement of NPLs-OAm superlattices. The full width at half maximum (FWHM) is calculated as 16 nm. Source data are provided as a Source Data file.

Supplementary Table 1 Comparison of aspect ratio (AR), polarized emission (P), and circularly polarized luminescence ($|g_{lum}|$) between 1D superlattices assembled from $CsPbBr_3$ nanorods (SLs-NRs) and nanoplatelets (SLs-NPLs).

	Aspect ratio (AR)	Polarized emission (*P*)	Circularly polarized luminescence ($|g_{lum}|$)
SLs-NRs	5	0.72	0.02
SLs-NPLs	7.7	0.93	0.11

Supplementary references:

1. Toso S, Baranov D, Giannini C, Marras S, Manna L. Wide-Angle X-ray Diffraction Evidence of Structural Coherence in CsPbBr3 Nanocrystal Superlattices. *ACS Materials Letters* **1**, 272-276 (2019).

2. Gomes Ferreira M*, et al.* Self-Assembly of Quantum-Confined CsPbBr3 Perovskite Nanocrystals into Rhombic, Frame, and Rectangular Superlattices. *Small Structures* **6**, 2500133 (2025).
