# Supplementary material for: One-dimensional CsPbBr3 superlattices with polarized and amplified spontaneous circularly polarized emissions
Source: Nat Commun. 2026 May 23;17:6768. doi: 10.1038/s41467-026-73513-2 (PMC13385850; doi:10.1038/s41467-026-73513-2)
Supplement: Supplementary file 2 — Description of Additional Supplementary Files [file 41467_2026_73513_MOESM2_ESM.pdf]

Description of Additional Supplementary Files

Supplementary Video 1. 3D reconstruction video of the superlattices obtained by electro
n tomography.

Supplementary Data 1. Unprocessed raw data
