# Supplementary figures and images for: One-dimensional CsPbBr3 superlattices with polarized and amplified spontaneous circularly polarized emissions
Source: Nat Commun. 2026 May 23;17:6768. doi: 10.1038/s41467-026-73513-2 (PMC13385850; doi:10.1038/s41467-026-73513-2)

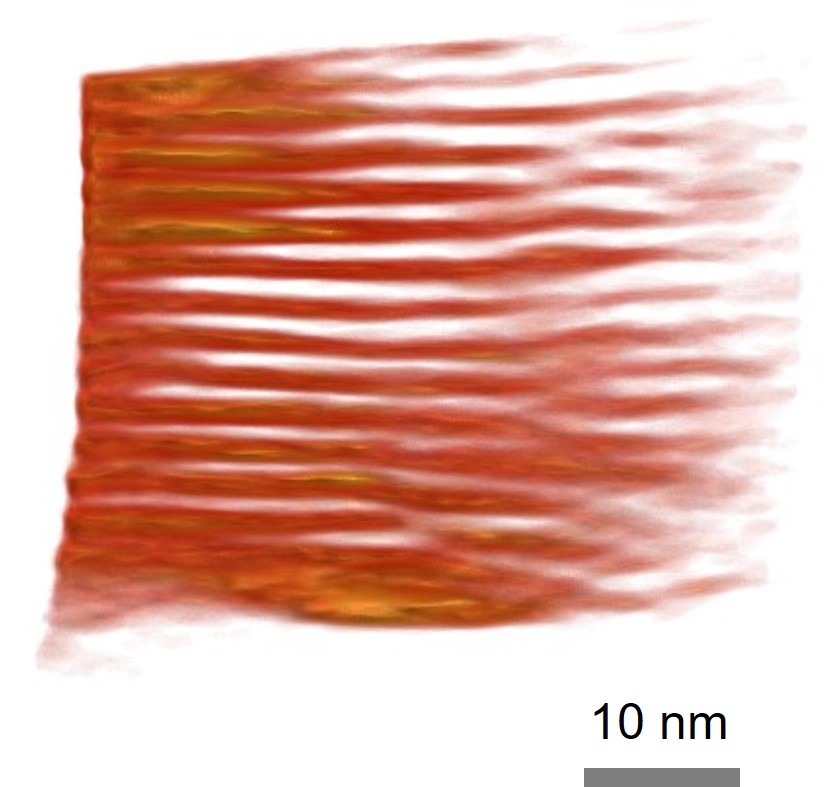

Supplement: Supplementary file 4 — Supplementary Data 1 (Unprocessed data) [file 41467_2026_73513_MOESM4_ESM.zip › Supplementary Data 1/Raw Data/Manuscript-Raw Data/Figure 6/ET data/ET1.jpg]

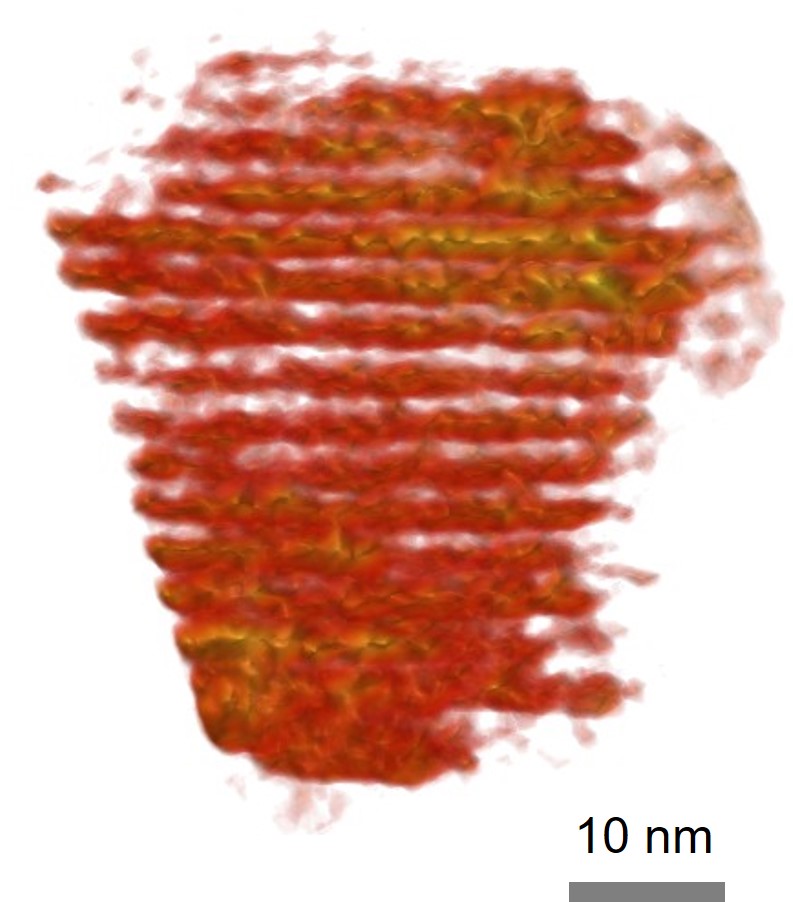

Supplement: Supplementary file 4 — Supplementary Data 1 (Unprocessed data) [file 41467_2026_73513_MOESM4_ESM.zip › Supplementary Data 1/Raw Data/Manuscript-Raw Data/Figure 6/ET data/ET2.jpg]

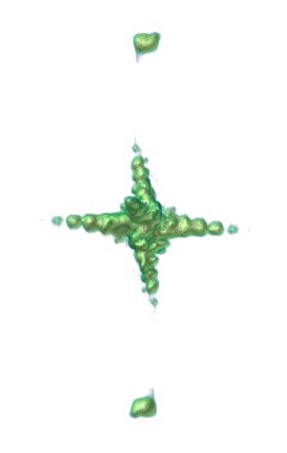

Supplement: Supplementary file 4 — Supplementary Data 1 (Unprocessed data) [file 41467_2026_73513_MOESM4_ESM.zip › Supplementary Data 1/Raw Data/Manuscript-Raw Data/Figure 6/ET data/volume1-fft.jpg]

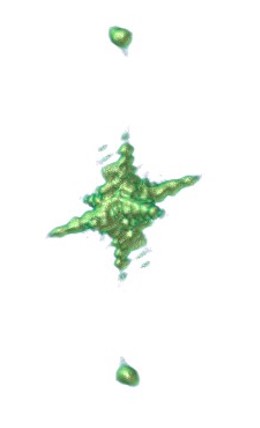

Supplement: Supplementary file 4 — Supplementary Data 1 (Unprocessed data) [file 41467_2026_73513_MOESM4_ESM.zip › Supplementary Data 1/Raw Data/Manuscript-Raw Data/Figure 6/ET data/volume2-fft.jpg]

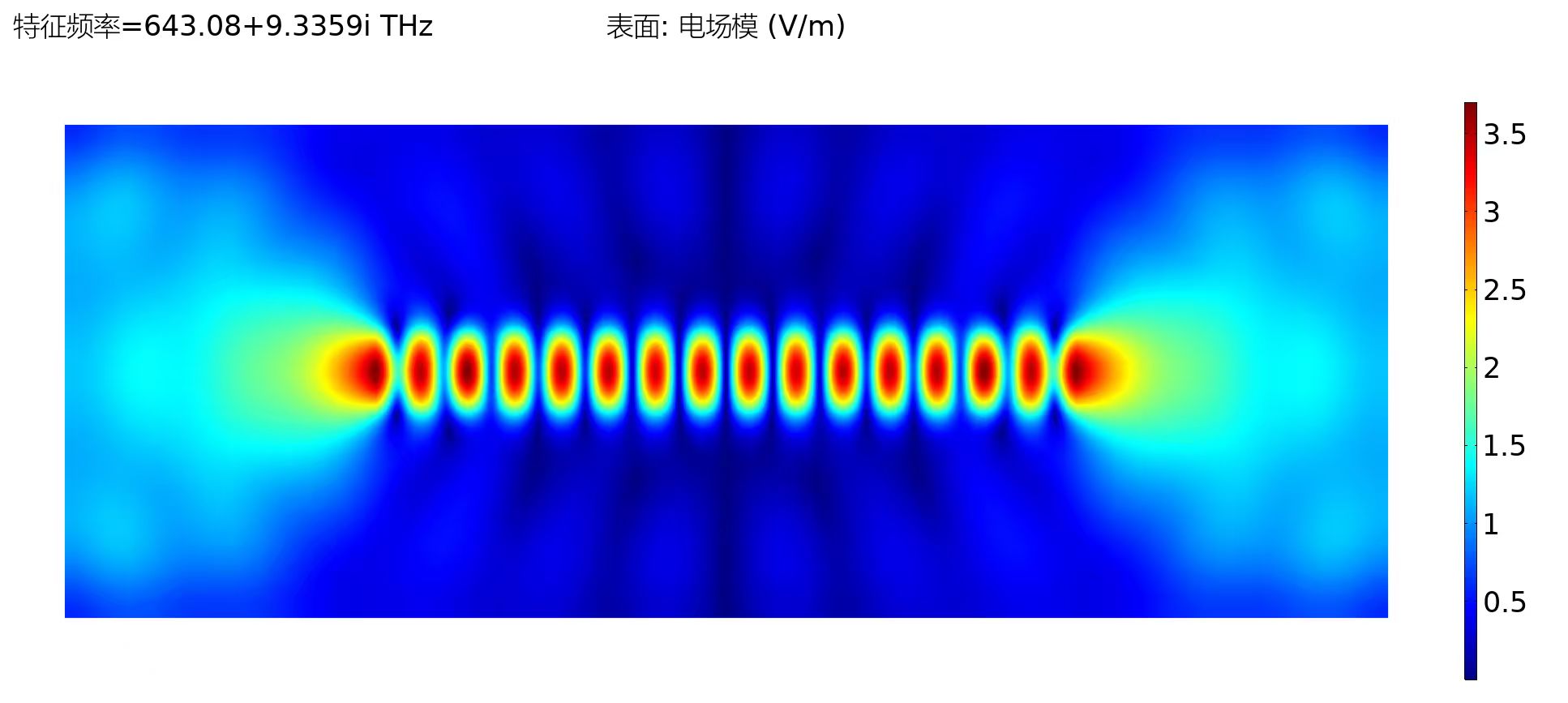

Supplement: Supplementary file 4 — Supplementary Data 1 (Unprocessed data) [file 41467_2026_73513_MOESM4_ESM.zip › Supplementary Data 1/Raw Data/Manuscript-Raw Data/Figure 7/4ee1818a6653553058f3112e683bf865.jpg]

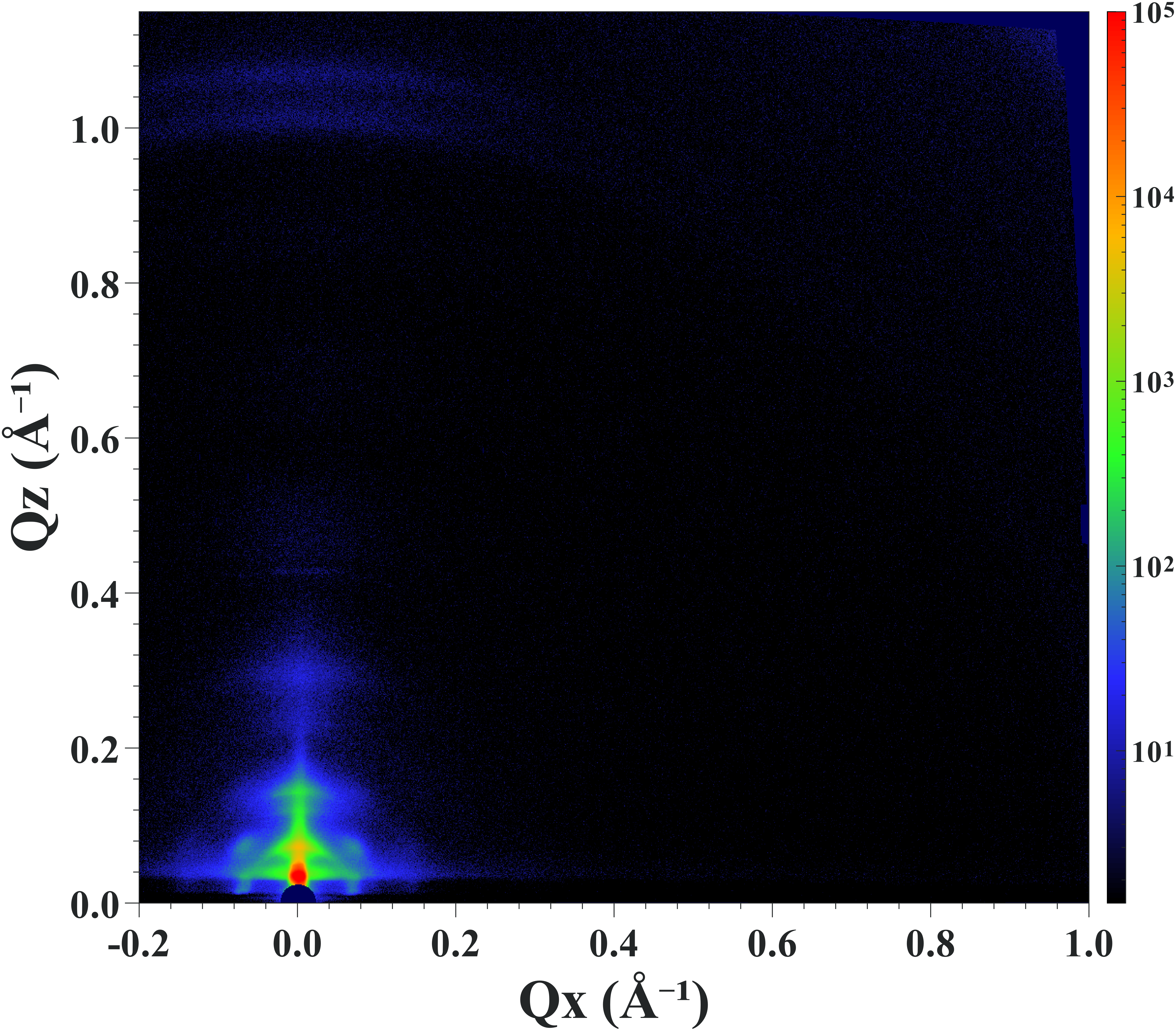

Supplement: Supplementary file 4 — Supplementary Data 1 (Unprocessed data) [file 41467_2026_73513_MOESM4_ESM.zip › Supplementary Data 1/Raw Data/SI-Raw Data/Fig 10/Fig 10a-SAXS/3. 二维数据 png/20241113_0_vd_00014-00015_corr1XZ.png]

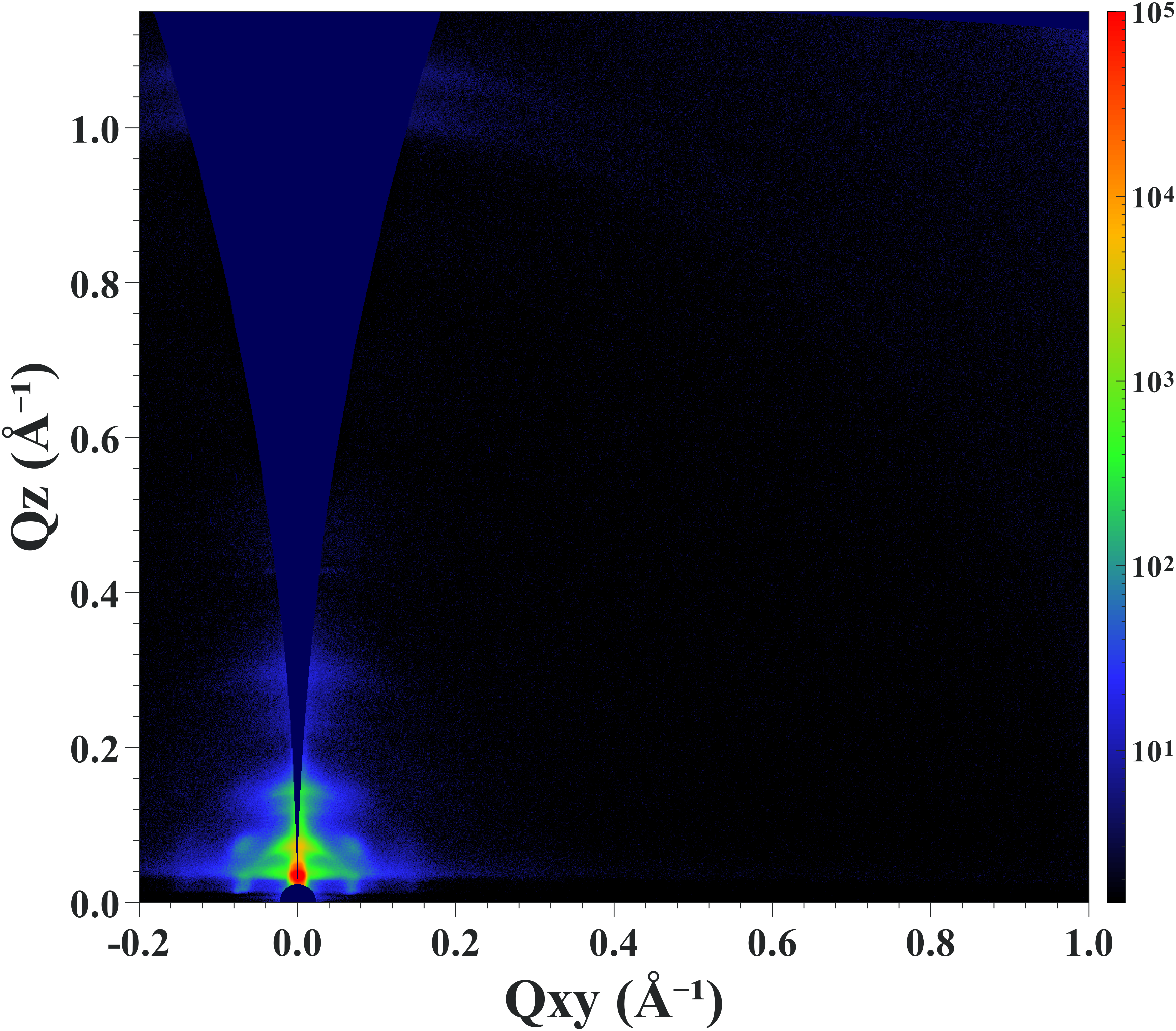

Supplement: Supplementary file 4 — Supplementary Data 1 (Unprocessed data) [file 41467_2026_73513_MOESM4_ESM.zip › Supplementary Data 1/Raw Data/SI-Raw Data/Fig 10/Fig 10a-SAXS/3. 二维数据 png/20241113_0_vd_00014-00015_corr3XYZ.png]

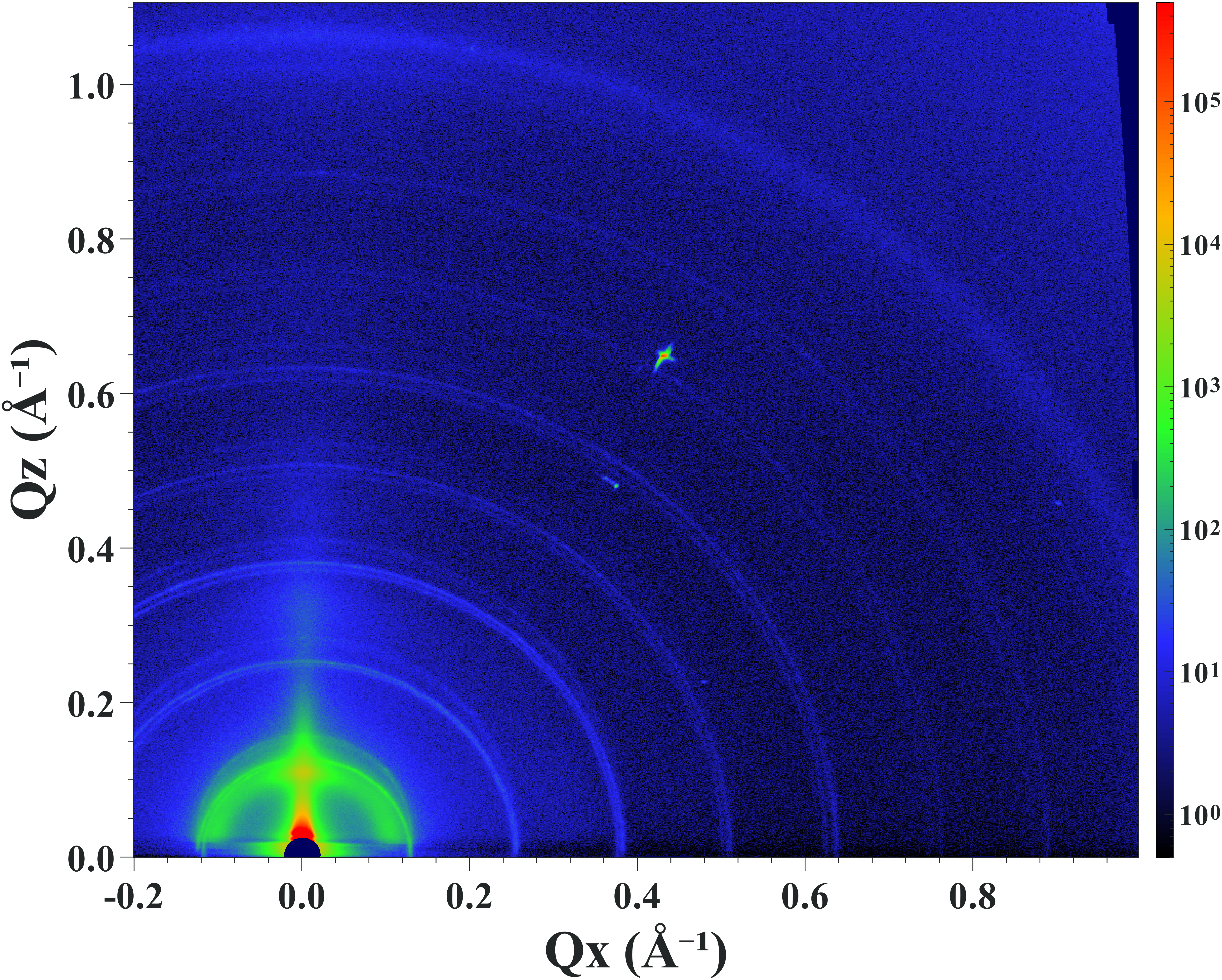

Supplement: Supplementary file 4 — Supplementary Data 1 (Unprocessed data) [file 41467_2026_73513_MOESM4_ESM.zip › Supplementary Data 1/Raw Data/SI-Raw Data/Fig 10/Fig 10b-SAXS/3. 二维数据 png/20250613_0_vd_00073-00074_corr1XZ.png]

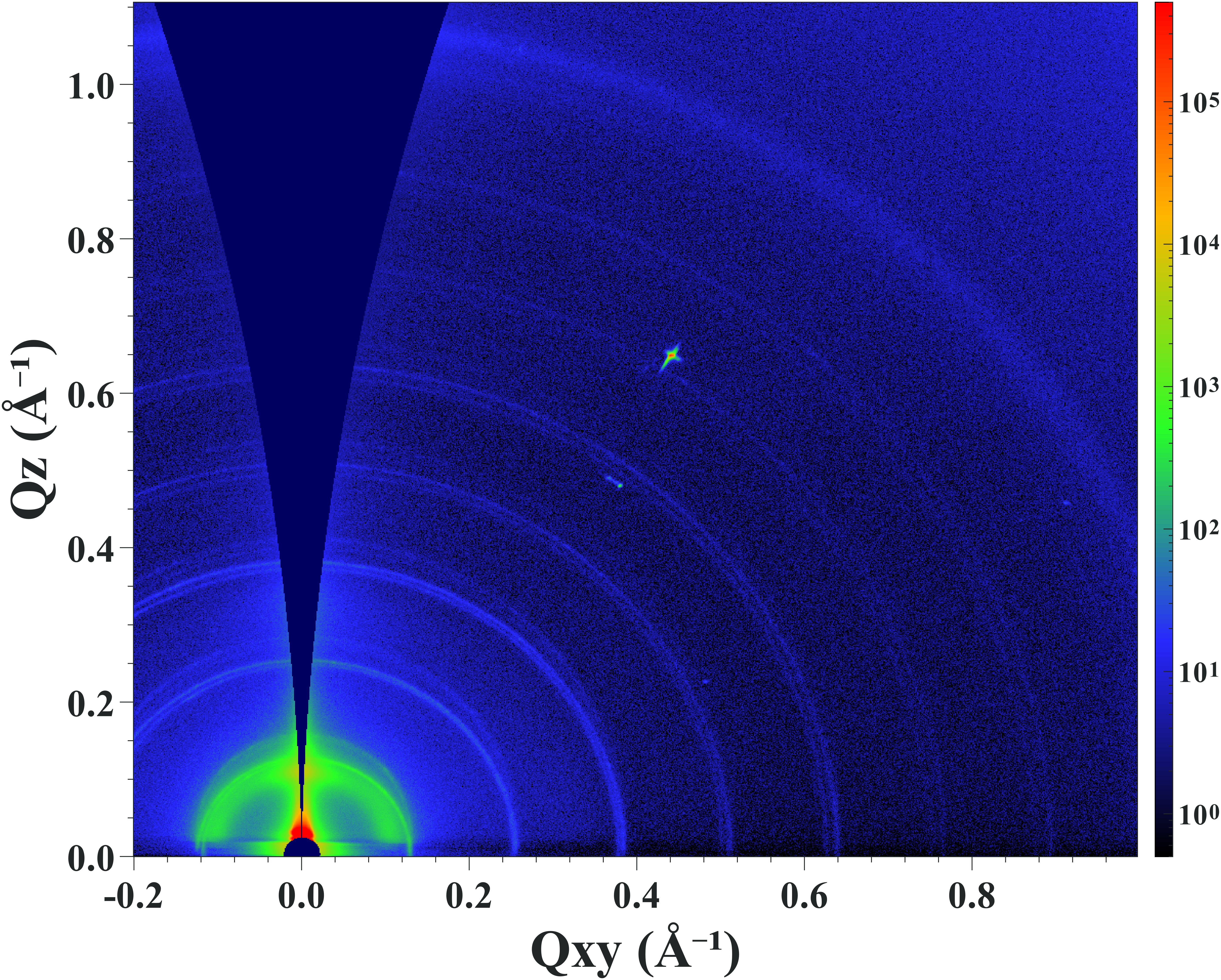

Supplement: Supplementary file 4 — Supplementary Data 1 (Unprocessed data) [file 41467_2026_73513_MOESM4_ESM.zip › Supplementary Data 1/Raw Data/SI-Raw Data/Fig 10/Fig 10b-SAXS/3. 二维数据 png/20250613_0_vd_00073-00074_corr3XYZ.png]
