# Supplementary material for: One-dimensional CsPbBr3 superlattices with polarized and amplified spontaneous circularly polarized emissions
Source: Nat Commun. 2026 May 23;17:6768. doi: 10.1038/s41467-026-73513-2 (PMC13385850; doi:10.1038/s41467-026-73513-2)
Supplement: Supplementary file 5 — Author Checklist [file 41467_2026_73513_MOESM5_ESM.docx]

| 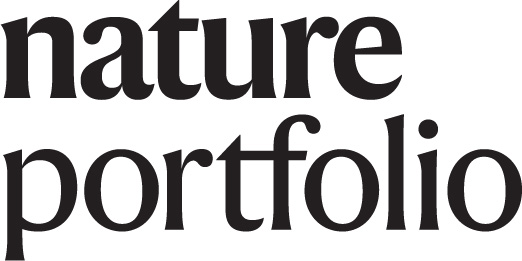 |  |
| --- | --- |

| **Author Checklist** | Manuscript Number: |
| --- | --- |
|  | NCOMMS-25-87032A-Z |
|  | ______________________________ |
| **Please check the items below carefully and add a response in each row of the table to indicate the changes that you have made. Please also check through any additional marked-up edits we may have provided within the manuscript file.** |  |
|  |  |
|  |  |
|  |  |
| **Abstract and editor's summary** |  |
| Our guidance: | Your response: |
| We would like to propose a revised title to comply with our formatting requirements and improve the accessibility of your work:  **One-dimensional CsPbBr₃ nanocrystal superlattices with polarized and amplified spontaneous circularly polarized emissions**  Please edit the title in your manuscript files accordingly. We invite you to propose an alternative title. Note that further minor changes may be made during the production process, and you will be able to check these in the proofs | We have revised it accordingly. |
| The abstract — which should be roughly **150-200 words** long and contain no references — should serve both as a general introduction to the topic and a non-technical summary of your main results and their implications. It should contain a brief account of the background and rationale of the work, followed by a statement of the main conclusions introduced by the phrase 'Here we show' or some equivalent phrase. Because we hope that researchers in a wide range of disciplines will be interested in your work, the abstract should be as accessible as possible, explaining essential but specialized terms concisely. We encourage you to show your abstract to colleagues outside of your direct field of expertise to uncover any problematic concepts. | We have checked it. |
| When discussing the current work in the abstract, please use the present tense. | We have checked it. |
| L30-31, please remove “Keyword: Halide perovskite nanocrystals; nanoplatelets; one-dimensional superlattices; Polarization; Chirality” | We have revised it. |
| L21, please change “near unity degree of polarization” to “near-unity degree of polarization” | We have revised it. |
| L22, and in all other instances, please use "≈" to signify “approximately” (instead of "~"). Please use “~” to signify “on the order of”. | We have revised it. |
| L27, and in all other instances, please set “g” in italics. | We have revised it. |
|  |  |
| **Author information** |  |
| Our guidance: | Your response: |
| We ask that you consult with your coauthors to ensure that all names, affiliations, and titles are represented correctly. Note that if any authors are added or removed after this point then all authors will be requested to provide approval documentation that could potentially delay the production of your paper. | We have checked it. |
| Ensure affiliations are appropriately labeled and featured sequentially and in ascending order (1,2,3,... or a,b,c...). Please ensure all corresponding authors are marked with a specific symbol and include their emails. Similarly, if you have “equally contributing” or “joint supervision” authors, use a specific symbol to mark them and not a number. | We have revised it. |
| Please ensure the author contributions section mentions each author's initials at least once with their contributions to the work. Authors with the same initials must be differentiated in the statement. | We have revised it. |
|  |  |
| **Article structure** |  |
| Our guidance: | Your response: |
| We can accommodate up to 10 display items (Figures or Tables) in the main article. Each Figure and Table must fit easily within an A4 page (210 x 297 mm). Please ensure that the number and size of your Figures and Tables fulfil these requirements to avoid any delay in the acceptance of your article. | We have checked and revised it. |
| Ensure main Figures are uploaded as separate individual files. Each figure file must contain all intended panels labelled and displayed as intended and fit entirely on a single page. Do NOT include legends within the figure files, as these must be in the main manuscript.  Supplementary Figures must be all contained in the Supplementary Information PDF and do NOT need to be uploaded separately. | We have fully adjusted our figures and tables following your guidelines.All main figures are provided as independent single-page files, legends are kept in the manuscript, and supplementary figures are combined in the SI PDF.The font type, size and layout of all graphics have been standardized to comply with journal requirements. |
| **To comply with this format and optimise the presentation of data in your Article, we suggest the following changes to the display items in your paper:** |  |
| *When scaling to our article template, text and details in some images may be too small or too large. Please provide your final figures at article scale and as a guideline,* ***all text should be sans-serif typeface, preferably Helvetica or Arial. Maximum text size is 7 pt. Minimum text size is 5 pt. Please ensure a consistent font and font size throughout all Figures.***  For detailed guidance on figure preparation, see: https://www.nature.com/documents/NRJs-guide-to-preparing-final-artwork.pdf | We have checked and revised it. |
| **Please ensure your main manuscript file includes the following sections, in this order:** |  |
| *Title Author list Affiliations Abstract Introduction Results Discussion (optional) Results and Discussion (optional) Methods Data Availability Code Availability (if relevant) References Acknowledgements Author Contributions Statement Competing Interests Statement Tables Figure Legends/Captions (for main text figures)* | We have checked and revised it. |
| We do not edit Supplementary Information files; they will be uploaded with the published article as they are submitted with the final version of your manuscript. Any tracked changes should be removed from the file and the file should be provided as a PDF file. Supplementary Figures do not need to be provided separately. | We have deleted all tracked changes in the SI file and supplied it in PDF format. All supplementary figures are included within the SI PDF and will not be uploaded separately. |
|  |  |
| In the Supplementary Information file, please rename the references to “Supplementary references”.  In the Supplementary Information file, please rename Figures to “Supplementary Figures” and Tables as “Supplementary Tables”.  Please note **that all text sections in the Supplementary Information file** should be organized, titled, and referred to as “Supplementary Notes” or “Supplementary Discussion”. Please refer to our “Formatting Instructions” for further guidance.  In the SI file, please add page numbers.  **Experimental details provided in the Supplementary Information should be included in the Methods section in the main article, in a concise version giving all necessary details.** Sufficient details of the experiments must be provided in the Methods section such that they could be reproduced without reference to published papers. Use of the term "as described previously" (or similar wording) is not encouraged. **Please transfer the respective text sections from the Supplementary Information that give experimental details and characterization procedures to be included in the methods section in the main article.** This refers to the section “Materials and Methods” (which needs to be rename to Methods, p.2-5. **To clarify, this only refers to the text sections that contain experimental details and descriptions of methods**, but the Suppl. Figures and Suppl. Tables should not be transferred and remain in the Suppl. Information file.  **While we do not impose a hard limit for the length and word count in the Methods section, we recommend rephrasing the text, as much as possible, to make it more concise without omitting important information.**  Please consider incorporating free text in the Suppl. Information file that directly provides additional information about Suppl. Figures in the corresponding Suppl. Figs., whenever possible.  **We discourage using the S- prefix to refer to Supplementary Items.** Instead, please write “Supplementary Figure #” or “Suppl. Fig. #” or “Supplementary Table #” or “Suppl. Table #”. | We have checked and revised it accordingly. |
| **Please supply Source Data files (i.e., the numerical raw data that was used for plotting) for all data presented in graphs within the Figures and Supplementary Figures.**  We are aware there are tools that can extract values from images, but these hinder both accessibility and accuracy of the extracted data, therefore we require authors to also provide the underlying data for all plots/graphs in the manuscript and supplementary information. Please note that Source Data Files should be supplied separately from potential Supplementary Data files containing, e.g., raw data. | We have provided independent Source Data files including the full raw numerical data for all graphs in the main and supplementary figures, in compliance with your requirements. |
| Within the Source Data file, the relevant numerical data from each figure or table (in the main manuscript and in the Supplementary Information) should be represented by a single sheet in an Excel document, or a single .txt file or other file type in a zipped folder. An example of the Source Data file is available demonstrating the correct format:  https://www.nature.com/documents/ncomms-example-source-data.xlsx  The file should be labelled 'Source Data', with the title and a brief description included in your response here, and **should be mentioned in all relevant Figure and Suppl. Figure legends using the template text below:**  **'Source data are provided as a Source Data file.'**  A reference to the source data file should be added in the 'Data Availability' section, using the text **“Source data are provided with this paper.**”. | We have prepared and uploaded the Source Data file in accordance with the required format.The file contains all numerical data for all figures and tables in the main manuscript and Supplementary Information, with each figure/table presented in a separate Excel sheet.  The statement “Source data are provided as a Source Data file” has been added to all relevant figure and Supplementary Figure legends.In addition, the sentence “Source data are provided with this paper.” has been included in the Data Availability section. |
| **Unprocessed raw data**: Please provide the unprocessed raw data corresponding to all results presented in the manuscript and the Supplementary Information file as **unprocessed data**, compiled in a **Supplementary Data file** (e.g., zip folder of txt or csv files). In addition, please ensure that the Methods section includes a complete and **detailed description of all data processing steps** performed on the data. This should include, but not be limited to, background subtraction, smoothing, signal averaging, curve fitting, normalization, and any other processing procedures. Please also specify any software or custom scripts used. This information is essential to ensure reproducibility and align with best practices in data transparency. | We have supplied all unprocessed raw data as a Supplementary Data zip file. The Methods section has been revised to include full details of data processing steps, software, and scripts as required. |
|  |  |
| **General guidance and main text** |  |
| Our guidance: | Your response: |
| **Please refrain from using words such as new/novel/first/unique, when referring to the scientific findings as novelty can be inferred by context.**  **Please also** **remove exaggerated and subjective language** and claims such as ‘extreme’, 'extremely', ‘giant’, “ultra”, ‘impressive’, 'outstanding', 'fascinating', ‘tremendous’, ‘holistic’, ‘powerful’, 'pave the way', ‘elegant’, ‘strikingly’, ‘unconventional’, ' open up a splendid era', 'to the best of our knowledge', ‘ultimate’, ‘surprisingly’, ‘remarkable’, ‘notably’ ‘record’, ‘surprising’, 'unprecedented', ‘open new avenues’, ‘paves the way’, ‘open the window’, ‘next generation’, and similar terminology. | We have checked and revised it. |
| Please divide the Results section into subsections, each with a title of 60 characters or fewer **including spaces.** | We have checked and revised it. |
| Please write units with **negative exponents**, e.g., “g mL^-1” instead of “g/mL”, throughout the manuscript, Figures, Tables, and the Supplementary Information. | We have checked and revised it. |
| **Please define abbreviations only once and at the first use**. Please revise throughout the manuscript. | We have checked and revised it. |
| **In Figures and Suppl. Figures, please use panel markers following the a, b, c, etc. convention, instead of (a), (b), (c), etc. Also, please set panel markers in bold.** | We have checked and revised it. |
| **In Figures, Tables, and throughout the manuscript, we discourage giving units in square brackets "[]", e.g., in axis labels as these brackets are used already to signify concentrations. We recommend to use round brackets "()", e.g. "ΔG (kcal mol^-1)" instead of "ΔG [kcal .mol-1]".** | We have checked and revised it. |
| **Please ensure that values and units are separated by a small space, e.g., “5 mL” instead of “5mL”.** | We have checked and revised it. |
| Both in the main article and in the Suppl. Information file, please use "≈" to signify “approximately” (instead of "~"). Please use “~” to signify “on the order of”. Please use “∝” to signify “proportional to”. | We have checked and revised it. |
| Please remove "⋅" in units, e.g., "J cm^-2" instead of "J⋅cm-2". | We have checked and revised it. |
| Please use bold font for numbering chemical compounds, **but not for chemical abbreviations or formulae in both the main text and the display items.** | We have checked and revised it. |
| Both in the main manuscript as well as in the Suppl. Information file, please label equations sequentially as (1), (2), (3), etc. | We have checked and revised it. |
| A full Methods section, divided into subsections and subheadings, must be provided in the main manuscript. There is no word limit to this section. | We have checked and revised it. |
| Please remove or rename the Conclusion heading, as the main text should only include the sections Abstract, Introduction, Results, optional Discussion and optional Methods. We also allow a combined Results and Discussion section. | We have checked and revised it. |
| **Please do not use italics, bold font, underlining or speech marks except in headings unless required for technical terms (in both the main text and the display items).** | We have checked and revised it. |
| **Please make sure that mathematical terms throughout your manuscript and Supplementary Information (including in figures, figure axes, and legends) conform strictly to the following guidelines.** Equations must be supplied in editable format, and not as images. Scalar variables (e.g., x, V, χ) must be typeset in italic, whereas multi-letter variables and functions (e.g., log, cos, PLQY) must be formatted in roman. Vectors (such as the wavevector k or the magnetic field vector B) must be typeset in bold without italics.  **Single-letter variables** must be typeset in italics, while **multi-letter variables** must be typeset in roman. Units that refer to numerical values or constants must be typeset in roman, e.g., “nm” or “mL”. Text indices and numerical indices must be typeset in roman, e.g., “abs” or “exc”; this applies both to super- as well as subscripts.  **Physical constants**, such as Boltzmann k, Planck h, or gas constant R, should be set in roman instead of italics.  **Numerical constants**, such as micro μ, milli m, kilo k, or mega M, should be set in roman instead of italics. | We have checked and revised it. |
| L41, please avoid “unique” | We have checked and revised it. |
| L43, please clarify “minibands” | We have clarified the term “minibands” in text with detailed explanation:narrow secondary energy bands arising from interparticle electronic hybridization and energy splitting. |
| L46, please change “long range order” to “long-range order” | We have checked and revised it. |
| L60, 61, 93, 103, 105, 112, 117, 118, 142, 239, and in all other instances, please use "≈" to signify “approximately” (instead of "~"). Please use “~” to signify “on the order of”. | We have checked and revised it. |
| L67, and in all other instances, please set “P” in italics. | We have checked and revised it. |
| L74, 76, 220, 229, 230, and in all other instances, please set “g” in “g_lum” and “g_abs” in italics. | We have checked and revised it. |
| L80, and in all other instances, please set single-letter variable “d” in italics. | We have checked and revised it. |
| L87, please change “Discussion” to “discussion” | We have checked and revised it. |
| L89, please change “in the method section of the Supporting Information (SI).” to “in the Methods section.” | We have checked and revised it. |
| L94, 96, 100, 101, 123, 125, 191, and in all other instances, please change “Figure S#” to “Supplementary Fig. #” | We have checked and revised it. |
| L97, 158, 224, and in all other instances, please change “mg/mL” to “mg mL^-1” with “-1” as superscript. | We have checked and revised it. |
| L102, please change “hours” to “h” when used as a unit in conjunction with a number. | We have checked and revised it. |
| L113, please change “Figure 1e and 1h” to “Figure 1e,h” | We have checked and revised it. |
| L115, please change “Figure 1f-g” to “Figure 1f,g” | We have checked and revised it. |
| L120, please change “Figure 1j-k” to “Figure 1j,k” | We have checked and revised it. |
| L126, please change “aspect ratio (AR)” to “AR” | We have checked and revised it. |
| L131, please set “x” and “z” in italics. | We have checked and revised it. |
| L132, 133, and in all other instances, please set single-letter variables “L”, “N” and “d” in italics. | We have checked and revised it. |
| L135,136, and in all other instances, please set single-letter variables “W”, “M” and “l” in italics. | We have checked and revised it. |
| L139, 140, 143, and in all other instances, please set single-letter variables “d”, “z”, “q” and “d” in italics. Please set numerical constant “π” in non-italics. | We have checked and revised it. |
| L146, please change “/2L” to “/(2L)” | We have checked and revised it. |
| L143, please change “Figure S10c-d” to “Supplementary Fig. 10c,d” | We have checked and revised it. |
| L145, please clarify “=” | We have revised the expression:The interdigitation factor Ƞ can be defined as Ƞ = (dz/(2L))*100%, and is usually in the 77-80% range. |
| L146, please change “77-80%” to “77%-80%” | We have checked and revised it. |
| L182, 183, 185, 186, please set “P”, “I” in italics. | We have checked and revised it. |
| L182, please change “polarization (P) equal” to “polarization equal” | We have checked and revised it. |
| L182, please clarify “similar” | We have replaced the word "similar" with consistent with for accurate description. |
| L189, please clarify “metallurgical” | The word "metallurgical" has been removed from the text. |
| L206, please clarify how the data was normalized. | Regarding the normalization method for the spectral data in Figure 4, we hereby provide a clear explanation: All data are processed using the maximum value normalization method. That is, for each spectral curve, the maximum signal intensity is set as 1, and the signal intensities of all other points on the curve are divided by this maximum value, thereby obtaining the normalized spectral data.  In the "Methods" section, we have provided detailed explanations regarding this data processing method. |
| L208, please ensure that section titles have 60 characters (including spaces) or fewer; for instance, please shorten “Circular dichroism and circularly polarized emission from 1D superlattices.” | According to the requirement, we have revised the section title to ensure its length (including spaces) is within 60 characters while maintaining full meaning. The revised title is: Chiral optical properties of 1D superlattices. Corresponding revisions have been updated in the manuscript. |
| L211, please change “Figure 4b-c” to “Figure 4b,c” | We have checked and revised it. |
| L214, please change “492 nm and 455 nm” to “492 and 455 nm” | We have checked and revised it. |
| L226, please change “328 nm to 235 nm and to 145 nm” to “328 to 235 and to 145 nm” | We have checked and revised it. |
| L227, please change “20 mg/mL to 10 mg/mL and to 5 mg/mL” to “20 to 10 and to 5 mg mL^-1” with “-1” as superscript. | We have checked and revised it. |
| L232, 233, 263, 264, 265, please set “θ”, “β”, “L” in italics. | We have checked and revised it. |
| L259, please change “Figure 5a-b” to “Figure 5a,b” | We have checked and revised it. |
| L263, please change “Figure 5a-b” to “Figure 5a,b” | We have checked and revised it. |
| L267, please change “Figure 5g-h” to “Figure 5g,h” | We have checked and revised it. |
| L269, please remove “(+)” and “(-)” | We have removed it. |
| L271, please change “Figure S20” to “Supplementary Fig. 20” | We have checked and revised it. |
| L275, please change “minute” to “min” when used as a unit in conjunction with a number. | We have checked and revised it. |
| L281, please change “Figure 5e-f” to “Figure 5e,f” | We have checked and revised it. |
| L288, please change “The corresponding video for the 3D structure is added as supplementary information (Video S1).” to “For the 3D structure, see Supplementary Movie 1”. | We have checked and revised it. |
| L290, please change “Figure 5k-l” to “Figure 5k,l” | We have checked and revised it. |
| L303, 308, 319, 331, 332, 339, 350, 352, please set “g”, “λ”, “n“, “L” in italics. | We have checked and revised it. |
| L312, please ensure that section titles have 60 characters (including spaces) or fewer; for instance, please shorten “Circularly polarized amplified spontaneous emission of 1D NPL superlattices.” | We have checked and revised it.CP-ASE of 1D NPL superlattices. |
| L316, please change “Figure 6a-b” to “Figure 6a,b” | We have checked and revised it. |
| L320, please change “µJ/cm^2” to “µJ cm^-2” | We have checked and revised it. |
| L324, please clarify “possibly” | We have removed it. |
| L309, please change “Fabry–Pérot (FP)” to “FP” | We have checked and revised it. |
| L327, please change “Fabry–Perot (FP)” to “Fabry–Pérot (FP)” | We have checked and revised it. |
| L341, please remove “Conclusion” | We have removed it. |
| L345, please set “P” in italics. | We have checked and revised it. |
| L360-362, please remove “This work was supported by the National Natural Science Foundation of China (Nos. 22305224 and 360 U24A2079), the China Postdoctoral Science Foundation (2022TQ0290) and the Ministry of Science 361 and Technology of China 343 (DL2023026004L).” | We have removed it. |
| L364, please add a section called “Funding” with the content “This work was supported by the National Natural Science Foundation of China (Nos. 22305224 and 360 U24A2079), the China Postdoctoral Science Foundation (2022TQ0290) and the Ministry of Science 361 and Technology of China 343 (DL2023026004L).” | We have checked and revised it. |
| L366, please change “draw” to “prepared” | We have revised it. |
| L366, please clarify “this experiment” | We have revised it. “this experiment” was revised to “this study”. |
| L367, please clarify “conducted experiment.” | We have revised it. “conducted experiment” was clarified as “conducted the structural and optical characterization experiments” |
| L368, please clarify “the experiment.” | We have revised it. “this experiment” was revised to “this lasing experiment”. |
| L368, please clarify “advised the manuscript” | We have revised it. “advised on the manuscript” was revised to “provided suggestions on the manuscript”. |
| L373, please remove “Supplementary information is available for this paper at https://doi.org/---------.” | We have removed it. |
| L375-377, please remove “Reprints and permissions information is available at <http://www.nature.com/reprints>. Publisher's note: Springer Nature remains neutral with regard to jurisdictional claims in published maps and institutional affiliations.” | We have removed it. |
|  |  |
| **Figures and Tables** |  |
| Our guidance: | Your response: |
| Please see the guidelines linked below for detailed instructions about how your figures should be prepared. Following these instructions will reduce the chances of delays should we need to request replacement artwork from you at a later stage. | We have revised all figures. |
| <https://www.nature.com/documents/NRJs-guide-to-preparing-final-artwork.pdf> |  |
| To ensure that your Figures are widely accessible, please make sure that the colour schemes used do not impair their readability. Please see the following resources for further information and suggestions:  Colour Brewer: http://colorbrewer2.org/#type=sequential&scheme=BuGn&n=3  Vischeck: http://www.vischeck.com/ Better Figures: https://betterfigures.org/2015/06/23/picking-a-colour-scale-for-scientific-graphics/ | We have revised all figures. |
| Using rainbow colour plots can be visually misleading (see links below) and I suggest altering the colour schemes used in your plots.  https://www.nature.com/articles/s41467-020-19160-7 https://www.climate-lab-book.ac.uk/2014/end-of-the-rainbow/ https://root.cern.ch/rainbow-color-map https://eagereyes.org/basics/rainbow-color-map | We have revised all figures. |
| Red/green colour contrasts can confuse our colour-blind readers; please consider recolouring such figures, if possible. | We have revised all figures. |
| Since your manuscript contains new cryoEM data, please include   - A cryo-EM data processing workflow including a representative micrograph as well as examples of the 2D-classes and major classes from 3D-reconstruction.   - The maps coloured to local resolution  - A global resolution estimate with FSC cut-off  - A representation of the angular distribution of particles used in the final reconstruction  - Maps for each helix shown individually with the structural model or, at the minimum, map and structural models for key structural elements  - Any substrate or ligand displayed as map fit with a stick model. Residues that form the binding site, and ligand, should be shown simultaneously at the same sigma level  - At least two viewing angles of all ligands with model-to-map fits  - A table summarizing structural and refinement statistics (see https://www.nature.com/authors/policies/tables-cryo-em.doc for a template)    This should be shown in one or multiple Supplementary Figures/Tables. | Our cryo-EM data have not undergone any processing, and the raw images are presented in the manuscript. |
| Please note that schemes are not used; sequences of chemical reactions or experimental procedures should be submitted as figures, with appropriate captions. | We have revised it. |
| All figures must include a legend/caption. These should be presented at the end of the article file, may be up to 350 words, and must refer to all panels. Figures must also be accompanied by a brief title that summarises the whole figure. | We have checked it. |
| Every figure panel must be described in the legend/caption. | We have checked it. |
| Please make sure that the terms ‘atomic units (a. u.)’, ‘absorbance units (abs. u.)’ or ‘arbitrary units (arb. u.)’ are appropriately used. Also, axis without numerical scaling and normalized data do not need specified units. | We have revised it. |
| Any abbreviations, symbols or colours present in your figures must be defined in the associated legends. | We have revised it. |
| All figures and tables must be cited in the main text, and numbered in the order in which they appear. | We have revised it. |
| In each Figure and Supplementary Figure where error bars are used, they must be defined. | We have revised it. |
| For all Figures and Suppl. Figures: We recommend using a consistent font and font size in all Figures and Figure panels; please revise. Please explain all colors used in the Figure in the Figure caption. | We have revised it. |
| For all data presented in a plot, chart or other visual representation, ensure that individual data points are shown when possible, and **always for n ≤ 10**. The format shows data distribution clearly (e.g. dot plots, box-and-whisker plots). Box-plot elements are defined (e.g. center line, median; box limits, upper and lower quartiles; whiskers, 1.5x interquartile range; points, outliers). Clearly defined error bars are present and what they represent (SD, SE, CI) is noted | We have checked it. |
| In Figures and Suppl. Figures, please use panel markers following the a, b, c, etc. convention, instead of (a), (b), (c), etc. Also, **please set panel markers in bold.** | We have revised it. |
| *When scaled to fit our article template, text and detail in some Figures may be too small to be legible. Please provide your final figures at article scale and as a guideline,* ***all text should be sans-serif typeface, preferably Helvetica or Arial. Maximum text size is 7 pt. Minimum text size is 5 pt.*** ***Please ensure a consistent font and font size throughout all Figures.***  For detailed guidance on figure preparation, see: https://www.nature.com/documents/NRJs-guide-to-preparing-final-artwork.pdf | We have revised it. |
| In Figures and Suppl. Figures, please use **sentence case**, i.e., in each text element, please capitalize the first letter only (with some exceptions, such as abbreviation and chemical notations). | We have revised it. |
| Scheme 1: We recommend using a consistent font and font size in all Figures and Figure panels; please revise. Please explain all colors used in the Figure in the Figure caption. Please change “Scheme” to “Figure”; also, the main text is missing a reference to this display item.  We discourage general references, such as “left’, “right”, “top”, “bottom”, when referring to content in panels with more than one plot; instead, please add an additional panel marker following the “a”, “b”, “c”, etc. convention to the bottom plot in 5d to facilitate referencing. Please use panel markers following the a, b, c, etc. convention. Also, please set panel markers in bold.  We discourage colored backgrounds, please remove the colored background. Please set all text elements in non-bold and in a consistent font size. Please clarify “Fluorine treated”. Please change “1D Superlattices” to “1D superlattices” and “Helical Nanoribbon” to “Helical nanoribbons”. Please ensure that all text elements in plots, such as axis labels, numerical axis markers, and inset text elements, are set in a consistent font size; as well as in non-bold and non-italics. Please set “CPL”, “CD”, “LPL”, “ASE” in non-italics and non-bold. Please clarify “LPL” and “ASE”. The plots are missing y-axis labels, e.g., “Intensity”. In the polar plot, please remove the colored background. Please remove the black frame around the Figure. | We have revised it accordingly. |
| Fig. 1: We recommend using a consistent font and font size in all Figures and Figure panels; please revise. Please explain all colors used in the Figure in the Figure caption.  Please use panel markers following the a, b, c, etc. convention, instead of (a), (b), (c), etc. Also, please set panel markers in bold.  In 1cko, please set “x” and “z” in sans-serif font. In 1dhlp, the plots are missing y-axis labels. In 1dhlp, please set bars in a solid color instead of gradual colors. In 1dhlp, please specify the number of independent samples/measurements. In 1a-p, please set all text elements in non-bold and in a consistent font size. | We have revised it accordingly. |
| Fig. 2: We recommend using a consistent font and font size in all Figures and Figure panels; please revise. Please explain all colors used in the Figure in the Figure caption.  Please use panel markers following the a, b, c, etc. convention, instead of (a), (b), (c), etc. Also, please set panel markers in bold. In 2d, please remove “Time-resolved In-situ Formation of 1D Superlattices”; this information should be provided in the Figure caption instead. In 2abcd, please set all text elements in non-bold and in a consistent font size. In 2d, please improve the visibility of the small black arrows in the right part of the schematic depiction. In 2de, please change “globally-aligned” to “globally aligned” and “globally-aligned” to “globally aligned”. In 2b, please set the scale bar text in non-bold. | We have revised it accordingly. |
| Fig. 3: We recommend using a consistent font and font size in all Figures and Figure panels; please revise. Please explain all colors used in the Figure in the Figure caption.  Please use panel markers following the a, b, c, etc. convention, instead of (a), (b), (c), etc. Also, please set panel markers in bold and black.  We discourage colored backgrounds in plots; in 3abcdefgh, please remove the colored backgrounds. In 3a-h, the plots are missing y-axis labels. In 3a-i, please set all text elements in non-bold and in a consistent font size. In 3a-h, please set the blue text in black and clarify its meaning, e.g., change “0.29” to “P=0.29” with “P” in italics. In 3a-h, please set the colored arrows in gray or black. In 3a-h, please remove the dashed frames around the panels. In 3i, please “Polarization” to “Degree of polarization, P” with “P” in italics. In 3i, please set all text elements in a consistent font size. Please change “:ref-#” to “: [#]”. | We have revised it accordingly. |
| Fig. 4: We recommend using a consistent font and font size in all Figures and Figure panels; please revise. Please explain all colors used in the Figure in the Figure caption.  Please use panel markers following the a, b, c, etc. convention, instead of (a), (b), (c), etc. Also, please set panel markers in bold and black.  In 4b, please change “Normalized Absorbance (a.u.)” to “Normalized absorbance”. In 4c, please change “Normalized intensity (a.u.)” to “Normalized intensity”, since a normalized axis does not need units to be defined. In 4d, please change “Normalized CD (mdeg)” to “Normalized CD”. In 4d, please change “Normalized CPL (mdeg)” to “Normalized CPL”. In 4bcde, the colors are hard to tell apart please use either different colors or use different lines styles, e.g. dashed or dotted to make the different data lines easier to distinguish. In 4a-e, please set all text elements in a consistent font size and in non-bold. | We have revised it accordingly. |
| Fig. 5: We recommend using a consistent font and font size in all Figures and Figure panels; please revise. Please explain all colors used in the Figure in the Figure caption.  Please use panel markers following the a, b, c, etc. convention, instead of (a), (b), (c), etc. Also, please set panel markers in bold and black.  In 5a-h, please set all text elements in a consistent font size and in non-bold. In 5gh, the images are missing a scale bar. In 5cdef, please set bars in a solid color instead of gradual colors. In 5cdef, please set blue text in black. In 5cdef, please change “Experimental numbers” to “Experiment”. In 5c-f, please change “Results” to “Sign of CD”. We recommend changing “+1” to “+” and “-1” to “-”. In 5i-l, the images are missing scale bars. In 5i-l, the images are missing a color scale. In 5i-l, we are discouraging the use of red-green contrasts. | We have revised it accordingly. |
| Fig. 6: We recommend using a consistent font and font size in all Figures and Figure panels; please revise. Please explain all colors used in the Figure in the Figure caption.  Please use panel markers following the a, b, c, etc. convention, instead of (a), (b), (c), etc. Also, please set panel markers in bold and black. In 6, please remove the dashed frames around the panels. We strongly discourage rainbow color schemes because they are perceptually non-uniform. In 6abi, please use a different color scheme – other than rainbow, preferably one that is perceptually uniform. See our guidance above for more information. In 6ab, please remove the blue background. In 6ab, please set “x”, “y” and “z” in italics. In 6ab, the color scales are missing a label and a clarification of the units. In 6abi, the images are missing a scale bar. In 6a-i, please set all text elements in a consistent font size and in non-bold. In 6d, please set “g” in italics. In 6ef, please remove “(a.u.)”. In 6ef, please set “σ”, “+” and “-” in sans-serif font. In 6gh, please change "~" to "≈". The polar plots are missing ac clarification of the radius and please clarify why the inner part of the polar plots are white and missing gridlines. In 6i, please set all text elements in non-bold and in a consistent font size, this also applies to the numerical markers along color scales. | We have revised it accordingly.  In panels a, b, and i, we have added scale bars. We have also added labels for the color scales and a clarification of the units: The color bar represents the electric field strength \|E\|, with the unit of V/m, scaled by ×10⁸.  Regarding “”inner part of the polar plots are white and missing gridlines. We have now revised the polar plots accordingly. To clarify the meaning of the radial axis, we have added the following explanation in the figure caption: The radial axis represents the relative ASE intensity, and the angular axis represents the polarization angle. |
| In Supplementary Figures, please use panel markers following the a, b, c, etc. convention, instead of (a), (b), (c), etc. Also, please set panel markers in bold and black. | We have revised it. |
| Suppl. Fig. 1bdf, 2e, 10cd, please specify the number of independent samples/measurements for each histogram in the Figure caption. | We have added it. |
| Suppl. Fig. 3, 6abc, 10ab, 13cd: Please remove “(a.u.)” | We have revised it. |
| Suppl. Fig. 3, please change “Theta” to “θ” in italics. | We have revised it. |
| Suppl. Fig. 3b, please clarify the meaning of colors. | We have added it. |
| Suppl. Fig. 10, please set “Q” in italics. | We have revised it. |
| Suppl. Fig. 10ab, please change “Log.Intensity” to “Log(Intensity)” | We have revised it. |
| Suppl. Fig. 11cd, please remove the colored backgrounds. | We have removed it. |
| Suppl. Fig. 11cd, the radius axis is missing a label. | We have added it. |
| Suppl. Fig. 13, please change “Normalized ABS” to “Normalized absorbance” or “Normalized Abs.” | We have revised it. |
| Suppl. Fig. 14 and in all other instances, please change “mg/mL” to “mg mL^-1” with “-1” as superscript. | We have revised it. |
| Suppl. Fig. 14g, 23, please change “(a.u.)” to “(arb.u.)” | We have revised it. |
| Suppl. Fig. 14i, 16, 17, 18, please set “g” in italics. | We have revised it. |
| Suppl. Fig. 16, 17, please change “ABS(a.u.)” to “Abs. (arb.u.)” | We have revised it. |
| Suppl. Table 1: please set “g” and “P” in italics. | We have revised it. |
| Suppl. Fig. 20, 21: please change “Results” to “Sign of CD”. We recommend changing “+1” to “+” and “-1” to “-”. Please change “Experimental numbers” to “Experiment”. | We have revised it. |
| Suppl. Fig. 22, please ensure that images are each accompanied by a scale bar. | We have added it. |
| L275, please remove “Video S1. 3D reconstruction video of the superlattices obtained by electron tomography.”.  Please include a 'Description of Additional Supplementary Files' file with your supplementary movie legends; please format the legends as follows:  File Name: Supplementary Movie 1  Description:  - Please note we need a separate PDF file with your supplementary movie legends. | We have removed it. |
|  |  |
| **Data and Code** |  |
| Our guidance: | Your response: |
| Nature journals strongly support public availability of data and code. Please deposit the data and code used in your paper into a public data repository, or alternatively, present the data as Supplementary Information. If data can only be shared on request, please explain why in your Data Availability Statement, and also in the correspondence with your editor.   Please note that for some data types, deposition in a public repository is mandatory. Any restrictions on sharing of these data types must be clearly indicated in the statement and discussed with the editor. More information on our data deposition policies and available repositories can be found here: |  |
| [https://www.nature.com/nature-research/editorial-policies/reporting-standards#availability-of-data](https://www.nature.com/nature-research/editorial-policies/reporting-standards#availability-of-data) |  |
| All published manuscripts reporting original research in Nature Portfolio journals must include a data availability statement, within the Methods and under the heading 'Data Availability'.     The data availability statement must make the conditions of access to the “minimum dataset” that are necessary to interpret, verify and extend the research in the article, transparent to readers. We ask that you don’t use phrases like ‘available on reasonable request’ but instead specify any restrictions to accessing your data as described below.    This minimum dataset may be provided through deposition in public community/discipline-specific repositories, custom proprietary repositories or general repositories like Figshare, Zenodo and Dryad. Providing large datasets in supplementary information is strongly discouraged and the preferred approach is to make data available in repositories. Please see https://www.springernature.com/gp/authors/research-data-policy/recommended-repositories for a list of recommended repositories.    If DOIs are provided, we also strongly encourage including these in the Reference list (authors, title, publisher (repository name), identifier, year).    The Data Availability Statement should also reference any source data published alongside the paper.    For clinical datasets or third party data, please ensure that the Data Availability statement adheres to our policy (https://www.nature.com/nature-research/editorial-policies/reporting-standards#availability-of-data)    If data are unavailable, please indicate the exact reasons why data cannot be made available in a suitable public repository or upon request, including any conditions related to ethical approval, consent from study subjects, commercial or legal restrictions, etc.    For data that are available under restricted access, the Data Availability statement must specify  - the reasons for access restrictions  - what the restrictions are   - how one can get access to the data  - who to contact to request access  - any restrictions on who the data can be made available to or for which purpose  - the expected timeframe for response to access requests  - for how long the data will be available once access has been granted. | The experimental data generated in this study are available under restricted access for non-commercial academic research only.  Access restrictions are imposed to prevent unauthorized commercial utilization of the raw and processed data.  Access to the restricted data can be obtained by sending a formal application email to the co-first author Kexin Chen (E-mail: 15515176996@163.com).  Data access requests are only open to academic researchers for non-commercial scientific research purposes.  All request emails will be responded to within one week after receipt.  Once access is granted, the shared data will be valid for one week only.  No public repository is used for data deposition in this work. |
| Please use the following template to provide all the information stated above:  The XX data generated in this study have been deposited in the YY database under accession code ZZ [add hyperlink here]. The XX data are available under restricted access for {insert reason}, access can be obtained by {explain how}. The raw XX data are protected and are not available due to data privacy laws. The processed XX data are available at YY. The XX data generated in this study are provided in the Supplementary Information/Source Data file. The XX data used in this study are available in the YY database under accession code ZZ [Add hyperlink here]. | The experimental data are available under restricted access for non-commercial academic research and prevention of unauthorized commercial exploitation, access can be obtained by contacting the first author via email for formal application. |
| In the Data Availability statement, please change "All the data supporting this study can be available in the article and the Supplementary Information." to "The data that support the findings of this study are available from the corresponding authors upon request. Unprocessed raw data are provided as Supplementary Data #." | We have revised it. |
|  |  |
| **Methods** |  |
| Our guidance: | Your response: |
| Sufficient details of the experiments must be provided in the Methods section such that they could be reproduced without reference to published papers. **Use of the term "as described previously" (or similar wording) is not encouraged.** | We have revised it. |
| Please abbreviate minutes as "min", hours as "h", milli liters as "mL", seconds as "s". | We have revised it. |
| Centrifugation speeds must be reported in “× *g*”, e.g., “5,000× *g”* with “g” in italics. | We have revised it. |
| Please specify molecular weights in either “g mol^-1” or “kg mol^-1” (instead of Da and kDa). For instance, please change “40000 MW” to “M_w 40,000 g mol^-1” or “M_w 40 kg mol^-1”. Also please set M_w or M_n, with “M” in italics and “w” and “n” in roman. | We have revised it. |
| In the Materials and Reagents section, please add information on the purity of all reagents used, for instance in % as stated by the supplier. | We have revised it. |
| **We advise against a fragmentation in experimental descriptions between the main article and the Suppl. Information file.**  **Experimental details provided in the Supplementary Information should be included in the Methods section in the main article, in a concise version giving all necessary details.** We encourage the text section in the Supplementary Information to be transferred to the Methods section of the main article, as we consider this information essential for publication. This includes p. 2-5.  Please note that there is no page or word limit for the main article file.  **While we do not impose a hard limit for the length and word count in the Methods section, we recommend rephrasing the text, as much as possible, to make it more concise without omitting important information.** | We have checked it. |
| The following lines and pages refer to the SI file: |  |
| P1, please remove “Vedio S1” | We have removed it. |
| P1, please change “Figures. S1 to S23” to “Supplementary Fig. 1-23” | We have revised it. |
| P1, please change “Table S1” to “Supplementary Table 1” | We have revised it. |
| P1, please change “References” to “Supplementary References” | We have revised it. |
| L23, please change “Materials and Methods” to “Methods” | We have revised it. |
| L24, please change “Chemicals / Materials” to “Materials” | We have revised it. |
| L27, please change “atom. %D” to “at.% D” | We have revised it. |
| L33, please clarify “General Reagents” | General Reagents is a reagent company, and we have revised its name to General Reagents Co., Ltd. |
| L36, please remove “Synthesis of CsPbBr3 nanorods” | We have removed it. |
| L37, please remove “(a)” | We have removed it. |
| L51, please remove “(b)” | We have removed it. |
| L50, please remove “Synthesis of CsPbBr3 nanoplatelets” | We have removed it. |
| L42, 43, 56, 57, please change “115°C” to “115 °C”, “30°C” to “30 °C”, and “40°C” to “40 °C” | We have revised it. |
| L45, please change “For” to “note: for” | We have revised it. |
| L48, 60, 66, 77, centrifugation speeds must be reported in “× g”, e.g., “5,000× g” with “g” in italics. | We have revised it. |
| L62, please remove “(c)” | We have removed it. |
| L65, 76, please change “minute” to “min” | We have revised it. |
| L67, 77, please change “minutes” to “min” | We have revised it. |
| L72, please remove “(d)” | We have removed it. |
| L84, please change “Figure S#” to “Supplementary Fig. #” | We have revised it. |
| L84, and in all other instances, please change “petri” to “Petri” | We have revised it. |
| L92, please change “Ultraviolet-Visible (UV-Vis) absorption measurements” to “UV-Vis absorption measurements” | We have revised it. |
| L97, please change “Photoluminescence (PL) and photoluminescence quantum yield (PLQY) measurements” to “PL and PLQY measurements” | We have revised it. |
| L101, please change “Circular dichroism (CD) and circularly polarized luminescence (CPL) measurements” to “CD and CPL measurements” | We have revised it. |
| L112, please change “X-ray Diffraction (XRD) measurements” to “XRD measurements” | We have revised it. |
| L125, please change “Small-angle X-ray scattering (SAXS) measurements” to “SAXS measurements” | We have revised it. |
| L129, please change “Optical microscope (OM) measurements” to “Optical microscopy” | We have revised it. |
| L132, please change “Transmission electron microscopy (TEM) measurements” to “Transmission electron microscopy” | We have revised it. |
| L138, please change “Scanning electron microscopy (SEM) measurements” to “Scanning electron microscopy” | We have revised it. |
| L143, please change “Nuclear Magnetic Resonance (NMR) measurements” to “NMR measurements” | We have revised it. |
| L147, please change “Time-resolved cryogenic transmission electron microscopy (Cryo-TEM) analysis” to “Time-resolved Cryo-TEM analysis” | We have revised it. |
| L156, please change “Electron tomography (ET) measurements” to “Electron tomography” | We have revised it. |
| L163, please change “Electromagnetic simulation of CPL” to “Electromagnetic simulations” | We have revised it. |
| L98, please change “photoluminescence (PL)” to “PL” | We have revised it. |
| L114, please change “Ka” to “K_α” with “K” in italics and “α” as subscript. | We have revised it. |
| L127, please set “θ” in italics. | We have revised it. |
| L130, please change “OM” to “Optical microscopy” | We have revised it. |
| L134, please change “carbon coated” to “carbon-coated” | We have revised it. |
| L151, please change “0 min, 30 min, 60 min, 90 min, 120 min and 150 min,” to “0, 30, 60, 90, 120, and 150 min,” | We have revised it. |
| L166, please set “z” in italics. | We have revised it. |
| In the SI, please set single-letter variable “d” in italics. | We have revised it. |
| In the SI, please change “mg/mL” to “mg mL^-1” with “-1” as superscript. | We have revised it. |
| In the SI, please use "≈" to signify “approximately” (instead of "~"). Please use “~” to signify “on the order of”. | We have revised it. |
|  |  |
| **References** |  |
| Our guidance: | Your response: |
| All references must be cited in numerical order. The reference list will be formatted according to the Nature style by our journal production team, however please ensure that references contain all of the information required, eg:  Kurumada, S., Takamori, S. & Yamashita, M. An alkyl-substituted aluminium anion with strong basicity and nucleophilicity. <i>Nat. Chem.</i> <b>12</b>, 36–39 (2020). | We have checked it. |
| Supplementary References should appear at the end of the Supplementary Information file, and **must be self-contained** and numbered from 1. References mentioned in both the main text and the Supplementary Information should be part of both reference lists so that the Supplementary Information does not refer to the reference list in the main paper and vice versa. | We have checked it. |
| In the Supplementary Information file, please rename "References" to "Supplementary references". | We have revised it. |
| In the References and Suppl. References, please give journal names in abbreviations following the CASSI guidelines (https://cassi.cas.org/search.jsp), e.g., “J. Catal.” instead of “Journal of Catalysis”. | We have revised it. |
| Please ensure that the **References and Suppl. References use the standard Nature referencing style**. All authors should be included in reference lists unless there are six or more, in which case only the first author should be given, followed by ‘et al.’. Authors should be listed last name first, followed by a comma and initials (followed by full stops, '.') of given names. Article titles should be in Roman text; only the first word of the title should have an initial capital, and the title should be written exactly as it appears in the work cited, ending with a full stop. Book titles should be given in italics and all words in the title should have initial capitals. Journal names are italicized and abbreviated (with full stops) according to common usage. Volume numbers and the subsequent comma appear in bold. The full-page range should be given where appropriate. Published conference abstracts, numbered patents, and archived code with an assigned DOI may be included in the reference list. | We have checked it. |
|  |  |
| **End matter** |  |
| Our guidance: | Your response: |
| Please supply an "Author Contributions" section after the "Acknowledgements" section that refers to all authors. For more information on the Author Contributions statement, please refer to our authorship policy(https://www.nature.com/nature-research/editorial-policies/authorship), and to the following Nature Editorial: https://www.nature.com/articles/4581078a. | We have added a complete section on author contributions, clearly indicating the specific contributions of each author. |
| Nature Portfolio defines Competing Interest (CI) as financial and non-financial interests (including but not limited to funding, employment, stocks, shares, patents, personal or professional relationships with individuals or institutions, and unpaid membership advocacy) that could be perceived to directly undermine the objectivity, integrity, and value of a publication, or could be seen as having an influence on the judgments and actions of authors with regard to objective data presentation, analysis, and interpretation.  Please thoroughly review our policy on Competing Interests and include a detailed statement both in your final manuscript file and in our manuscript tracking system. Please ensure the statements are identical in both. Be specific about how each point stated relates to the research and list applicable author initials, and/or patent numbers.  If there are no competing interests, a negative statement must be included. | We declare no competing interests. |
| <https://www.nature.com/nature-research/editorial-policies/competing-interests> |  |
| Any relevant funding should be declared in a separate funding statement. Please refer to our funding statement guidelines (https://www.nature.com/nature-portfolio/editorial-policies/funding) for more information. | We have provided the required funding statement (Funding Statement), indicating all relevant funding projects and their corresponding numbers. |
|  |  |
|  |  |
| **Preparing your manuscript files** |  |
| Our guidance: | Your response: |
| Unless otherwise stated please limit individual file sizes to approximately **20 MB**. We strongly encourage the use of repositories for large datasets or source data due to size considerations. | All requirements will be fully fulfilled. |
| Please supply a brief (**maximum 250 characters {not words}, including spaces**) summary of the main findings of the paper to be used on our website and in our e-alerts. The summary should be written in the third person in language suitable for a broad audience. It should follow the structure: "[[Brief background first sentence]]. Here, the authors [[brief description of accomplishment]]." The summary may be edited by the editors prior to publication. Please provide this summary here. | All requirements will be fully fulfilled. |
| To ensure maximum visibility for your work, we may tweet about your paper following publication. If you would like us to include the **X** (formerly **Twitter**) and/or **Bluesky** **handles** of the first author(s), corresponding author(s), lab, or institution in this tweet, please provide them here. We would also welcome your suggestions for a sentence (**max. 150 characters {not words}**) or hashtags to use when tweeting about the work. You are invited to submit **artistic renderings** of your work for the tweet. | All requirements will be fully fulfilled. |
| Large datasets exceeding an A4 page size should be supplied as Supplementary Data files to allow reuse, not Supplementary Tables. | All requirements will be fully fulfilled. |
| **Please provide figures as individual vector files with editable text. Acceptable file types for figures are .ai, .eps, .pdf, .ppt, .pptx, or Chem Draw for fully editable vector-based art. For detailed guidance on figure preparation, see *https://www.nature.com/documents/NRJs-guide-to-preparing-final-artwork.pdf*** | We have provided all figures as editable files in PPT format. |
| Please note that all Supplementary Information must be provided as a single separate PDF file and single separate DOCX file, not within the manuscript file.  All Supplementary Information items (e.g., Supplementary Figures, Supplementary Tables, Supplementary Methods, Supplementary Notes, Supplementary Discussion, Supplementary References) must be included in one PDF document. Please refer to our formatting guide when preparing your supplementary information file: https://www.nature.com/documents/ncomms-formatting-instructions.pdf    All Supplementary Information files (e.g. Supplementary Data, Supplementary Software, etc.) must be cited in the main text.  **Every Supplementary Figure must be accompanied by a legend of up to 350 words, referring to all panels, and a brief title that summarises the whole figure.**  Only Supplementary Movie, Audio, Data and Software files should be submitted separately from the Supplementary Information. | All requirements will be fully fulfilled. |
| Please supply legends for each Supplementary Movie/Audio/Data file in your response here (not in the Supplementary Information file). Please label each files as Supplementary Movie/Audio/Data 1, etc. | All requirements will be fully fulfilled. |
| The use or adaptation of previously published images is strongly discouraged. If this is unavoidable, please request the necessary rights documentation to re-use such material from the relevant copyright holders and return this to us when you submit your revised manuscript. Please check whether your manuscript or Supplementary Information contain third-party images, such as figures from the literature, stock photos, clip art or commercial satellite and map data. Please note that Springer Nature journals do not permit the use of generative AI in our publications. For concessions and additional details relating to this policy, please refer to the following page on Artificial Intelligence: https://www.nature.com/nature-portfolio/editorial-policies/ai  If any elements of your submitted work have been created with BioRender you will need to ensure you have obtained a publication license from BioRender, adhering to the user requirements as outlined within the license. The reference for BioRender created graphics should be present in the accompanying legend of the display material it is present in.  A copy of the publication license should be uploaded to our system as a related manuscript file upon resubmission.  For more information please see the BioRender knowledge article here: https://help.biorender.com/hc/en-gb/articles/21283116932765-CC-BY-publishing-and-reader-permissions  For more information on what constitutes ownership by a third party, please contact our Editorial Assistant at naturecommunications@nature.com | We have carefully checked the manuscript and supplementary materials.All images, figures and illustrations in this submission are originally created by us.No third-party copyrighted materials, stock photos, clip art, commercial map/satellite data or contents generated by generative AI are used in this paper.No BioRender figures are included.We confirm full compliance with the copyright and AI policy of Springer Nature. |
|  |  |
| **Please note that suspected third party content is present in Figures 2d; Scheme. 1 and Supplementary Figure 5.** | We have carefully checked Figure 2d, Scheme 1 and Supplementary Figure 5.We confirm that all of these figures are completely original and created by the authors, without any third-party content or copyrighted material.No unauthorized cited or copied content was involved in this work. |
|  |  |
| **Forms to complete** |  |
| Our guidance: | Your response: |
|  |  |
|  |  |
|  |  |
|  |  |
|  |  |
| **You will need to upload:** |  |
| Completed Third Party Rights Table (if relevant) |  |
| A point-by-point response to the reviewers' comments | We have provided the corresponding file. |
| A completed copy of this checklist in .docx format | We have provided the corresponding file. |
| The main manuscript file in either Microsoft Word or LaTeX format | We have provided the corresponding file. |
| Separate Figure files (one file per figure) | We have provided the corresponding file. |
| Source Data file (.xslx file or .zip folder) | We have provided the corresponding file. |
| Inventory of Supporting Information in .docx format | We have provided the corresponding file. |
| A Supplementary Information file in .pdf format | We have provided the corresponding file. |
