# Supplementary material for: One-dimensional CsPbBr3 superlattices with polarized and amplified spontaneous circularly polarized emissions
Source: Nat Commun. 2026 May 23;17:6768. doi: 10.1038/s41467-026-73513-2 (PMC13385850; doi:10.1038/s41467-026-73513-2)
Supplement: Supplementary file 6 — Transparent Peer Review file [file 41467_2026_73513_MOESM6_ESM.pdf]

nature portfolio

Peer Review File

One-dimensional $CsPbBr_3$ nanocrystal superlattices with polarized and amplified spontaneous circularly polarized emissions

Corresponding Author: Professor Siyu Lu

This file contains all reviewer reports in order by version, followed by all author rebuttals in order by version.

Version 0:

Reviewer comments:

Reviewer #1

(Remarks to the Author)
The authors report CPL in their NPL self-assembled structures. Overall I do not find the current form of manuscript merits publication in Nat. Commun. More detailed analysis and understanding are required to elucidate the fundamental mechanisms resulting in CPL. My details questions are as follows.

1. I cannot find any TEM evidence that the synthesized nanocrystals are NPLs. They can also be nanorods, since there is a luck of horizontally oriented NPLs captured in the TEM images.

2. It is not clear to me how the authors prepare the 1D superlattice samples (Fig. 2c-2h). How do the authors align the fibrillar-like assemblies? What are the driving force of alignment? How did the authors increase the degree of linear polarization? Is the polarization in parallel to the long axis of fibrillar-like assemblies?

3. There is no direct evidence showing the NPLs are stacked and gradually rotates along the long axis of fibrillar structure. More detailed cross-sectional TEM using FIB may help to resolve the structure.

4. What are the physical mechanisms and driving force for the NPLs to stack with gradual rotational variation?

5. Even the NPLs are stacked according to Fig. 4g and 4h, detailed electromagnetic simulations are required to show CPL can be generated.

6. The authors report enhanced g_lum in their ASE without detailed understanding. The authors only cited a few reference and suggested the cavity effects. Please carry out detailed optical simulations to support the claims. Also there are many samples presented in Fig. 2. Is there any correlation between the degree of linearly polarized emission vs CPL g_lum vs ASE g_lum? Please carefully and statistically characterized more samples to draw a conclusion.

7. It would be great if the authors can quantify the dipole orientation of CPL for the prepared films using k-space microscopy or angular resolved PL with linear polarizer.

Reviewer #2

(Remarks to the Author)
The authors reported on the self-assembly of CsPbBr3 nanoplatelets for the preparation of 1D superlattices of varying lengths. The nanoplatelets stack into ribbons while the shape of the nanoplatelets influences the aspect ratio of the superlattices. The superlattices exhibit strong polarized emission owing to the anisotropic shape. The superlattices stack into a chiral configuration along the length due to the orientational twist of these nanoplatelets. The chiral configuration of the superlattices results in circular dichroism and circularly polarized luminescence. The chirality is controlled after the addition of chiral ligands exchanging the pristine ligands. A moderate

dissymmetrical factor is obtained from the self-assemblies of the nanoplatelets. The helical assembly of the nanoplatelets and the chiroptical properties are interesting. I recommend publication of this paper in Nature Communications after the authors have considered and addressed the following comments:
Comments:
(i) The synthesis of the nanoplatelets were carried out using didodecylamine (DDDAm)
and oleic acid as surfactants. Whether the lateral spacing between the adjacent nanoplatelets has any correlation with the lengths of these ligands.
(ii) The length of the superlattices of the nanoplatelets was increased by reducing the thickness of the nanoplatelets. How the nanoplatelets are stacked into twisted configuration? The authors should comment on this.
(iii) When oleylamine was used in the synthesis, the thickness of the reduced to 4.6 nm and the lateral size became ~29 nm. The length of the ribbons became ~1000 µm with a width of ~0.8 µm. This observation implies that the ribbons consist of many nanoplatelets along the width and lenths of the ribbons. What is the orientation of the individual nanoplatelets inside the ribbon?
(iv) The individual $CsPbBr_3$ nanoplatelets are polarized owing to their anisotropic shapes, with the randomly oriented nanoplatelets show lesser degree of polarization compared to the ribbons. How the polarization was measured? Is there any correlation with the excitation polarizer and nanoplatelets orientation?
(v) The intrinsic chiral origin of $CsPbBr_3$ nanoplatelets is unclear. The distorted $PbBr_6$ octahedra may also incorporate intrinsic chirality in these halide perovskite nanocrystals. The authors should comment on this. Also the dissymmetrical factor (gabs) of CD may be useful.
(vi) Nanocrystals exhibiting circularly polarized luminescence are of great interest, yet the distinction of circularly polarized luminescence is not made clear in the text. What is the origin of circularly polarized luminescence in the $CsPbBr_3$ nanoplatelets?
(vii) Could the authors show film thickness- and orientation-dependent CD curves for the nanoplatelets?
(viii) The amplified spontaneous emission of 1D nanoplatelet superlattices is interesting, however, the description is too short. A detailed discussion may be useful.

Version 1:

Reviewer comments:

Reviewer #1

(Remarks to the Author)
The authors have carried out considerable amount of extra experiments to address my questions. Although they did not perform the k-space imaging to determine the dipole orientation, I find the new scientific evidence, including the new TEM characterization, tomography, and many new optical simulations are sufficient to justify the publication of this manuscript. This is a great piece of work. I support publication of the current form of manuscript.

Reviewer #2

(Remarks to the Author)
In the previous round of review, I found the helical self-assembly of the nanoplatelets and their chiroptical properties to be interesting. However, the new set of experiments and simulations raises additional questions, as outlined below. I recommend publication of this paper in Nature Communications after the authors have addressed the following comments.
Comments:
1. The assembly process starting from the initial droplet to dried fibrillar-like assemblies using cryo-TEM was demonstrated using NRs. However, the major focus of this work is the self-assembly of NPLs. It would be interesting to see the formation process of superlattices of NPLs.
2. To probe the NPLs stacking and gradual rotation along the long axis of fibrillar structure, 3D reconstruction based on scanning TEM and 3D FFT images are provided. However, it is difficult to observe rotations of the NPLs from these images.
3. Electromagnetic simulations were performed to show CPL from the stacked NPLs. The simulated CPL and g-factor do not contain units (figure R9), although the g-factor was compared with the experimental value (figure 3e). Also, CD and CPL plots in measured scale may be provided.
4. Optical simulations were performed to demonstrate the optical microcavities effect of 1D superlattices (Figure 6i). However, the mechanism of CPL g-factor vs ASE g-factor still remain unclear.
5. The interparticle spacing for the vertical direction was calculated using SAXS and spacing along the lateral direction was calculated using TEM. The interparticle spacings appear to be different for the vertical and lateral directions.
6. The authors stated that the stress induced by the ligands under locked condition led to the twisting of the NPLs. However, the interparticle distance between the NPLs depends on the thickness of the NPLs which in tern has an effect on the locking of the NPLs. Hence, the stress may not be the same for all the NPLs which will affect the degree of rotation. Additionally, the self-assembly of cadmium selenide nanoplatelets [Science Advances 2017, 3 (9), e1701483] did not show chirality in spite of ligand induced stress.
7. The polarization measurements were carried out with a polarizer behind the emission lens to control the polarization direction. This eventually gives the emission polarization. My specific query was with the polarization behavior with excitation polarizer and nanoplatelets orientation.
8. The dissymmetric factor obtained from CD is in the range of 10-3 to 10-4 while the dissymmetric factor from CPL appears to be ~ 10-2. Generally, the dissymmetric factor for CPL remains smaller than the dissymmetric factor of CD. Please

comment.

9. CsPbI3 NPLs (Nature Photonics, 2024, 18, 586– 594); CsPbBr3 NPLs (Nano Lett. 2023, 23, 2148-2157); CsPbBr3 NPLs (J. Am. Chem. Soc. 2015, 137, 16008-16011); CsPbBr3 NPLs (Light: Science & Applications (2025) 14:336); CsPbBr3 NPLs (Nano Lett. 2020, 20, 1808-1818); CsPbBr3 NPLs (J. Phys. Chem. Lett. 2019, 10, 1300-1305) exhibited similar self-assemblies of halide perovskite NPLs. Please include these references.

Open Access This Peer Review File is licensed under a Creative Commons Attribution 4.0 International License, which permits use, sharing, adaptation, distribution and reproduction in any medium or format, as long as you give appropriate credit to the original author(s) and the source, provide a link to the Creative Commons license, and indicate if changes were made.
In cases where reviewers are anonymous, credit should be given to 'Anonymous Referee' and the source.
The images or other third party material in this Peer Review File are included in the article's Creative Commons license, unless indicated otherwise in a credit line to the material. If material is not included in the article's Creative Commons license and your intended use is not permitted by statutory regulation or exceeds the permitted use, you will need to obtain permission directly from the copyright holder.
To view a copy of this license, visit https://creativecommons.org/licenses/by/4.0/

Reviewers' comments:

Reviewer #1 (Remarks to the Author):

The authors report CPL in their NPL self-assembled structures. Overall, I do not find the current form of manuscript merits publication in Nat. Commun. More detailed analysis and understanding are required to elucidate the fundamental mechanisms resulting in CPL. My details questions are as follows.

1. I cannot find any TEM evidence that the synthesized nanocrystals are NPLs. They can also be nanorods, since there is a luck of horizontally oriented NPLs captured in the TEM images.

Response: We thank the reviewer for this suggestion. To avoid edge-up stacking and obtain the horizontally oriented nanocrystals, we have prepared a diluted sample (1 mg/mL,0.5 mg/mL and 0.1 mg/mL) for TEM characterization. As shown in Figure R1, the TEM images of the non-stacked 'DDDAM-NPL' sample indicate that the particles have a shape that can be defined as a nanorod. Further evidence can be found in panel c of Figure R1 (see discussion below), where some of these rods are laying perpendicular to the grid. The rods have a square cross section with an edge length of around 8 nm, while their average length is around 42 nm. Hence, to reply to the comment of the reviewer and in analogy with previous morphological characterization on $CsPbBr_3$ nanorods (*J. Mater. Chem. C*, **2024,** *12,* 4234–4242; *Adv. Optical Mater.* **2024,** *12,* 2302129) we have indeed decided to rename this sample as NR. Instead, for the OAm-sample (Figure R2), the TEM images reveal a clear 2D nanoplatelet shape. The lateral length is ~25-35 nm, and the thickness is around ~4.6 nm (determined by images in which several platelets are edge-up stacked).

Overall, these new results demonstrate that the sample synthesized with DDAM is made of nanorods (NRs), while the sample synthesized with OAm is made of NPLs. Our experiments indicate that both the $CsPbBr_3$ NRs and the NPLs can assemble into ordered 1D superlattices. As shown below, we compare the aspect ratio, the polarized emission and the circularly polarized emission of the 1D superlattices assembled from the NRs and the NPLs (Table R1). Generally, the NPLs based superlattices exhibit larger aspect ratio, higher polarized emission and larger dissymmetry factor g_{lum} than the NRs based superlattices.

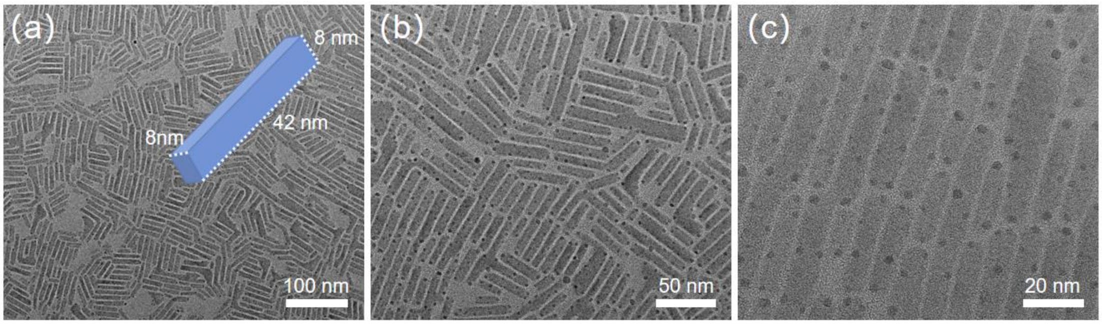

Figure R1. TEM characterization of the sample synthesized using DDDAm with concentrations of (a) 1 mg/mL, (b) 0.5 mg/mL and (c) 0.1 mg/mL, respectively. The images generally point to a nanorod (NR) shape. We rename this sample as NRs-DDDAm-1. The geometrical parameters for this sample are: edge of ~8 nm and length of ~42 nm [inset of panel (a)].

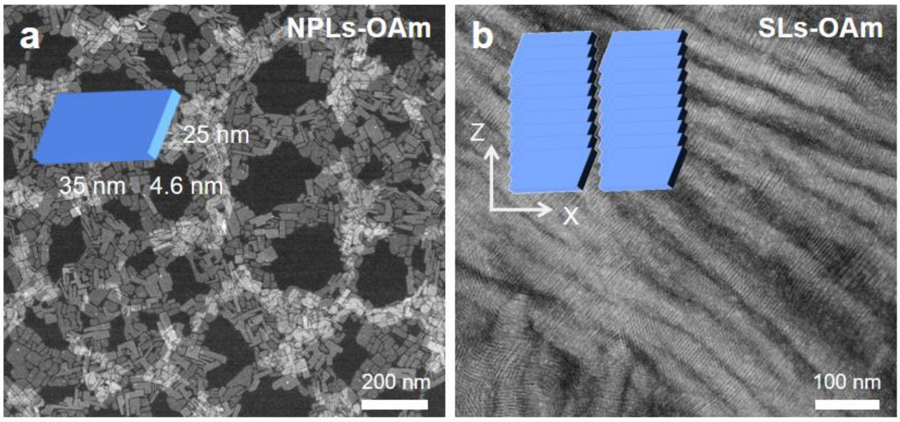

Figure R2. TEM images of (a) Edge-up stacked and (b) horizontally oriented NPLs-OAm. These indicate a NPLs shape for the OAm sample with a diameter of ~4.6 nm and lateral length of ~25-35 nm.

	Aspect ratio (AR)	Polarized emission (P)	Circularly polarized luminescence (│g_{lum}│)
SLs-NRs	5	0.72	0.02
SLs-NPLs	7.7	0.93	0.11

Table R1. Comparison of aspect ratio (AR), polarized emission (P), and circularly polarized luminescence (|g_{lum}|) between 1D superlattices assembled from $CsPbBr_3$ nanorods (SLs-NRs) and nanoplatelets (SLs-NPLs).

Action: We have updated Figures R1, R2 and Table R1 as Figure S2, Figure S7 and Table S1 in the Supporting Information. All the descriptions of 'Nanoplatelet' for the DDAM sample have been corrected as 'Nanorod' in this work. The following text has

been added to highlight the comparison between the NRs based 1D superlattices and NPLs based 1D superlattices:

'Both the $CsPbBr_3$ NRs and NPLs can assemble into ordered 1D superlattices. As shown in Table R1, the NPLs based 1D superlattices exhibit larger aspect ratio, higher polarized emission and larger dissymmetry factor (g_{lum}) than the NRs superlattices, possibly due to the stronger anisotropic shape of the NPLs.'

2. It is not clear to me how the authors prepare the 1D superlattice samples (Fig. 2c-2h). How do the authors align the fibrillar-like assemblies? What is the driving force of alignment? How did the authors increase the degree of linear polarization? Is the polarization in parallel to the long axis of fibrillar-like assemblies?

Response: We thank the reviewer for these comments.

2.1. The assembly protocols were described in the 'Materials and Methods' section in the Supporting Information. To better show the protocols, we have prepared the scheme reported below (Figure R3). Firstly, $CsPbBr_3$ NRs or NPLs were dispersed in a toluene solution at a concentration of 10 mg/mL. The glass slides were treated with perfluorodecyltriethoxysilane to make them hydrophobic, thus enhancing their interaction with the toluene solution. Subsequently, the treated glass slides were placed in a Petri dish, and 30 µL NRs/NPLs stock solution was drop-cast onto the slides using a pipette. The Petri dish was covered and wrapped with foil to avoid light and dust. Under these conditions, the solvent was allowed to evaporate slowly over several hours, after which the glass slides were removed for further characterization. It is worth to note that, if the Petri dish was left uncovered, the rapid solvent evaporation would result in the formation of randomly assembled NPLs/NRs, instead of 1D superlattices.

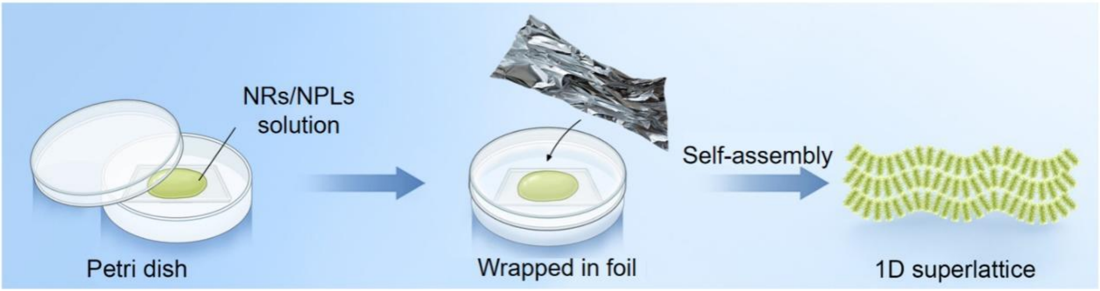

Figure R3. Sketch of the set-up for the assembly experiment. The treated glass slide is placed in a Petri dish. 30 uL of the NCs solution (20 mg/mL) are dropped onto the glass slide. The lid of the Petri dish is used to cover the glass slides, and the whole setup is wrapped in aluminum foil.

2.2-2.3. To investigate the assembly process starting from the initial droplet to

dried fibrillar-like assemblies, cryo-TEM at different times was carried out (Figure R4 below). In details, three copper net grids were placed on the substrate, and the NRs toluene solution (10 mg/mL) was drop-cast on each of the grids. Following the assembly process at different times, each grid was then rapidly plunge-frozen at different times (0 min, 90 min, 150 min) using liquid ethane. As shown in Figure R4a, the cryo-TEM images of the NRs sample at 0 min did not evidence any clear alignment globally, as the NRs mainly formed disordered clusters. At 90 min, the NRs started to organize into locally aligned zones (Figure R4b). At 150 min, globally aligned NRs were observed, corresponding to the 1D superlattices under the optical microscope (Figure 1a).

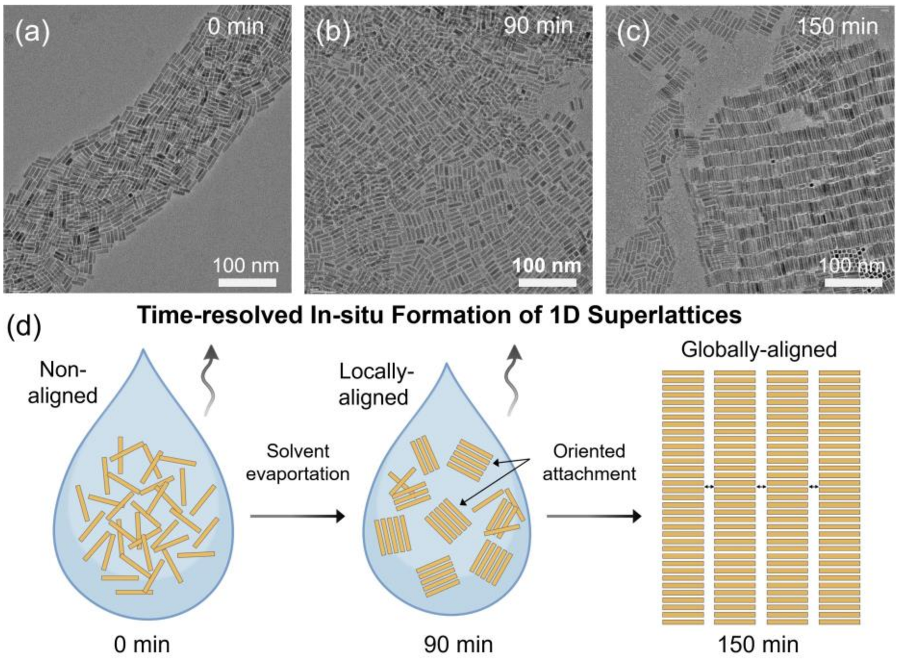

Figure R4. Monitoring the formation process of one-dimensional superlattices by cryo-TEM. (a-c) Cryo-TEM images during the process of solvent evaporation, for evaporation times of 0 min (a), 90 min (b) and 150 min (c), respectively. (d) Sketches of the assembly process during the evaporation, from non-aligned to locally-aligned and then to globally-aligned particles.

2.4. The increase in linear polarization is strongly related to the degree of order of the 1D superlattices. The randomly assembled NRs/NPLs were obtained by randomly evaporation (i.e. not following the confined evaporation procedure discussed above), while the 1D superlattices were obtained only under confined evaporation conditions (Figure R3). Optical microscopy images and polar plots of the normalized intensity of the photoluminescence emission as a function of detection angle for random assembled NRs and 1D superlattices are shown in Figure R5. The randomly assembled NRs sample exhibits no clear orientation (Figure R5a), corresponding to a weak polarized emission (P = 0.16, Figure R5c). In contrast, 1D superlattices sample shows strong orientation

(Figure R5b) and significantly stronger polarized emission (P = 0.72, Figure R5d).

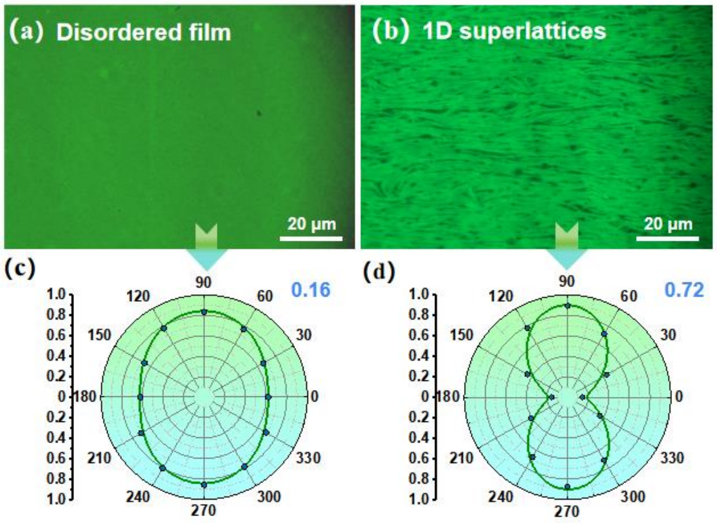

Figure R5. (a-b) Optical microscopy images of (a) a disordered NRs-DDDAm-1 film prepared by random evaporation assembly and (b) a 1D NRs-DDDAm-1 superlattices film prepared by confined evaporation; (c-d) Polar plots of the normalized intensity of the photoluminescence emission as a function of detection angle for (c) disordered NRs-DDDAm-1 film and (d) 1D NRs-DDDAm-1 superlattices film.

2.5. To analyze the polarized emission direction of the samples, we employed a simplified polarization system based on a RX50M upright metallurgical microscope. This system consists of a polarizer and an analyzer, where the detection angle can be tuned by rotating the analyzer (0–360°). As shown in Figure R6, the fluorescence intensity of the sample exhibits a trend of "increase-decrease-increase-decrease" by rotating the analyzer in steps of 90°. In particular, the weakest and strongest intensity appeared at 45° and 135°, respectively, which indicates that the polarization direction is in perpendicular to the long axis of the 1D superlattices, or parallel to the long axis of the NRs/NPLs. This indicates that the polarized emission stems from the building blocks (NPLs/NRs) of the 1D superlattices. This was already stated in the manuscript, (apart from the addition of "NR"): 'Due to their anisotropic shape, individual $CsPbBr_3$ NPLs/NRs are known to show polarized emission. A random assembly of NPLs on the other hand can partially average out this polarization. The ordered 1D superlattices of this work are instead built from oriented NPLs/NRs and are thus expected to preserve the polarized emission'.

The related phenomenon can be also found in *Nature Photonics,* **2024,** *18,* 586–594 and *Nano Letters,* **2014,** *14,* 710–715.

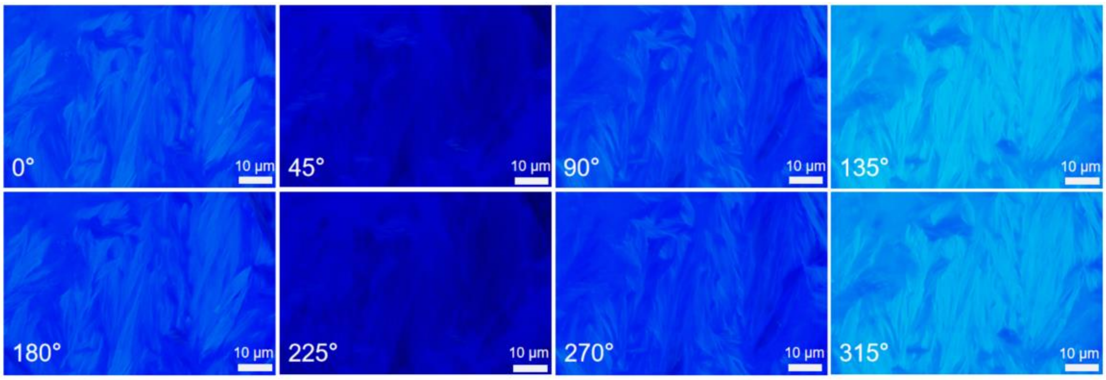

Figure R6. (a-h) Polarized fluorescence optical micrographs taken under crossed polarizers for film by rotating 1D SLs-OAm films by different angles from 0° to 315°. (The film was rotated in 45° increments from 0° to 315°.)

Action:

(1) To better describe the assembly protocols, the scheme (Figure R3) has been added as Figure S5 in the Supporting Information.

(2) To investigate the alignment process, we have added the cryo-TEM analysis as a new Figure 2 in the manuscript, and added the following paragraph:

'To investigate the assembly process starting from the initial droplet to dried fibrillar-like assemblies, cryo-TEM analysis at different times was carried out (Figure R4 below). In details, three copper net grids were placed on the substrate, and the NRs toluene solution (10 mg/mL) was drop-cast on each grid. Each grid was then rapidly plunge-frozen at different times (0 min, 90 min, 150 min) using liquid ethane. As shown in Figure R4a, the cryo-TEM images of the NRs sample at 0 min did not evidence any clear alignment globally, as the NRs mainly formed disordered clusters. At 90 min, the NRs started to organize into locally aligned zones (Figure R4b). At 150 min, globally aligned NRs were observed, in line with the corresponding 1D superlattices observed under the optical microscope (Figure 1a).'

(3) To study the correlation between the orientation of the superlattices and linear polarization, we have added the control experiment (Figure R5) and the orientation dependent polarized emission (Figure R6) as Figures S11 and Figure S12 in the Supporting Information. The following text has been added in the manuscript:

'To analyze the polarized emission direction of the samples, we employed a simplified polarization system based on a RX50M upright metallurgical microscope in this study. This system consists of a polarizer and an analyzer, where the detection angle can be tuned by rotating the rotatable analyzer (0 - 360°). As shown in Figure

S12, the fluorescence intensity of the sample exhibits a trend of “increase-decrease-increase-decrease” by rotating the analyzer in steps of 90°. In particular, the weakest

a n d s t r o n g e s t i n t e n s i t y a p p e a r e d a t 4 5 a n

We have cited the references as ref-17 and 42. (*Nature Photonics*, **2024**, *18*, 586–594 and *Nano Letters*, **2014**, *14*, 710–715.)

3. There is no direct evidence showing the NPLs are stacked and gradually rotates along the long axis of fibrillar structure. More detailed cross-sectional TEM using FIB may help to resolve the structure.

Response: We thank the reviewer for this suggestion. To directly investigate the stacking of NPLs in the superlattices (NPLs-OAm based 1D superlattices), we employed 3D reconstruction based on scanning TEM. As shown in Figure R7, four orthoslices (Figure R7b-e) of stacked NPLs have been collected and used to reconstruct a 3D image (R7a). The corresponding video for the 3D structure is added as supplementary information (Video R1) that support the rotated NPLs in the fibrillar structure. Accordingly, 3D FFT analysis of the stacked NPLs was carried out, as shown in Figure R8. The discrete dots (highlighted by the red arrows) indicate a distorted lamellar pattern between the stacked NPLs, in analogy with previous works (*Journal of Structural Biology*, **2012**, 178, 152–164; *Am. J. Phys,* **2018**, 86, 95–104). These results support our hypothesis that the NPLs are stacked along their long axis with different rotation angles relative to each other.

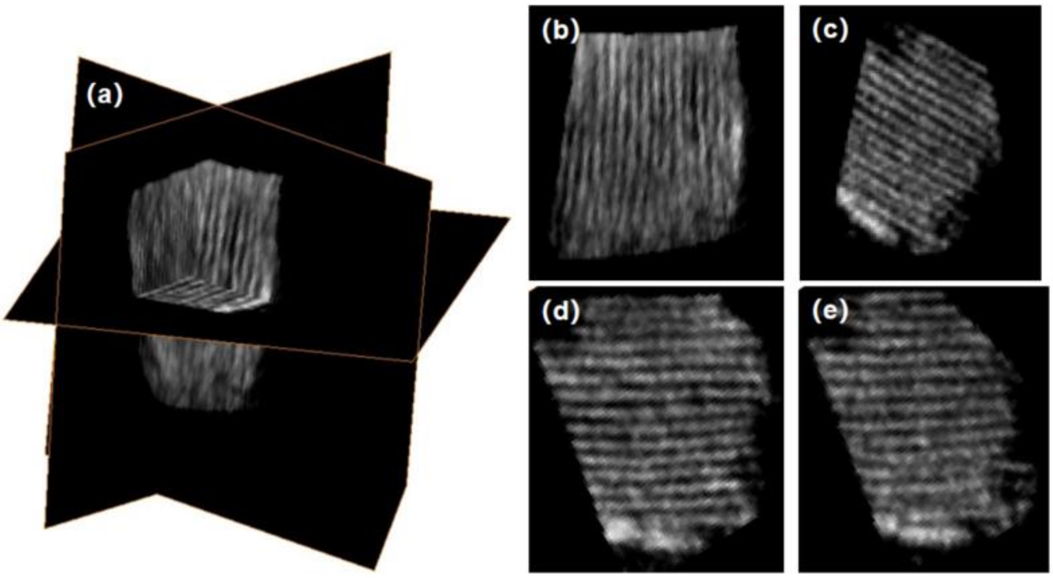

Figure R7. 3D reconstruction of the superlattices obtained by electron tomography, showing the reconstructed 3D volume and orthoslices from different projections.

Vedio 1.mp4

Video R1. 3D reconstruction video of the superlattices obtained by electron tomography.

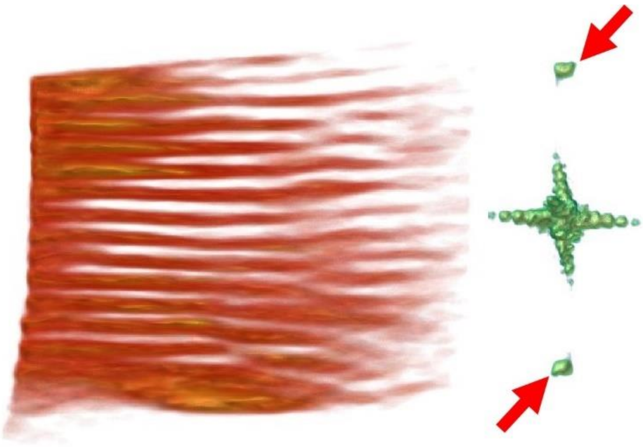

Figure R8. 3D reconstructed volume and 3D fast Fourier transform (FFT) analysis on the reconstructions of the stacked NPLs.

Action: We have added Figure R8 as Figure 5i-l in the manuscript, along with the following discussion:

'To directly investigate the stacking of NPLs in the superlattices (NPLs-OAm based 1D superlattice), we employed 3D reconstruction based on scanning TEM. As shown in Figure R7, four orthoslices (Figure R7b-e) of stacked NPLs were collected, and used to reconstruct a 3D image (Figure Ra). The corresponding video for the 3D structure is added as supplementary information (Video R1). 3D FFT analysis of the stacked NPLs was carried out, as shown in Figure 5k-l. The discrete dots (highlighted by the red arrows) indicate a distorted lamellar pattern between the stacked NPLs, in analogy with previous works. These results support our hypothesis that the NPLs are stacked along their long axis with different rotation angles relative to each other.'.

We have added Figure R7 and Video R1 in the Supporting Information. We have cited the references as ref-55 and 56. (*Journal of Structural Biology*, **2012**, *178,* 152–164; *Am. J. Phys*, **2018**, *86*, 95–104.) We cited these references to provide supporting evidence for the interpretation of the 3D FFT analysis results: We have added an explanation in the manuscript regarding citing these references.

We have added the sample preparation and testing methods for the electron tomography analysis the SI as follows:

‘Tomography series were acquired using an aberration corrected “cubed” Thermo Fisher Scientific Titan 60-300 electron microscope operated at 120 kV with a single-tilt tomography holder over a tilt range from −68° to +72°, with tilt increments of 1°. The 3D reconstruction of the tilt series was performed using the SIRT algorithm. The volume rendering, digital slicing and images were processed using Avizo.’.

4. What are the physical mechanisms and driving force for the NPLs to stack with gradual rotational variation?

Response: We thank the reviewer for this comment. The driving force for this stacking geometry probably stems from the ligand-induced mechanical stress on $CsPbBr_3$ NPLs, which has been studied in detail for stacked CdSe NPLs (Ref-23: *Science Advances* **2017**, *3 (9)*, e1701483.). Essentially, in an isolated NPL, the ligands on surface are free to rotate. However, after stacking with other NPLs, the ligands lock each other and such rotation freedom is limited (*Nano Lett.* **2016**, *16*, 4, 2133 - 2138; *Nano Lett*. **2016**, *16*, 4, 2127 - 2132). This translates into a reduction of the entropy and an increase of the Gibbs energy in the stacked system, generating extra mechanical stress that twists this NPL to partially recover the rotation freedom of the ligands (*Science Advances* **2017**, *3* (9), e1701483.).

Action: We have added this proposed rotation mechanism in the manuscript as follows:

‘The driving force for this stacking geometry probably stems from the ligand-induced mechanical stress on $CsPbBr_3$ NPLs, which has been studied in detail for stacked CdSe NPLs (Ref-23: *Science Advances* **2017**, *3* (9), e1701483.). Essentially, in an isolated NPL, the ligands on surface are free to rotate. However, after stacking with other NPLs, the ligands lock each other and such rotation freedom is limited (*Nano Lett.* **2016**, *16*, 4, 2133 - 2138; *Nano Lett*. **2016**, *16*, 4, 2127 - 2132). This translates into a reduction of the entropy and an increase of the Gibbs energy in the stacked system, generating extra mechanical stress that twists this NPL to partially recover the rotation freedom of the ligands (*Science Advances* **2017**, *3* (9), e1701483.)’.

We have cited the rotation entropy related references as ref-57 and 58.

5. Even the NPLs are stacked according to Fig. 4g and 4h, detailed electromagnetic simulations are required to show CPL can be generated.

Response: We thank the reviewer for the suggestion, following which we have performed electromagnetic simulations on left-handed (LH) and right-handed (RH) one-dimensional superlattices using the COMSOL Wave Optics Module (Figure R9). The experimentally measured PL spectrum was used as the incident light source, which,

after modulation by the helical structure (Figure R9a-b), produced the circularly polarized luminescence (CPL) spectrum (Figure R9c). The incident light was directed vertically from the top port, and the left- and right-handed polarized signals modulated by the nanostructure were collected at the bottom port. numerical simulations were performed for the left-handed and right-handed one-dimensional superlattices, respectively, assuming linearly polarized light excitation. The simulated CPL characteristic peaks of the two chiral films are in agreement with the experimental measurements (Figure 3e), and the simulated g-factors was of the order of 10^{-2} (Figure R9d).

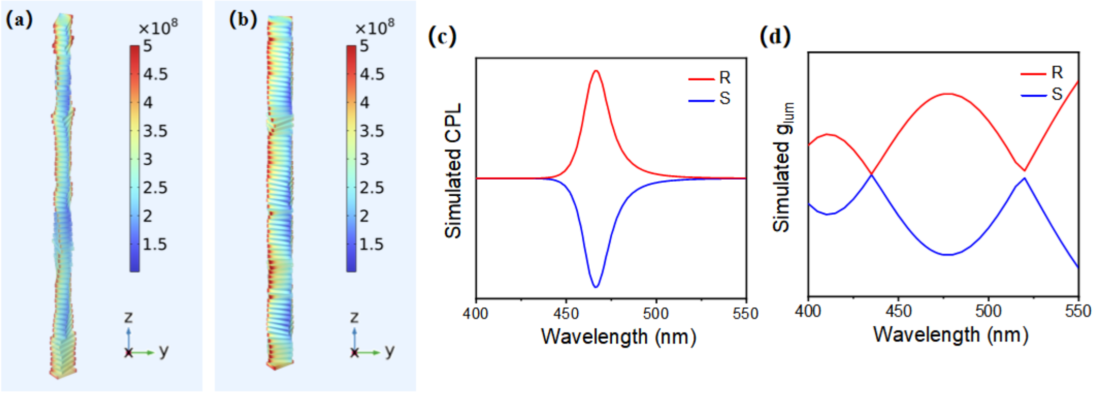

Figure R9. (a-b) Electric field passing through (a) right-handed chiral superlattice and (b) left-handed chiral superlattice. (c) Calculated CPL response and (d) g_{lum} curve by COMSOL Wave Optics Module.

Action: We have added this simulation result as Figure 6a-d in manuscript, and we added the corresponding discussion:

'Electromagnetic simulations were performed on left-handed (LH) and right-handed (RH) one-dimensional superlattices using the COMSOL Wave Optics Module (Figure 6a-d). The experimentally measured PL spectrum was used as the incident light source, which, after modulation by the helical structure (Figure 6a-b), produced the circularly polarized luminescence (CPL) spectrum (Figure 6c). The results show that the simulated CPL characteristic peaks of the two chiral films are in strong agreement with the experimental measurements (Figure 3e), and the simulated g-factors was of the order of 10^{-2} (Figure 6d)'.

We have added the calculation details in the 'Materials and methods' section of the SI as follows:

'All numerical calculations were performed via finite-element simulations using the COMSOL software. Floquet periodic boundary conditions were applied in the transverse directions of the unit cell to define the wavevector, while a perfectly matched layer (PML)

was adopted in the z-direction to simulate a reflection-free infinite domain. Simulations were carried out on structures modeled with left-handed and right-handed chirality. The incident light was directed vertically from the top port, and the left- and right-handed polarized signals modulated by the nanostructure were collected at the bottom port. Under linearly polarized light excitation, numerical simulations were performed for the left-handed and right-handed one-dimensional superlattices, respectively'.

6. The authors report enhanced g_{lum} in their ASE without detailed understanding. The authors only cited a few references and suggested the cavity effects. Please carry out detailed optical simulations to support the claims. Also, there are many samples presented in Fig. 2. Is there any correlation between the degree of linearly polarized emission vs CPL g_{lum} vs ASE $g_{lum?}$ Please carefully and statistically characterized more samples to draw a conclusion.

Response: We thank the reviewer for the comment. Following the suggestion, optical simulations were performed to demonstrate the optical microcavity effect in the 1D superlattices (Figure R10). The results indicate that the 1D superlattices can naturally form effective Fabry-Perot (FP) optical cavities, providing feedback for laser oscillation. This proves that the system possesses a favorable optical cavity effect. The photon loss within the cavity is relatively low and the resonance capability is strong.

At present, only NPLs-OAm based 1D superlattices exhibit a chiral ASE effect, while the other 1D superlattices (NRs-DDAm based one) does not show any chiral ASE effect under the same pumping densities (35 μJ/cm^2). Hence, for that sample, we cannot establish a correlation among the linearly polarized emission, the CPL g_{lum} and the ASE g_{lum}. We propose that the higher anisotropic shape of the NPLs-OAm contributes to the higher aspect ratio of the 1D superlattices (Figure 1m), and thus a higher linear and circularly polarized emission (Figure 3f and Figure 5c), leading to a detectable chiral ASE effect (Figure 6e-f). In the microcavity, photons are confined and oscillate repeatedly. During each round trip, the right-handed circularly polarized (RCP) compo nent experiences greater gain than the left-handed circularly polarized (LCP) component. After hundreds or thousands of round trips of amplification, the small inherent differences are continuously accumulated and amplified, leading to a rapid increase in the output g_{ASE} value, which far exceeds the intrinsic g_{lum} value.

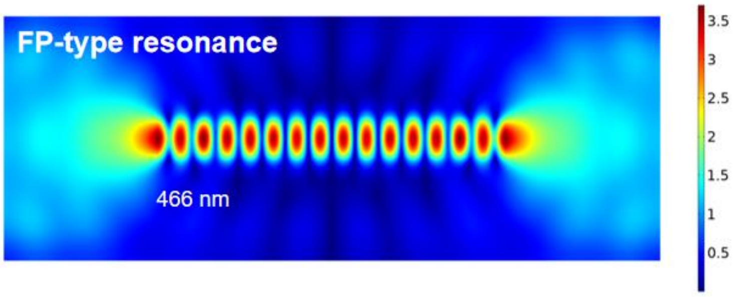

Figure R10. Simulated field intensity distribution (λ=466 nm, n= 2.6) in a superlattice with L= 1.7 μm. The simulated field intensity distribution indicates that the superlattice can function as a Fabry–Pérot (FP) optical cavity for realizing laser oscillations.

Action: We have added Figure R10 as Figure 6i in the manuscript and added the following text in the manuscript:

'Optical simulations were performed to demonstrate the optical microcavities effect of 1D superlattices (Figure 6i). The results indicate that the 1D superlattices can naturally form effective Fabry-Perot (FP) optical cavities, providing feedback for laser oscillation. This proves that the system possesses a favorable optical cavity effect.'. And 'In the microcavity, photons are confined and oscillate repeatedly. During each round trip, the right-handed circularly polarized (RCP) component experiences greater gain than the left-handed circularly polarized (LCP) component. After hundreds or thousands of round trips of amplification, the small inherent differences are continuously accumulated and amplified, leading to a rapid increase in the output g_{ASE} value, which far exceeds the intrinsic g_{lum} value.'

7. It would be great if the authors can quantify the dipole orientation of CPL for the prepared films using k-space microscopy or angular resolved PL with linear polarizer.

Response: We thank the reviewer for this suggestion. In our case, the dissymmetrical factor (g_{lum}) is of the order of 10^{-2}, which is too small to show detectable PL intensity differences (~1%) in the angular resolved PL.

Action: We have added the following sentence in the manuscript: 'The dissymmetrical factor (g_{lum}) is of the order of 10^{-2}, which is too small to show detectable PL intensity differences (~1%) in the angular resolved PL.'.

Reviewer #2 (Remarks to the Author):

The authors reported on the self-assembly of $CsPbBr_3$ nanoplatelets for the preparation of 1D superlattices of varying lengths. The nanoplatelets stack into ribbons while the shape of the nanoplatelets influences the aspect ratio of the superlattices. The superlattices exhibit strong polarized emission owing to the anisotropic shape. The superlattices stack into a chiral configuration along the length due to the orientational twist of these nanoplatelets. The chiral configuration of the superlattices results in circular dichroism and circularly polarized luminescence. The chirality is controlled after the addition of chiral ligands exchanging the pristine ligands. A moderate dissymmetrical factor is obtained from the self-assemblies of the nanoplatelets. The helical assembly of the nanoplatelets and the chiroptical properties are interesting. I recommend publication of this paper in Nature Communications after the authors have considered and addressed the following comments:

Comments:

(i) The synthesis of the nanoplatelets were carried out using didodecylamine (DDDAm) and oleic acid as surfactants. Whether the lateral spacing between the adjacent nanoplatelets has any correlation with the lengths of these ligands.

Response: We thank the reviewer for the comment. The space between the adjacent NPLs or NRs is indeed strong correlated with the lengths of the surface ligands.

(1) For the vertical direction (along the long axis of the 1D superlattices), the inter-nanoparticle spacing (d_z) can be directly calculated based on SAXS data (Figure R11 below), by the formula $d_z = 2\pi/q - d_0$, where ($2\pi/q$) is the stacking distance measured by SAXS and d_0 is the thickness of the NPLs (or NRs). For example, the alkyl chain lengths (L) of DDDAm and OAm are ~1.5 nm and ~1.7 nm, respectively, and the corresponding d_z between adjacent NRs/NPLs is ~2.3 nm and ~3.0 nm, respectively (Figure R11). d_z is generally smaller than twice the length of the ligands (2L), due to partial interdigitation of adjacent ligands. The interdigitation factor η can be calculated as $\eta = (d_z/2L) * 100\%$, in a range of 77-80%. Overall, the inter-nanoparticle spacing between adjacent NRs/NPLs increases following the increase of alkyl chain lengths of ligands.

(2) For the lateral direction (along the short axis of the 1D superlattices), the lateral spacing (d_x) was estimated from stacked NRs/NPLs in the TEM images (Figure R11c-d).
S
u
c
h

l
a
t
e

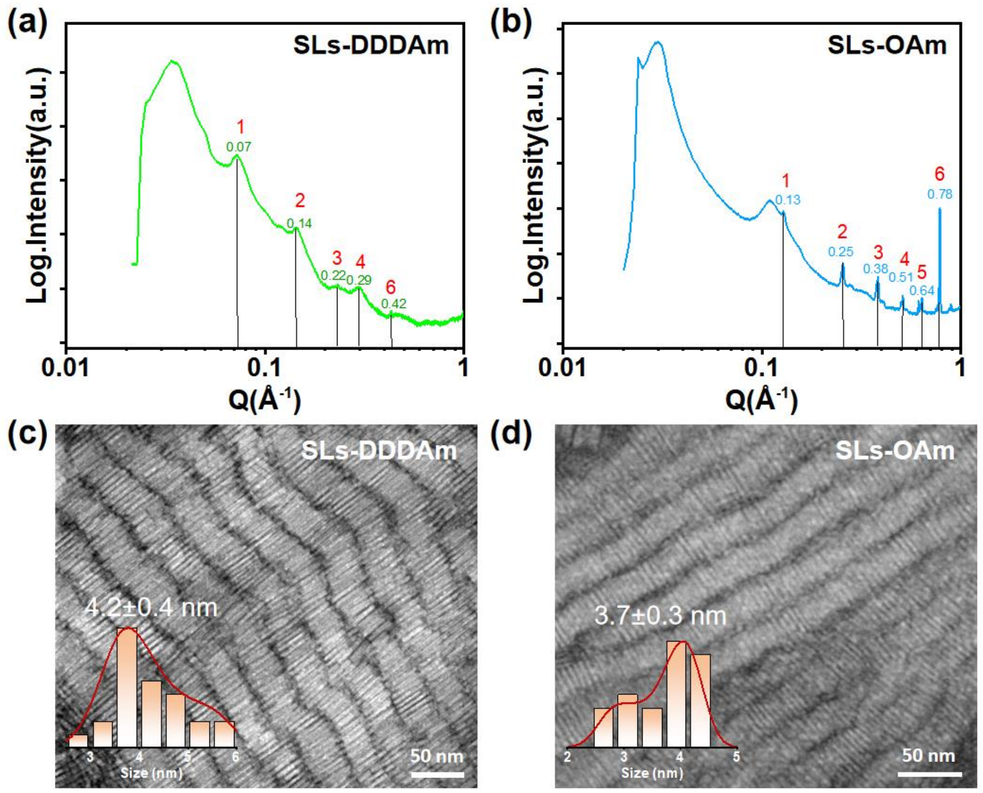

Figure R11. (a-b) SAXS data of (a) SLs-DDDAm and (b) SLs-OAm. (c-d) TEM images and size distograms of (

Action: We have added the SAXS data as Figure S10 in the Supporting Information. The following text has been added to the manuscript:

'For the vertical direction (along the long axis of the 1D superlattices), the inter-nanoparticle spacing (d_z) can be directly calculated based on SAXS data (Figure R11 below), by the formula $d_z = 2\pi/q - d_0$, where ($2\pi/q$) is the stacking distance measured by SAXS and d_0 is the thickness of the NPLs (or NRs). For example, the alkyl chain lengths (L) of DDDAm and OAm is ~1.5 nm and ~1.7 nm, respectively, and the lateral spacing (d_x) between adjacent NRs/NPLs is ~2.3 nm and ~3.0 nm, respectively (Figure R11). d_z is generally smaller than twice the length of the ligands (2L), due to partial interdigitation of ligands. The interdigitation factor η can be calculated as $\eta = (d_z/2L)$ *100%, in a range of 77-80%. Overall, the inter-nanoparticle spacing between adjacent NRs/NPLs increases following the increase in alkyl chain lengths of the ligands.'.

The following references (*ACS Nano* **2021**, *15*, 20341−20352; *Nano Lett*. **2020**, *20*, 3673−3680) have been cited as Ref-31 and 32.

(ii) The length of the superlattices of the nanoplatelets was increased by reducing the thickness of the nanoplatelets. How the nanoplatelets are stacked into twisted configuration? The authors should comment on this.

Response: We thank the reviewer for this comment. To directly investigate the stacking of NPLs in the superlattices (NPLs-OAm based 1D superlattices), we employed 3D reconstruction based on scanning TEM. As shown in Figure R7, four orthoslices (Figure R7b-e) of stacked NPLs have been collected and used to reconstruct a 3D image (R7a). The corresponding video for the 3D structure is added as supplementary information (Video R1) that support the rotated NPLs in the fibrillar structure. Accordingly, 3D FFT analysis of the stacked NPLs was carried out, as shown in Figure R8. The discrete dots (highlighted by the red arrows) indicate a distorted lamellar pattern between the stacked NPLs, in analogy with previous works (*Journal of Structural Biology,* **2012**, *178*, 152 - 164; *Am. J. Phys*, **2018**, *86*, 95 - 104). These results support our hypothesis that the NPLs are stacked along their long axis with different rotation angles relative to each other.

The driving force for this stacking geometry probably stems from the ligand-induced mechanical stress on $CsPbBr_3$ NPLs, which has been studied in detail for stacked CdSe NPLs (Ref-23: *Science Advances* **2017,** *3 (9),* e1701483.). Essentially, in an isolated NPL, the ligands on surface are free to rotate. However, after stacking with other NPLs, the ligands lock each other and such rotation freedom is limited (*Nano Lett.* **2016,** *16,* 4, 2133–2138; *Nano Lett.* **2016,** *16,* 4, 2127–2132). This translates into a reduction of the entropy and an increase of the Gibbs energy in the stacked system, generating extra mechanical stress that twists this NPL to partially recover the rotation freedom of the ligands. (*Science Advances* **2017,** *3 (9),* e1701483.).

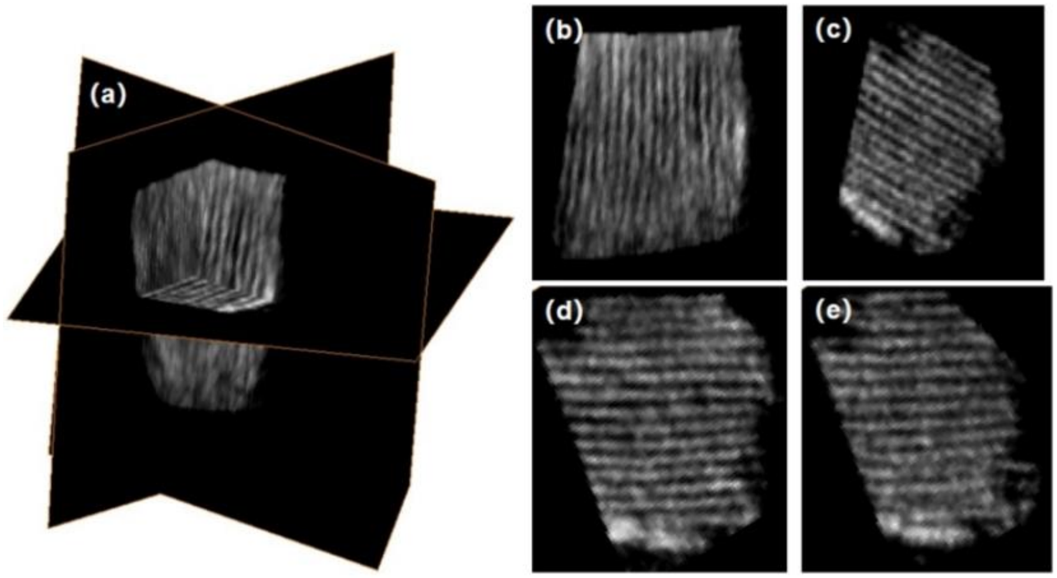

Figure R7. 3D reconstruction of the superlattices obtained by electron tomography, showing the reconstructed 3D volume and orthoslices from different projections.

Vedio 1.mp4

Video R1. 3D reconstruction video of the superlattices obtained by electron tomography.

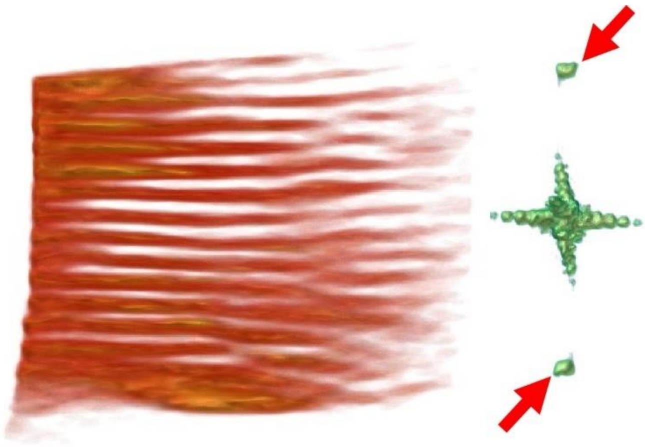

Figure R8. 3D reconstructed volume and 3D fast Fourier transform (FFT) analysis on the reconstructions of the stacked NPLs.

Action: We have added Figure R8 as Figure 5i-l in the manuscript, along with the following discussion:

'To directly investigate the stacking of NPLs in the superlattices (NPLs-OAm based 1D superlattice), we employed 3D reconstruction based on scanning TEM. As shown in Figure R7, four orthoslices (Figure R7b-e) of stacked NPLs were collected, and used to reconstruct a 3D image (Figure R7a). The corresponding video for the 3D structure is added as supplementary information (Video R1). 3D FFT analysis of the stacked NPLs was carried out, as shown in Figure 5k-l. The discrete dots (highlighted by the red arrows) indicate a distorted lamellar pattern between the stacked NPLs, in analogy with previous works. (*Journal of Structural Biology*, **2012**, *178*, 152–164; *Am. J. Phys*, **2018**, *86,* 95–104). These results support our hypothesis that the NPLs are stacked along their long axis with different rotation angles relative to each other.'.

We have added this proposed rotation mechanism in the manuscript as follows: 'The driving force for this stacking geometry probably stems from the ligand-induced mechanical stress on $CsPbBr_3$ NPLs, which has been studied in detail for stacked CdSe NPLs (Ref-23: *Science Advances* **2017**, *3 (9)*, e1701483.). Essentially, in an isolated NPL, the ligands on surface are free to rotate. However, after stacking with other NPLs, the ligands lock each other and such rotation freedom is limited (*Nano Lett*. **2016**, *16*,

4, 2133 - 2138; *Nano Lett.* **2016**, *16*, 4, 2127 - 2132). This translates into a reduction of the entropy and an increase of the Gibbs energy in the stacked system, generating extra mechanical stress that twists this NPL to partially recover the rotation freedom of the ligands (*Science Advances* **2017**, *3 (9)*, e1701483.)' .We have cited the rotation entropy related reference as ref-57 and 58.

We have added Figure R7 and Video S1 in the Supporting Information. We have cited the references as ref-55 and 56. (*Journal of Structural Biology*, **2012**, *178*, 152 - 164; *Am. J. Phys*, **2018**, *86*, 95 - 104.)

We have added the sample preparation and testing methods for the electron tomography analysis the SI as follow:

'Tomography series were acquired using an aberration corrected "cubed" Thermo Fisher Scientific Titan 60-300 electron microscope operated at 120 kV with a single-tilt tomography holder over a tilt range from −68° to +72°, with tilt increments of 1°. The 3D reconstruction of the tilt series was performed using the SIRT algorithm. The volume rendering, digital slicing and images were processed using Avizo'.

(iii) When oleylamine was used in the synthesis, the thickness of the reduced to 4.6 nm and the lateral size became ~29 nm. The length of the ribbons became ~1000 μm with a width of ~0.8 μm. This observation implies that the ribbons consist of many nanoplatelets along the width and lengths of the ribbons. What is the orientation of the individual nanoplatelets inside the ribbon?

Response: We thank the reviewer for the comment. The NPLs/NRs stack with each other both along their thickness direction (along the long axis of 1D superlattices) and their lateral direction (along the short axis of 1D superlattices). We have labeled the x-z directions in Figure R12 to show this relation. In detail, the length of the 1D superlattices (L_{SL}) follows the formula L_{SL} = N*d (where N is the number of NRs/NPLs along the long axis, and d is the sum of thickness of a single NPL and the length of a partial interdigitated ligand layer). Correspondingly, the width of the 1D superlattices (W_{SL}) follows the formula W_{SL} = M*l (where M is the number of NRs/NPLs along the short axis, and l is the lateral size of a single NR/NPL).

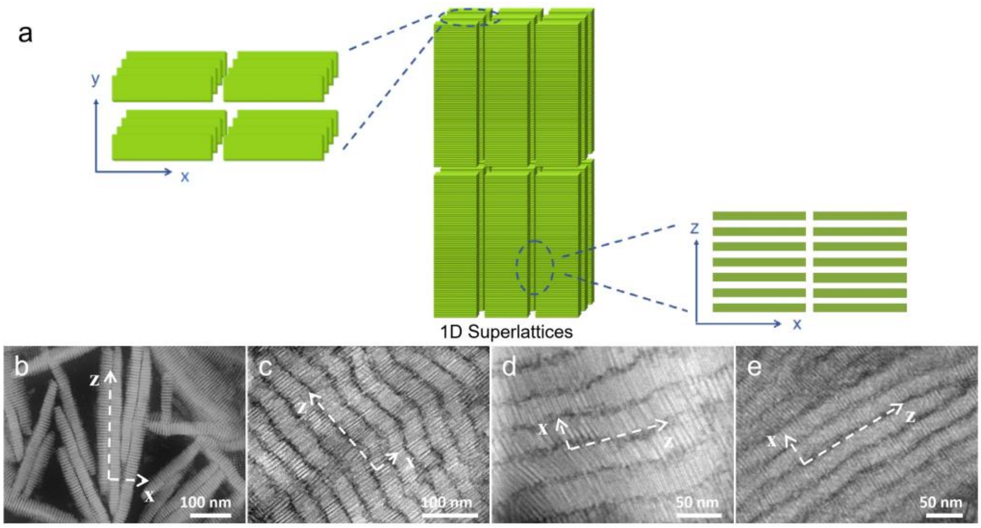

Figure R12. (a) Scheme of the stacking directions of NPLs/NRs in 1D superlattices;(b-e) STEM characterization of 1D NPL/NR superlattices.

Action: We have added coordinate axes to the TEM images in Figures 1d-p: the z-axis is along the length direction of the 1D superlattices, and the x-axis is along the width direction. We have added Figure R12 as Figures S9 in the Supporting Information. The following text has been added to the manuscript:

‘The NRs/NPLs stack with each other both along their thickness direction (along the long axis of 1D superlattice) and their lateral direction (along the short axis of the 1D superlattice), as shown in Figure R12. We have labeled the x-z directions in Figure 1c-o to show this relation. In detail, the length of the 1D superlattices (L_{SL}) follows the formula L_{SL}= N*d (where N is the number of NRs/NPLs along the long axis, and d is the sum of thickness of a single NPL and the length of a partial interdigitated ligand layer). Correspondingly, the width of the 1D superlattices (W_{SL}) follows the formula W_{SL} = M*l (where M is the number of NRs/NPLs along the short axis, and l is the lateral size of a single NR/NPL).’.

(iv) The individual $CsPbBr_3$ nanoplatelets are polarized owing to their anisotropic shapes, with the randomly oriented nanoplatelets show lesser degree of polarization compared to the ribbons. How the polarization was measured? Is there any correlation with the excitation polarizer and nanoplatelets orientation?

Response: We thank the reviewer for the comment. The polarization measurements were carried out on an Edinburgh FLS980, equipped with a polarizer behind the emission lens to control the polarization direction. The 1D superlattices was placed on the solid sample holder. Under excitation light irradiation, the polarizer was rotated from 0° to 360° in steps of 30°, and the PL spectra at different polarization angles were collected sequentially. The maximum fluorescence intensity I_{max} and

minimum fluorescence intensity I_{min} were extracted, and the polarization degree P was calculated according to Equation (1):

$$P = \frac{I_{max}-I_{min}}{I_{max}+I_{min}} \quad (1)$$

The increase in linear polarization is strongly related to the degree of order of the 1D superlattices. The 1D superlattices were obtained only under confined evaporation conditions (Figure R3), while the randomly assembled NRs were obtained by randomly evaporation (i.e. not following the confined evaporation procedure). Optical microscopy images and polar plots of the normalized intensity of the photoluminescence emission as a function of detection angle for random assembled NRs and 1D superlattices are shown in Figure R5. The randomly assembled NRs sample exhibits no clear orientation (Figure R5a), corresponding to a weak polarized emission (P = 0.16, Figure R5c). In contrast, the 1D superlattices sample shows strong orientation (Figure R5b) and significantly stronger polarized emission (P = 0.72, Figure R5d).

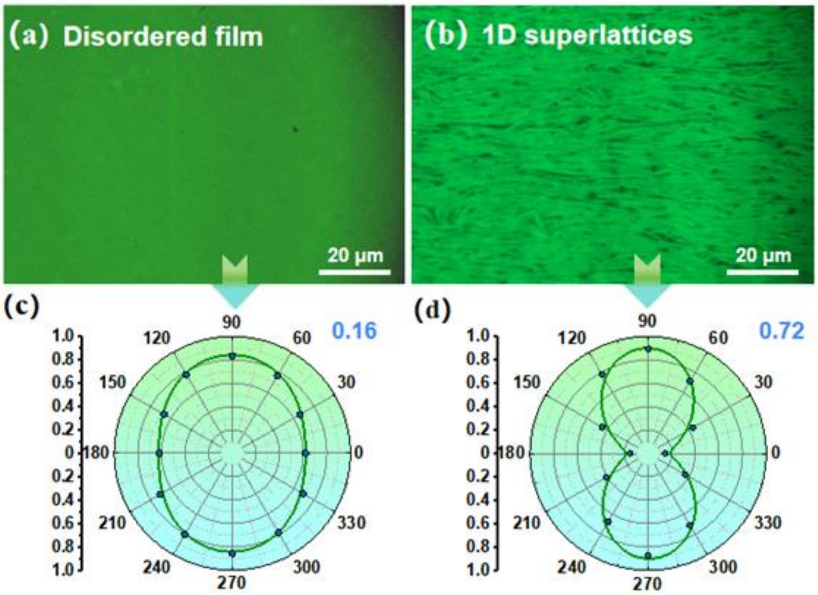

Figure R5. (a-b) Optical microscopy images of (a) a disordered NRs-DDDAm-1 film prepared by random evaporation assembly and (b) a 1D NRs-DDDAm-1 superlattices film prepared by confined evaporation; (c-d) Polar plots of the normalized intensity of the photoluminescence emission as a function of detection angle for (c) disordered NRs-DDDAm-1 film and (d) 1D NRs-DDDAm-1 superlattices film.

To analyze the polarized emission direction of the samples, we employed a simplified polarization system based on a RX50M upright metallurgical microscope. This system consists of a polarizer and an analyzer, where the detection angle can be tuned by rotating the analyzer (0 - 360°). As shown in Figure R6, the fluorescence intensity

o

f

t

h

, respectively, which indicates that the polarization direction is in perpendicular to the long axis of the 1D superlattices, or parallel to the long axis of the NRs/NPLs. This indicates that the polarized emission stems from the building blocks (NPLs/NRs) of the 1D superlattices. This was already stated in the manuscript, (apart from the addition of ”NR”): ‘Due to their anisotropic shape, individual $CsPbBr_3$ NPLs/NRs are known to show polarized emission. A random assembly of NPLs on the other hand can partially average out this polarization. The ordered 1D superlattices of this work are instead built from oriented NPLs/NRs and are thus expected to preserve the polarized emission.’. The related phenomenon can be also found in *Nature Photonics*, **2024**, *18*, 586–594 and *Nano Letters*, **2014**, *14*, 710–715.

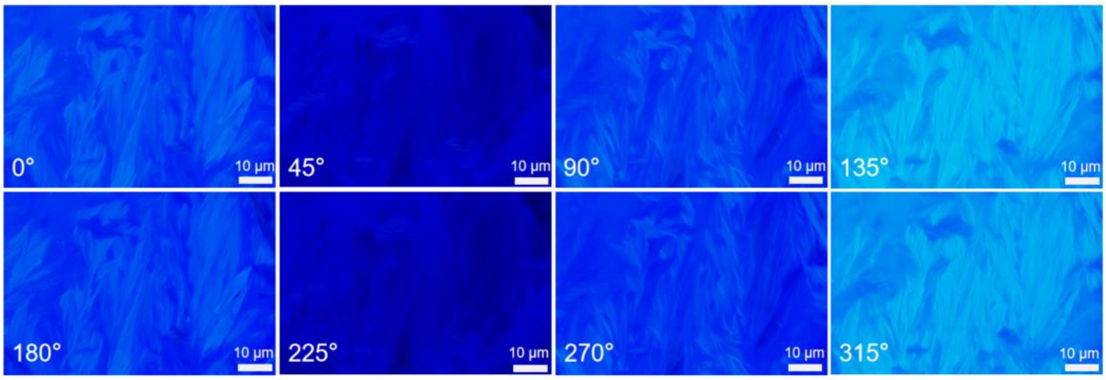

Figure R6. (a-h) Polarized fluorescence optical micrographs taken under crossed polarizers for film by rotating 1D SLs-OAm films by different angles from 0° to 315°. (The film was rotated in 45° increments from 0° to 315°.)

Action: To study the relations between the orientation of the superlattices and linear polarization, we have added the control experiment (Figure R5) and the orientation dependent polarized emission (Figure R6) as Figures S10 and Figure S12 in the Supporting Information. The corresponding discussion have been added the following text in the manuscript:

‘To analyze the polarized emission direction of the samples, we employed a simplified polarization system based on a RX50M upright metallurgical microscope in this study. This system consists of a polarizer and an analyzer, where the detection angle can be tuned by rotating the rotatable analyzer (0–360°). As shown in Figure S12, the fluorescence intensity of the sample exhibits a trend of “increase-decrease-

i

n

c

r

e

a

s

e

-

We have cited the references as ref-17 and 42. (*Nature Photonics*, **2024**, *18*, 586 – 594 and *Nano Letters*, **2014**, *14*, 710 – 715.)

(v) The intrinsic chiral origin of $CsPbBr_3$ nanoplatelets is unclear. The distorted PbBr6 octahedra may also incorporate intrinsic chirality in these halide perovskite nanocrystals. The authors should comment on this. Also, the dissymmetrical factor (g_{abs}) of CD may be useful.

Response: We thank the reviewer for the suggestion. We agree that the distorted lattice may also contribute intrinsic chirality in the absence of chiral ligands, which is well established in cube-connected twisted $CsPbBr_3$ nanowires and $CsPbBr_3$ nanoclusters (*J. Am. Chem. Soc.* **2024,** 146, 33883−33892; *J. Phys. Chem. Lett.* **2023,** *14,* 10630−10633.). This rotated stacked NPLs in 1D superlattices likely exhibit similar distorted lattice and contribute to the chirality. It is worth to note that the randomly assembled NPLs exhibit non-detectable CD response, while the 1D NPLs-OAm superlattices show a reasonable CD signal (Figure R13 below). This indicates that the ordered macroscopic pattern plays a key role on preserving or amplifying the intrinsic chirality.

Following the reviewer's suggestion, we have evaluated the g_{abs} of the CD spectra reported in the manuscript (Figures R15-16). For the NRs/NPLs based superlattices they are in the 10^{-3} to 10^{-4} range, while for the NPLs based superlattices they are higher.

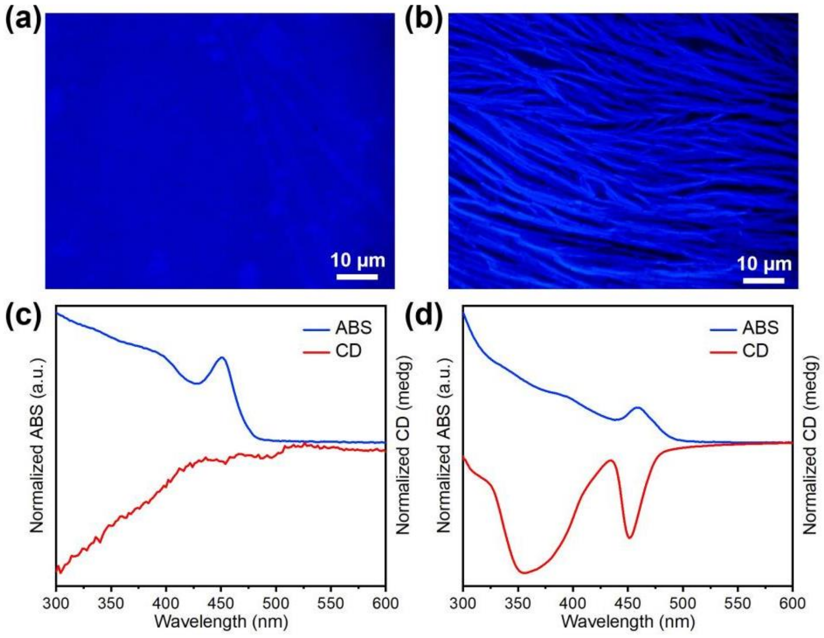

Figure R13. (a-b) Optical microscope images of the disordered film and the 1D SLs-OAm film. (c-d) CD signals of the disordered film and the 1D SLs-OAm film.

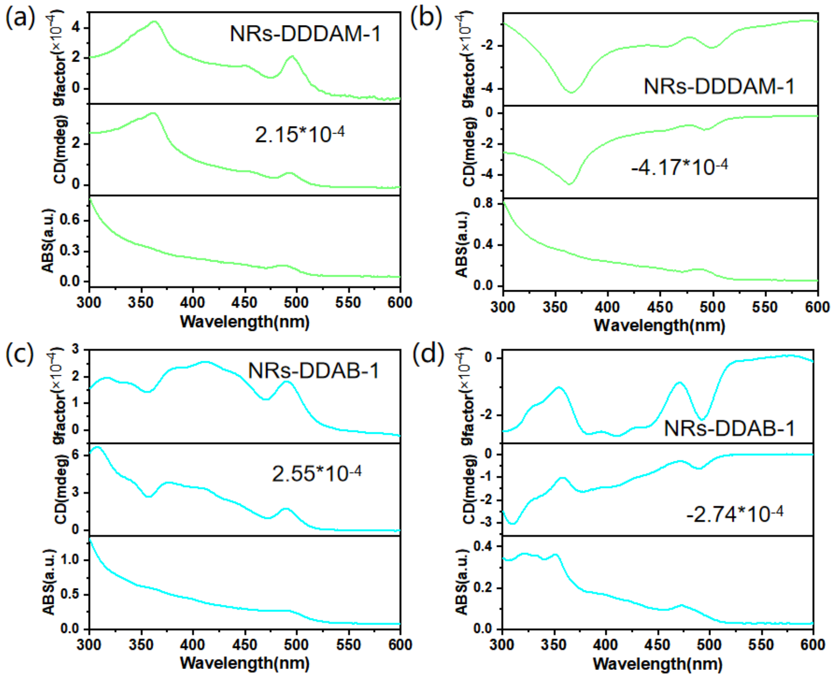

Figure R14. (a-b) g_{abs} data of 1D superlattices assembled from NRs-DDDAm-1; (c–d) g_{abs} data of 1D superlattices assembled from NRs-DDAB-1.

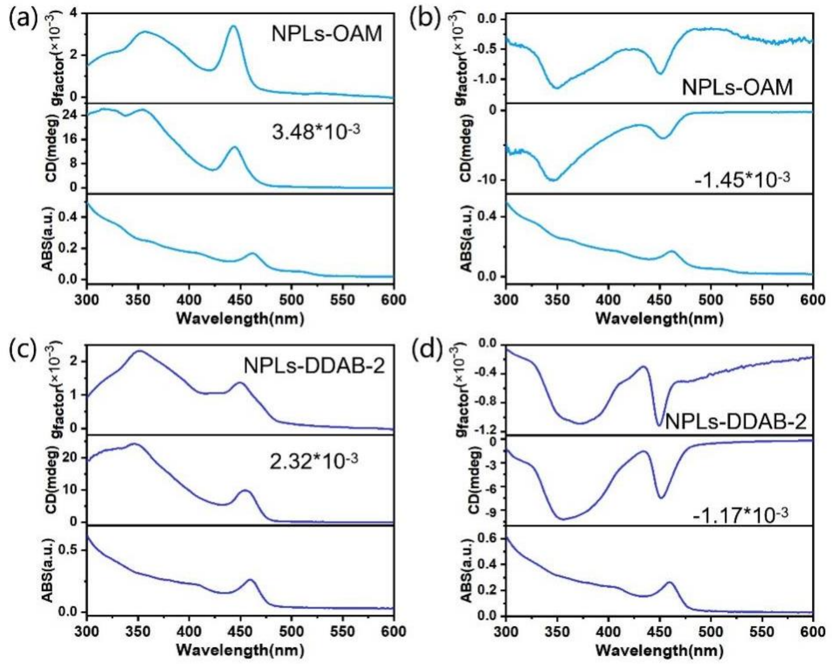

Figure R15. (a–b) g_{abs} data of 1D superlattices assembled from NPLs-OAm; (c–d) g_{abs} data of 1D superlattices assembled from NPLs-DDAB-2.

Action: We have added the discussion on origin of chirality in manuscript as 'These rotated stacked NPLs in 1D superlattices likely exhibit distorted lattice and contribute to the chirality, similar to non-chiral ligands involved chiral $CsPbBr_3$ nanowires and nanoclusters [ref-24 and 44]. It is worth to note that the randomly assembled NPLs

exhibit non-detectable CD response, while the 1D NPLs-OAm superlattices show a reasonable CD signal (g_{abs}= is of the order of 10^{-3}) (Figure R13). This indicates that the ordered macroscopic pattern plays a key role in preserving or amplifying the intrinsic chirality.';

The related references (*J. Am. Chem. Soc.* **2024,** *146,* 33883−33892; *J. Phys. Chem. Lett.* **2023,** *14,* 10630−10633) have been cited as ref-24 and 44;

The g_{abs} result (Figure R14-15) has been supplied as Figure S16-17 in Supporting Information.

(vi) Nanocrystals exhibiting circularly polarized luminescence are of great interest, yet the distinction of circularly polarized luminescence is not made clear in the text. What is the origin of circularly polarized luminescence in the $CsPbBr_3$ nanoplatelets?

Response: We thank the reviewer for the suggestion, following which we have performed electromagnetic simulations on left-handed (LH) and right-handed (RH) stacked NPLs using the COMSOL Wave Optics Module (Figure R10). The experimentally measured PL spectrum was used as the incident light source, which, after modulation by the helical structure (Figure R10a-b), produced the circularly polarized luminescence (CPL) spectrum (Figure R10c). The incident light was directed vertically from the top port, and the left- and right-handed polarized signals modulated by the nanostructure were collected at the bottom port. Numerical simulations were performed for the left-handed and right-handed one-dimensional superlattices, respectively, assuming linearly polarized light excitation. The simulated CPL characteristic peaks of the two chiral films are in agreement with the experimental measurements (Figure 3e), and the simulated g-factors was of the order of 10^{-2} (Figure R10d).

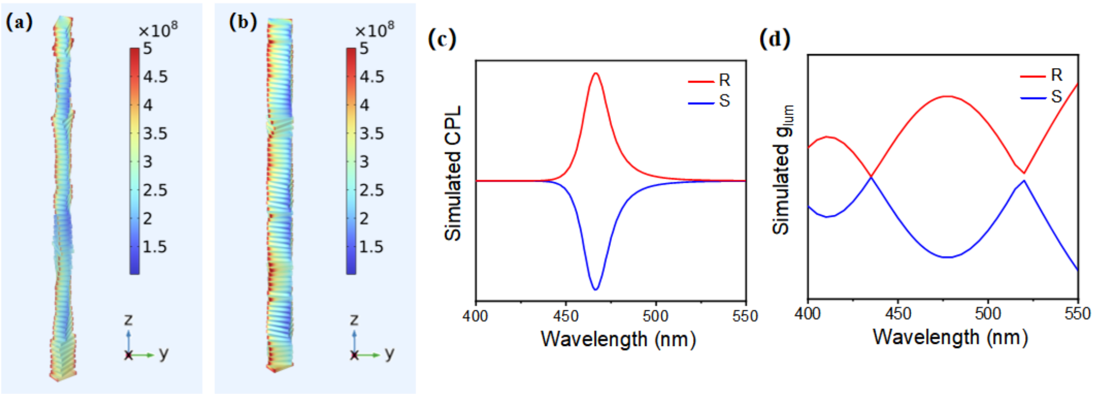

Figure R9. (a-b) Electric field passing through (a) right-handed chiral superlattice and (b) left-handed chiral superlattice. (c) Calculated CPL response and (d) g_{lum} curve by COMSOL Wave Optics Module.

Action: We have added these simulations as Figure 6a-d in manuscript, and we

added the corresponding discussion:

'Electromagnetic simulations were performed on left-handed (LH) and right-handed (RH) one-dimensional superlattices using the COMSOL Wave Optics Module (Figure 6a-d). The experimentally measured PL spectrum was used as the incident light source, which, after modulation by the helical structure (Figure 6a-b), produced the circularly polarized luminescence (CPL) spectrum (Figure 6c). The results showed that the simulated CPL characteristic peaks of the two chiral films are in strong agreement with the experimental measurements (Figure 3e), and the simulated g-factors was of the order of 10^{-2} (Figure 6d)'.

We have added the calculation details in the 'Materials and methods' section of the SI as follows:

'All numerical calculations were performed via finite-element simulations using the COMSOL software. Floquet periodic boundary conditions were applied in the transverse directions of the unit cell to define the wavevector, while a perfectly matched layer (PML) was adopted in the z-direction to simulate a reflection-free infinite domain. Simulations were carried out on structures modeled with left-handed and right-handed chirality. The incident light was directed vertically from the top port, and the left- and right-handed polarized signals modulated by the nanostructure were collected at the bottom port. Under linearly polarized light excitation, numerical simulations were performed for the left-handed and right-handed one-dimensional superlattices, respectively'.

(vii) Could the authors show film thickness- and orientation-dependent CD curves for the nanoplatelets?

Response: We thank the reviewer for this suggestion. (1) To evaluate the thickness dependent CD spectra, we have used different concentrated NPLs solutions (5-20 mg/mL) to prepare the SLs film with different thicknesses. As shown in Figure R17 below, the average thickness is determined by cross-sectional SEM. It decreases from 328 nm to 235 nm and to 145 nm, following the decrease in concentration from 20 mg/mL to 10 mg/mL and to 5 mg/mL. The absorption spectra and the CD spectra were collected on all the samples and the corresponding dissymmetry factors (g_{abs}) were calculated. The results indicate that the dissymmetry factor (g_{abs}) is thickness dependent: a thicker SLs film has a larger value of g_{abs} (Figure R16i).

(2) To evaluate the CD dependence on film orientation, we performed CD measurements on the film samples using the so-called four-scan method (the sample is scanned at four different orientations, (1) θ = 0°, β = 0° (2) θ = 90°, β = 0° (3) θ = 0°, β = 180° and (4) θ = 90°, β = 180° and averages are made over the four scans) (Figure R17). This allowed us to subtract the contribution of linear dichroism to

the CD spectra, hence retaining only the “true CD” signal.

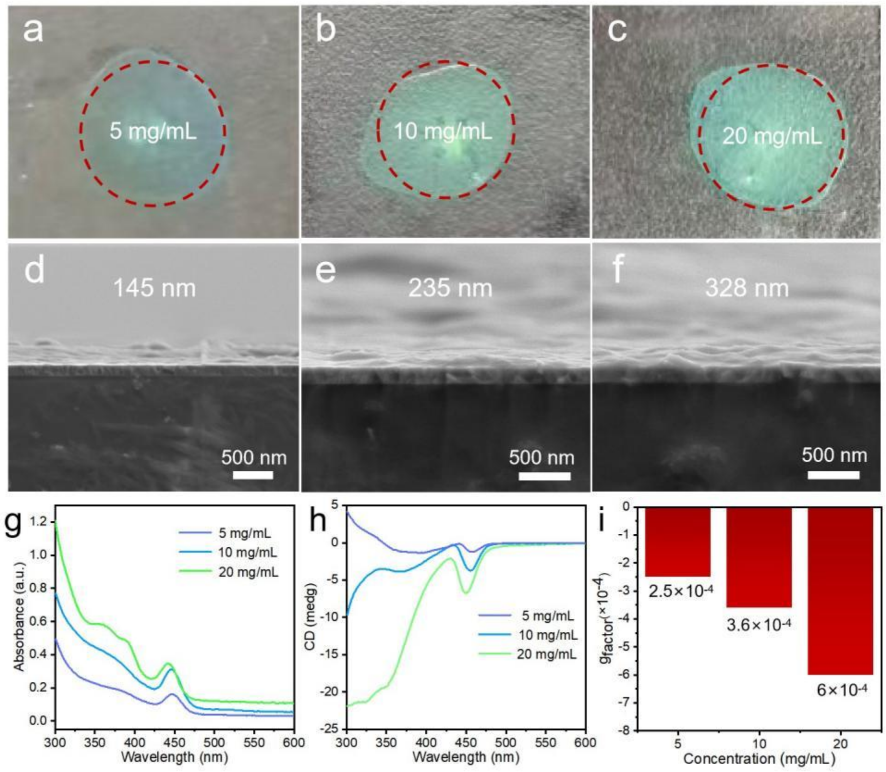

Figure R16. (a-c) SLs-OAm films prepared from NPLs solutions in toluene at different concentrations of NPLs, ranging from 5 mg/mL to 20 mg/mL. (d-f) The corresponding thickness values for the prepared SLs-OAm films were determined by cross sectional SEM. The average thickness was 322 nm for 20 mg/mL film, 230 nm for 10 mg/mL film, and 132 nm for 5 mg/mL film, respectively. (g) Absorption spectra, (h) CD spectra and (i) g_{abs} values of the various SLs-OAm films.

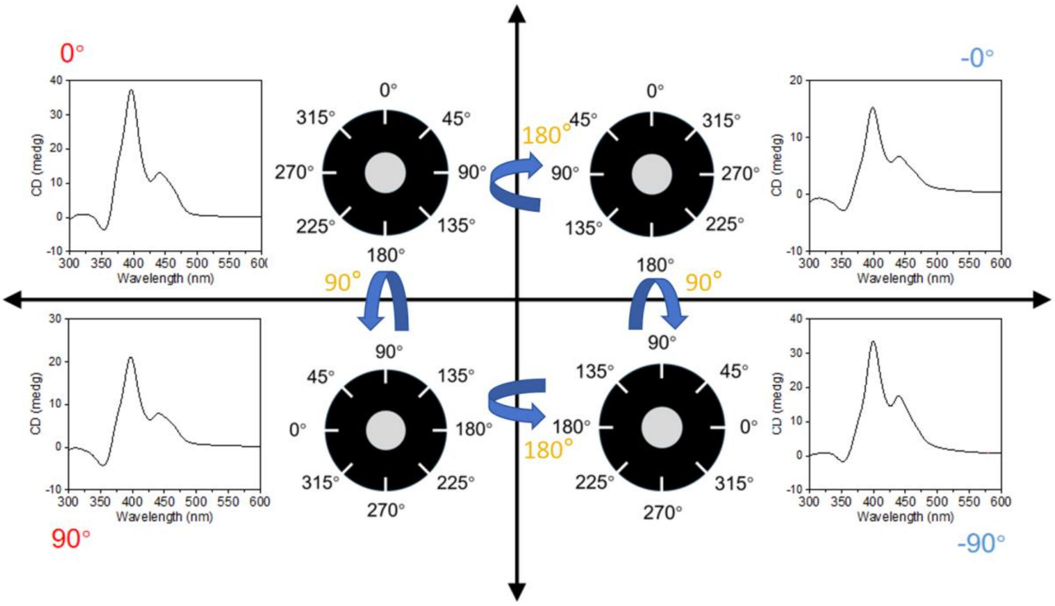

Figure R17. The glass slide with the SLs-OAm on was fixed on the testing frame, and the CD signal of the sample at (0°, 90°, -0° -90°) were collected, respectively. From 0° to -0° or 90° to -90° represent a flipping of sample.

Action: We have added the thickness- and orientation-dependent CD experiments as Figure S14-15 in Supporting Information. The following text has been added in the manuscript:

'To evaluate the thickness dependent CD spectra, we used different concentrated NPLs-OAm solutions (5-20 mg/mL) to prepare the SLs-OAm film with different thicknesses. As shown in Figure R16, the average thickness is determined by cross-sectional SEM. It decreases from 328 nm to 235 nm and to 145 nm, following the decrease in concentration from 20 mg/mL to 10 mg/mL and to 5 mg/mL. The absorption spectra and the CD spectra were collected on all the samples and the corresponding dissymmetry factors (g_{abs}) were calculated. The results indicate that the dissymmetry factor (g_{abs}) is thickness dependent: a thicker SLs film has a larger value of g_{abs} (Figure R16i). The CD spectra were collected using the so-called four-scan method (the sample is scanned at four different orientations, (1) θ = 0°, β = 0° (2) θ = 90°, β = 0° (3) θ = 0°, β = 180° and (4) θ = 90°, β = 180° and averages are made over the four scans) (Figure R17). This allowed us to subtract the contribution of linear dichroism to the CD spectra, hence retaining only the "true CD" signal.'.

(viii) The amplified spontaneous emission of 1D nanoplatelet superlattices is interesting, however, the description is too short. A detailed discussion may be useful.

Response: We thank the reviewer for the comment. We thank the reviewer for the comment. Optical simulations were performed to demonstrate the optical microcavity effect in the 1D superlattices (Figure R10). The results indicate that the 1D superlattices can naturally form effective Fabry-Perot (FP) optical cavities, providing feedback for laser oscillation. This proves that the system possesses a favorable optical cavity effect. The photon loss within the cavity is relatively low and the resonance capability is strong.

At present, only NPLs-OAm based 1D superlattices exhibit a chiral ASE effect, while the other 1D superlattices (NRs-DDAm based one) does not show any chiral ASE effect under the same pumping densities (35 μJ/cm^2). Hence, for that sample, we cannot establish a correlation among the linearly polarized emission, the CPL g_{lum} and the ASE g_{lum}. We propose that the higher anisotropic shape of the NPLs-OAm contributes to the higher aspect ratio of the 1D superlattices (Figure 1m), and thus a higher linear and circularly polarized emission (Figure 3f and Figure 5c), leading to a detectable chiral ASE effect (Figure 6e-f). In the microcavity, photons are confined and oscillate repeatedly. During each round trip, the right-handed circularly polarized (RCP) compo

nent experiences greater gain than the left-handed circularly polarized (LCP) component. After hundreds or thousands of round trips of amplification, the small inherent differences are continuously accumulated and amplified, leading to a rapid increase in the output g_{ASE} value, which far exceeds the intrinsic g_{lum} value.

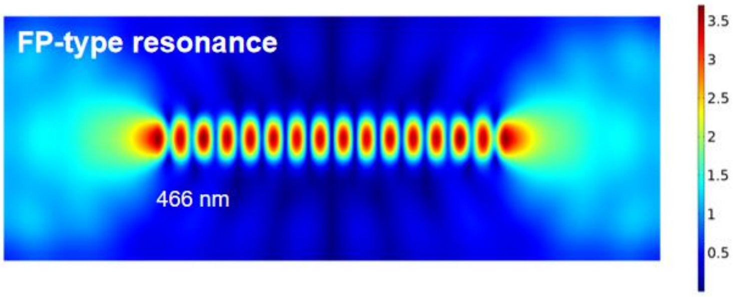

Figure R10. Simulated field intensity distribution (λ=466 nm, n= 2.6) in a superlattice with L= 1.7 μm. The simulated field intensity distribution indicates that the superlattice can function as a Fabry–Pérot (FP) optical cavity for realizing laser oscillations.

Action: We have added Figure R10 as Figure 6i in the manuscript and added the following text in the manuscript:

‘Optical simulations were performed to demonstrate the optical microcavities effect of 1D superlattices (Figure 6i). The results indicate that the 1D superlattices can naturally form effective Fabry-Perot (FP) optical cavities, providing feedback for laser oscillation. This proves that the system possesses a favorable optical cavity effect.’. And ‘In the microcavity, photons are confined and oscillate repeatedly. During each round trip, the right-handed circularly polarized (RCP) component experiences greater gain than the left-handed circularly polarized (LCP) component. After hundreds or thousands of round trips of amplification, the small inherent differences are continuously accumulated and amplified, leading to a rapid increase in the output g_{ASE} value, which far exceeds the intrinsic g_{lum} value.’.

Reviewers' comments:

Reviewer #2 (Remarks to the Author):

In the previous round of review, I found the helical self-assembly of the nanoplatelets and their chiroptical properties to be interesting. However, the new set of experiments and simulations raises additional questions, as outlined below. I recommend publication of this paper in Nature Communications after the authors have addressed the following comments.

Comments:

1. The assembly process starting from the initial droplet to dried fibrillar-like assemblies using cryo-TEM was demonstrated using NRs. However, the major focus of this work is the self-assembly of NPLs. It would be interesting to see the formation process of superlattices of NPLs.

Response: We thank the reviewer for the insightful suggestion. It is generally difficult to obtain high-contrast image under cryo-TEM, due to the surrounding amorphous ice matrix of the sample. For this reason, we chose NRs as a representative sample to investigate the assembly pathway, as their greater thickness yields much clearer contrast and distinct morphology in frozen solvent. Given that NPLs and NRs share a similar anisotropic nature, and considering that NPLs ultimately form even longer superlattices, we deduce that the NPLs follow a self-assembly process that is fundamentally similar to that of the NRs.

Action: We have added this discussion in the revised manuscript 'Given that NPLs and NRs share a similar anisotropic nature, and considering that NPLs ultimately form even longer superlattices, we deduce that the NPLs follow a self-assembly process that is fundamentally similar to that of the NRs'.

2. To probe the NPLs stacking and gradual rotation along the long axis of fibrillar structure, 3D reconstruction based on scanning TEM and 3D FFT images are provided. However, it is difficult to observe rotations of the NPLs from these images.

Response: We thank the reviewer for this comment. Beside these images, we also provided a video that shows the stacking model of NPLs, as attached below and in Supplementary Movie 1. To further demonstrate this behavior, we have extracted sequential frames from this movie at 0 s, 3 s, 6 s, and 9 s (Figure R1). These frames

clearly illustrate the gradual change in the projected lateral size of the NPLs as the stacking model rotates, confirming their twisted stacking pattern.

Supplementary Movie 1.mp4

Movie R1. 3D reconstruction video of the superlattices based on electron tomography (also seen in Supplementary Movie 1).

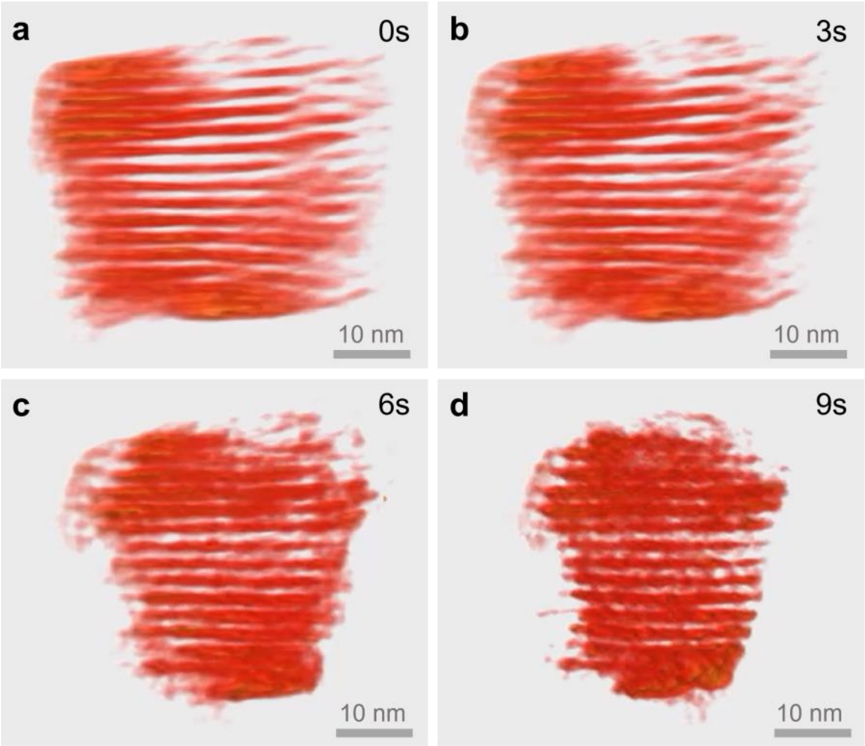

Figure R1. Sequential frames from this movie at (a) 0 s, (b) 3 s, (c) 6 s, and (d) 9 s.

Action: We have added Figure R1 as Supplementary Fig. 25 in the Supporting Information.

3. Electromagnetic simulations were performed to show CPL from the stacked NPLs. The simulated CPL and g-factor do not contain units (figure R9), although the g-factor was compared with the experimental value (figure 3e). Also, CD and CPL plots in measured scale may be provided.

Response: We thank the reviewer for these suggestions. The simulated CPL intensity in Figure R9 (or Figure 7c) is normalized for clarity, and therefor does not include units. Similarly, the g-factor are calculated using the formula $g = 2 \times \frac{I_L - I_R}{I_L + I_R}$, and are consequently dimensionless as well. To avoid misunderstanding, we have revised the y-axis label of Figure 6c which now reads as ‘simulated CPL (normalized)’.

Furthermore, all original CD/CPL data plotted with scale and unit (Figure R2) have been provided in the Supporting Information.

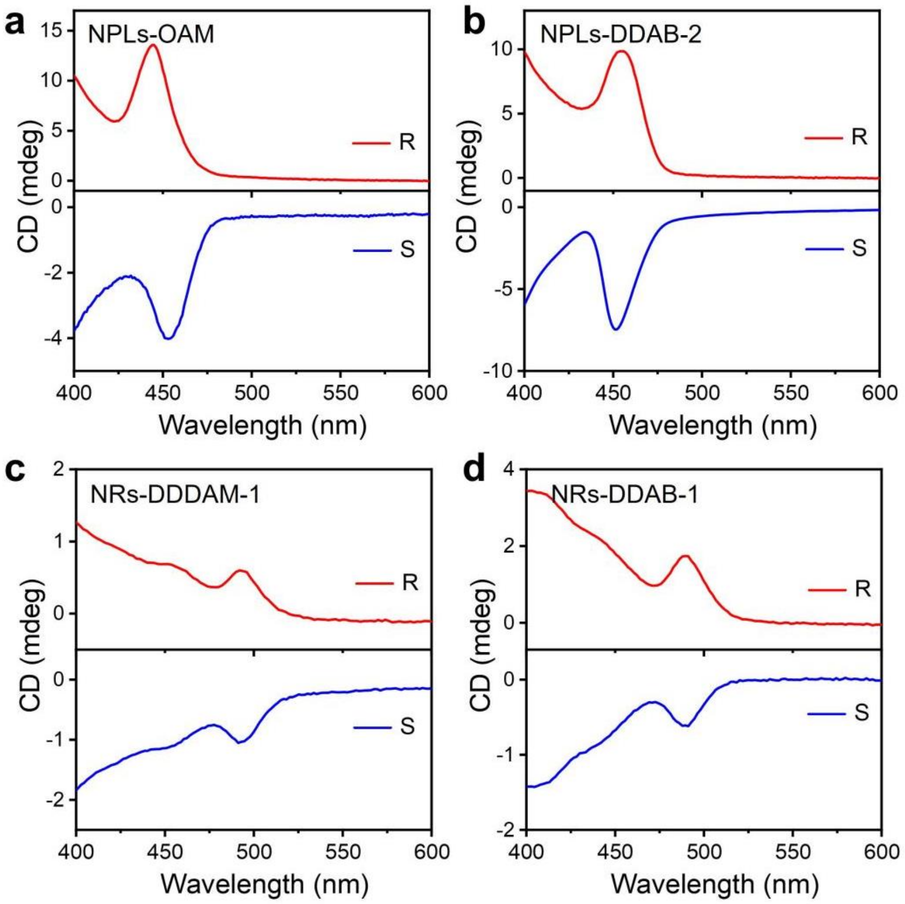

Figure R2. (a) Circular dichroism spectra of NPLs-OAM superlattices. (b) Circular dichroism spectra of NPLs-DDAB-2 superlattices. (c) Circular dichroism spectra of NRs-DDDAM-1 superlattices. (d) Circular dichroism spectra of NRs-DDAB-1 superlattices. All the intensity values plotted have not been normalized. The red curves represent the R-SLs, while the blue curves represent the S-SLs.

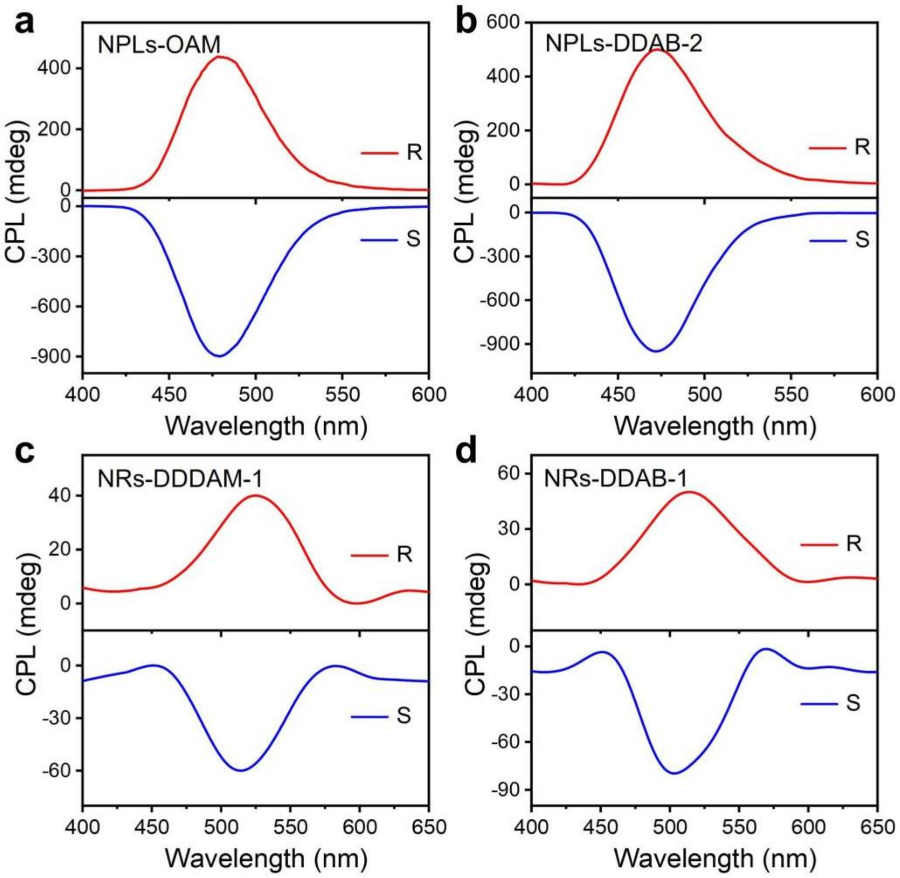

Figure R3. (a) Circularly polarized photoluminescence spectra of NPLs-OAM superlattices. (b) Circularly polarized photoluminescence spectra of NPLs-DDAB-2 superlattices. (c) Circularly polarized photoluminescence spectra of NRs-DDDAM-1 superlattices. (d) Circularly polarized photoluminescence spectra of NRs-DDAB-2 superlattices. All the intensity values plotted have not been normalized. The red curves represent the R-SLs, while the blue curves represent the S-SLs.

Action: We have revised the label of y axis in Figure 6c. We have added Figure R2-3 as Figure S18-19 in the Supporting Information.

4. Optical simulations were performed to demonstrate the optical microcavities effect of 1D superlattices (Figure 6i). However, the mechanism of CPL g-factor vs ASE g-factor still remain unclear.

Response: We thank the reviewer for the comment. In the absence of chiral ligands or other chiral templates, both the CPL and ASE are attributed to the twisted stacking pattern of the NPLs within the 1D superlattices. The optical simulations also support this light-matter interaction mechanism: incident light is converted into circular polarized luminescence (CPL) after passing through the left-handed (LH) or right-

handed (RH) stacked NPLs (Figure 7a-d). Regarding the ASE, left- and right-handed circularly polarized light experience distinct gain and loss within the chiral microcavity structure. This difference is further amplified through the multi-feedback process, leading to a significantly enhanced g-factor of ASE (*Angewandte Chemie,* **2025,** *137(2):* e202415092;).

Action: We have added the following discussion in the manuscript:

‘In the absence of chiral ligands or other templates, both the CPL and ASE are attributed to the twisted stacking pattern of the NPLs within 1D superlattices’

and cited the related references (*Angewandte Chemie*, **2025**, *137(2)*: e202415092;) as ref-64.

5. The interparticle spacing for the vertical direction was calculated using SAXS and spacing along the lateral direction was calculated using TEM. The interparticle spacings appear to be different for the vertical and lateral directions.

Response: We thank the reviewer for the comment. Indeed, the interparticle spacing along the vertical direction determined by SAXS is ~2.3-3.0 nm, whereas the spacing along the lateral direction determined by TEM is larger, in the range of ~3.7-4.2 nm. This difference can be directly observed in TEM (Figure 2c-o): the NPLs are densely stacked along the vertical direction of the 1D superlattices, but relatively loosely packed with neighboring NPLs along the lateral direction. This discrepancy arises because the lateral size distribution of the NPLs is less uniform than their thickness distribution. Ideally, the NPLs exhibit no variation in thickness. Therefore, dense packing is only achieved in the thickness direction.

Action: We have added this discussion in manuscript ‘the NPLs are densely stacked along the vertical direction of the 1D superlattices, but relatively loosely packed with neighboring NPLs along the lateral direction. This discrepancy arises because the lateral size distribution of the NPLs is less uniform than their thickness distribution. Ideally, the NPLs exhibit no variation in thickness. Therefore, dense packing is only achieved in the thickness direction.’

6. The authors stated that the stress induced by the ligands under locked condition led to the twisting of the NPLs. However, the interparticle distance between the NPLs depends on the thickness of the NPLs which in tern has an effect on the locking of the NPLs. Hence, the stress may not be the same for all the NPLs which will affect the degree of rotation. Additionally, the self-assembly of cadmium selenide nanoplatelets [Science Advances 2017, 3 (9), e1701483] did not show chirality in spite of ligand induced stress.

Response: We thank the reviewer for these comments.

(1) We agree that NPLs with different thicknesses are subject to different levels of ligands-induced stress. Indeed, thicker NRs based superlattices exhibit a g_{abs} factor of the order of 10^{-4} (Figure S14), whereas thinner NPLs based superlattices exhibit a g_{abs} factor of 10^{-3} (Figure S15). The higher g_{abs} factor suggests stronger twisting of the NPLs within the superlattice, likely resulting from greater ligands-induced stress among thinner NPLs.

(2) Regarding the previously reported CdSe NPLs helices (Science Advances, 2017, 3(9), e1701483), these helices are well dispersed and do not interact with each other (Figure R3). Consequently, left- and right-handed helices are present in nearly equal amounts, resulting in a racemic system that exhibits no chiroptical response. In contrast, in our work, the densely aligned superlattices enable an autocatalysis effect (*Science*, **1990**, *250(4983)*, 975 - 976.): an initial small fluctuation in one handedness promotes the formation of the same handedness, ultimately leading to macroscopic chiral symmetry breaking.

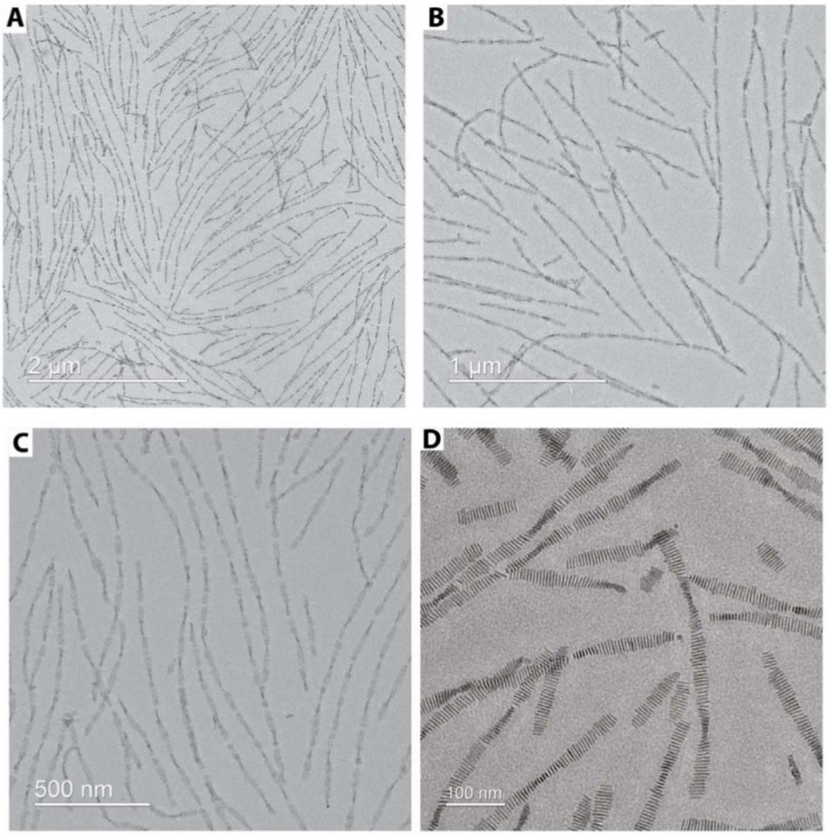

Figure R3. Screenshot of Figure 2 from Science Advances 2017, 3 (9), e1701483.

Action: We have added the corresponding discussion in manuscript: Regarding the previously reported CdSe NPLs helices, these helices are well dispersed and do not interact with each other. Consequently, left- and right-handed helices are present in nearly equal amounts, resulting in a racemic system that exhibits no chiroptical

response. In contrast, in our work, the densely aligned superlattices enable an autocatalysis effect: an initial small fluctuation in one handedness promotes the formation of the same handedness, ultimately leading to macroscopic chiral symmetry breaking.

‘We have cited the reference (*Science*, **1990**, *250(4983)*, 975‑976.) as ref-56.

7. The polarization measurements were carried out with a polarizer behind the emission lens to control the polarization direction. This eventually gives the emission polarization. My specific query was with the polarization behavior with excitation polarizer and nanoplatelets orientation.

Response: We thank the reviewer for the suggestion.

(1) Due to space limitation of our experimental platform, building a setup capable of delivering excitation light with controllable polarization poses significant difficulties.

(2) In the present system, the ground-state optical absorption behavior of the NPLs is largely unaffected by their spatial configuration and orientation. Therefore, we believe that using polarized or unpolarized excitation light would not alter the polarization characteristics of the fluorescence emission.

Action: None.

8. The dissymmetric factor obtained from CD is in the range of 10^{-3} to 10^{-4} while the dissymmetric factor from CPL appears to be ~ 10^{-2}. Generally, the dissymmetric factor for CPL remains smaller than the dissymmetric factor of CD. Please comment.

Response: We thank the reviewer for the comment. CD measures ground-state chiroptical absorption differences, while CPL originates from excited-state emission. Generally, the dissymmetric factor for CPL (g_{lum}) remains smaller than that for CD (g_{abs}). However, in a few systems such as strongly chiral superstructures, exciton coupling, or chiral ligand-based superstructures, only the electronic ground state contributes to CD measurement, while the CPL process encompasses a broader range of excited transitions relevant for luminescence, which can lead to higher dissymmetry factors due to the cumulative effects of multiple contributing transitions (*Angewandte Chemie,* **2025**, *137(13)*: e202423272).

Action: We have added the corresponding discussion: ‘only the electronic ground state contributes to CD measurement, while the CPL process encompasses a broader range of excited transitions relevant for luminescence, which can lead to higher dissymmetry factors due to the cumulative effects of multiple contributing transitions (*Angewandte Chemie,* **2025**, *137(13)*: e202423272)’.

We have cited the references (*Angewandte Chemie*, **2025**, *137(13)*: e202423272.) as

ref-31.

9. $CsPbI_3$ NPLs (Nature Photonics, 2024, 18, 586 – 594); CsPbBr3 NPLs (Nano Lett. 2023, 23, 2148-2157); CsPbBr3 NPLs (J. Am. Chem. Soc. 2015, 137, 16008-16011); CsPbBr3 NPLs (Light: Science & Applications (2025) 14:336); CsPbBr3 NPLs (Nano Lett. 2020, 20, 1808-1818); CsPbBr3 NPLs (J. Phys. Chem. Lett. 2019, 10, 1300-1305) exhibited similar self-assemblies of halide perovskite NPLs. Please include these references.

Response: We thank the reviewer for the suggestion. We have cited the above-mentioned references in the introduction and discussion sections of the revised manuscript. These citations have significantly strengthened our introduction to the background of perovskite NPLs self-assembly.

Action: We have cited the references as ref-17、ref-22、ref-23、ref-24、ref-25 and ref-26.(*Nature Photonics*, **2024**, *18*, 586 – 594; *Nano Lett.* **2023**, *23*, 2148-2157; *J. Am. Chem. Soc.* **2015**, *137*, 16008-16011; *Light: Science & Applications* (**2025**) *14*:336; *Nano Lett.* **2020**, *20*, 1808-1818; *J. Phys. Chem. Lett*. **2019**, *10*, 1300-1305.)
